# DOT1L inhibition reveals a distinct subset of enhancers dependent on H3K79 methylation

**DOI:** 10.1038/s41467-019-10844-3

**Published:** 2019-06-26

**Authors:** Laura Godfrey, Nicholas T. Crump, Ross Thorne, I-Jun Lau, Emmanouela Repapi, Dimitra Dimou, Alastair L. Smith, Joe R. Harman, Jelena M. Telenius, A. Marieke Oudelaar, Damien J. Downes, Paresh Vyas, Jim R. Hughes, Thomas A. Milne

**Affiliations:** 1 MRC Molecular Haematology Unit, MRC Weatherall Institute of Molecular Medicine, NIHR Oxford Biomedical Research Centre Haematology Theme, Radcliffe Department of Medicine, University of Oxford, Oxford, OX3 9DS UK; 2 MRC WIMM Centre for Computational Biology, MRC Weatherall Institute of Molecular Medicine, Radcliffe Department of Medicine, University of Oxford, Oxford, OX3 9DS UK; 3 MRC Molecular Haematology Unit, MRC Weatherall Institute of Molecular Medicine, Radcliffe Department of Medicine, University of Oxford, Oxford, OX3 9DS UK; 4 Department of Haematology, Oxford University Hospitals NHS Foundation Trust, Oxford, OX3 9DU UK

**Keywords:** Gene regulation, Chromatin structure, Transcriptional regulatory elements, Molecular medicine

## Abstract

Enhancer elements are a key regulatory feature of many important genes. Several general features including the presence of specific histone modifications are used to demarcate potentially active enhancers. Here we reveal that putative enhancers marked with H3 lysine 79 (H3K79) di or trimethylation (me2/3) (which we name H3K79me2/3 enhancer elements or KEEs) can be found in multiple cell types. Mixed lineage leukemia gene (*MLL*) rearrangements (MLL-r) such as MLL-AF4 are a major cause of incurable acute lymphoblastic leukemias (ALL). Using the DOT1L inhibitor EPZ-5676 in MLL-AF4 leukemia cells, we show that H3K79me2/3 is required for maintaining chromatin accessibility, histone acetylation and transcription factor binding specifically at KEEs but not non-KEE enhancers. We go on to show that H3K79me2/3 is essential for maintaining enhancer-promoter interactions at a subset of KEEs. Together, these data implicate H3K79me2/3 as having a functional role at a subset of active enhancers in MLL-AF4 leukemia cells.

## Abstract (web summary)

Histone 3 lysine 79 is mono (me1), di (me2), or tri (me3) methylated by the methyltransferase DOT1L. Here the authors reveal a group of enhancers defined by H3K79me2/3 which regulates enhancer-promoter interactions and other key enhancer features in MLL-AF4 leukemia cells.

## Introduction

Enhancers are key regulatory elements that contribute to gene expression. They function in part by acting as docking sites for transcription factors (TFs), which activate appropriate target genes over long distances through mechanisms that have not been fully elucidated^1^. A key attribute of many enhancers is that they come into close physical proximity with the promoter when active^2–5^. Enhancers have the capacity to drive gene expression in a tissue-specific manner, so the same DNA sequence can be inactive in one tissue, but fully functional when the correct tissue-specific TFs are expressed^6^. Because of this, identifying active, tissue-specific enhancers can be challenging as many genes have more than one enhancer, which may be active only in specific cell types.

One common way to identify putative enhancers is by using genome-wide chromatin profiling. In general, active enhancers display characteristics including an open chromatin conformation and histone modifications such as H3 lysine 4 monomethylation (H3K4me1) and H3 lysine 27 acetylation (H3K27ac)^7–10^. Attempts to use other features to subclassify enhancers resulted in the identification of super-enhancers^11–13^.

Disruptor of telomeric silencing 1-like (DOT1L) is the only known methyltransferase for H3 lysine 79 methylation (H3K79me)^14^. H3K79 can be mono (me1), di (me2), or tri (me3) methylated, all of which are deposited by DOT1L^14^. H3K79me2/3 is mainly found within the body of active genes and is associated with transcription elongation^14–18^. The precise role of H3K79me2/3 in promoting transcription elongation is unknown, but one model suggests that H3K79me2/3 functions in part by inhibiting histone deacetylase activity and by preventing the formation of H3 lysine 9 trimethylated (H3K9me3) repressive domains^19^. Some additional support for this model comes from recent evidence that KDM2B may act as a histone demethylase for H3K79me2/3 causing recruitment of SIRT1 and gain of H3K9me3^20^. Several reports have also suggested that H3K79me2/3 can also be used as a marker of functionally important active enhancers^21–23^. However, the functional significance of H3K79me2/3 at enhancers has not been established.

H3K79me has also been shown to be important in human disease. In particular, H3K79me2/3 is an important driver of leukemogenesis, mainly in a rare subset of leukemias caused by rearrangements of the mixed lineage leukemia (*MLL*) gene^17,24–28^. The most common MLL rearrangements (MLL-r) are chromosome translocations that fuse MLL in-frame with over 120 genes creating novel fusion proteins, most frequently MLL-AF4^29^. MLL-AF4 is a major cause of incurable acute lymphoblastic leukemia (ALL) in infants and children^29–31^, and increased H3K79me2 or H3K79me3 correlates with increased transcription of MLL-AF4-bound genes^26,32–36^, likely due to aberrant recruitment of DOT1L^15,23,24,37^. The highly specific DOT1L inhibitor, EPZ-5676 (pinometostat), has moderate clinical activity in patients^38^ and has been extensively characterized in numerous studies as an excellent tool to study the molecular function of DOT1L^19,39^.

Past studies have shown that H3K79me3 is a marker of active *Drosophila* enhancers during embryogenesis^22^ and H3K79me2 has been found at a subset of super-enhancers in MLL-r leukemia cells^23^. Although there has been some indication that H3K79me2 and H3K79me3 may have similar binding profiles in mammalian cells^18,35^, most work has focused on the association of H3K79me2 with active gene bodies in MLL-r leukemia cells^18,24,26,35^, and it is unclear whether there is a distinction between the two methylation states. Where H3K79me2/3 enhancer function has been studied, the focus has been mainly on H3K79me2, but the role of this mark has only been partially elucidated^21–23^. For example, it is unclear to what extent H3K79me2/3 is found at enhancers genome-wide in mammalian cells and whether it contributes to enhancer function.

In this study, we perform a genome-wide analysis in multiple cell types and discover a large set of enhancers that are marked with H3K79me2/3, which we term H3K79me2/3 enhancer elements (KEEs). In MLL-AF4 cells, we demonstrate that H3K79me2/3 plays a functional role at these enhancers with loss of H3K79me2/3 leading to a reduction of H3K27ac, chromatin accessibility, and TF binding specifically at KEEs. This perturbation leads to a downregulation of transcription and a disruption of interactions between KEEs and the promoter of the regulated gene. Together, these results define a distinct set of enhancers in MLL-AF4 cells, which are dependent on H3K79me2/3 to maintain an active, open chromatin configuration, facilitating enhancer–promoter interactions that contribute to the transcriptional activity of the regulated gene.

## Results

### H3K79me2/3 marks a set of putative enhancers (KEEs)

**Fig. 1:**
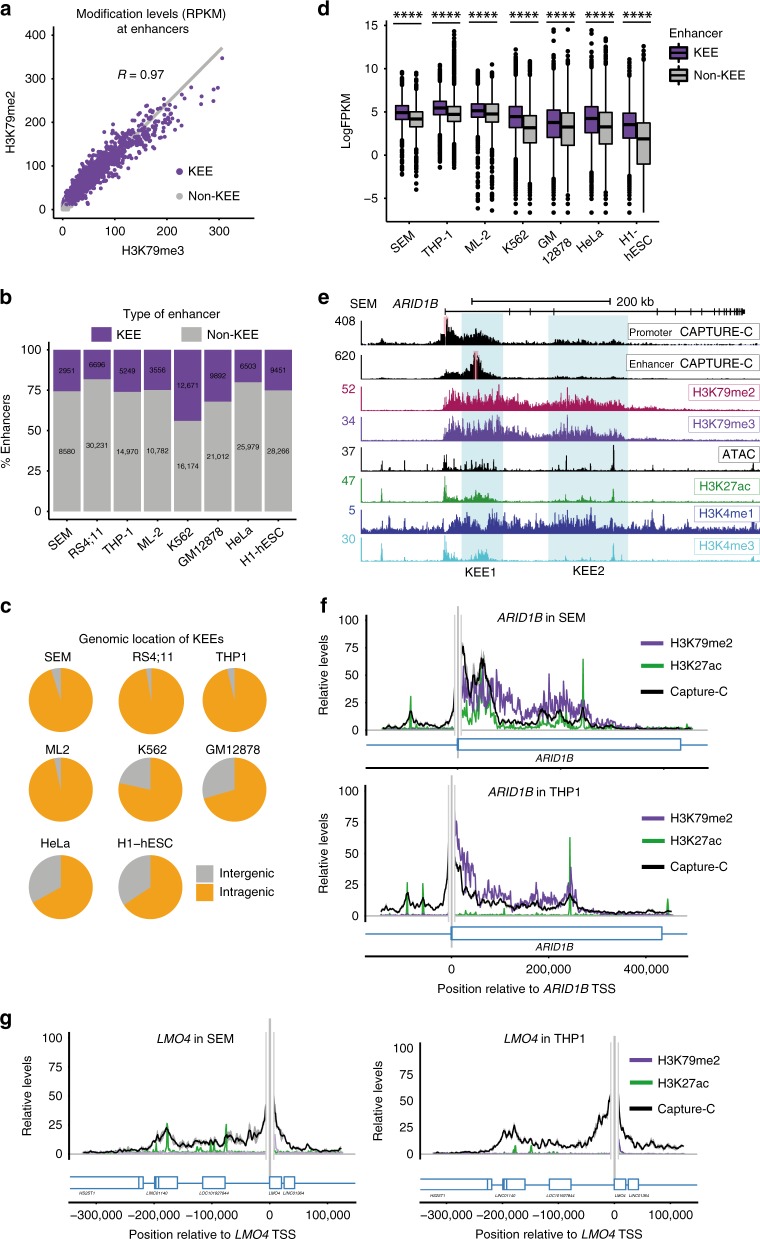
H3 lysine 79 methylation 2/3 (H3K79me2/3) marks a subset of enhancers. **a** Correlational analysis between H3K79me2 chromatin immunoprecipitation-sequencing (ChIP-seq) and H3K79me3 ChIP-seq reads in SEM cells at H3K79me2/3 enhancer elements (KEEs) (purple) and non-KEEs (gray), based on one biological replicate. **b** Proportion of predicted enhancers, which are KEEs (purple) or non-KEEs (gray) in different cell types, based on ChromHMM analysis. **c** Genomic location, either intragenic (orange) or intergenic (gray), of KEEs in different cell types. **d** Gene expression (log FPKM (fragments per kilobase of transcript, per million) values) of KEE genes (purple) and non-KEE genes (gray) in different cell types. Most data were based upon one replicate; SEM and THP1 data were based on three replicates. ****p < 0.0001, using a Mann–Whitney *U* test. Box plots show interquartile range; center line represents the median value. **e** Capture-C, ChIP-seq, and assay for transposase-accessible chromatin using sequencing (ATAC-seq) at *ARID1B* performed in SEM cells. Blue boxes indicate KEE clusters 1 and 2. Red bars indicate location of Capture-C probes. Capture-C track is mean of three biological replicates; ATAC-seq is a representative track of five biological replicates. **f**, **g** Overlay of Capture-C with H3K79me2 and H3 lysine 27 acetylation (H3K27ac) ChIP-seq at *ARID1B* and *LMO4* in SEM and THP1 cells. Gray bars represent location of Capture-C probe, ±1 kb exclusion zone. Shaded area around Capture-C signal represents 1 s.d. See also Supplementary Fig. 1

Since past work suggested that either H3K79me2^23^ or H3K79me3^22^ could be found at a subset of active enhancers, we initially wanted to determine if either modification could be used to categorize enhancers in SEM (MLL-AF4) leukemia cells. Using the automated chromatin-state discovery package ChromHMM^40,41^, we classified putative enhancers based on the presence of H3K27ac and H3K4me1, and subcategorized them using either H3K79me2 or H3K79me3 chromatin immunoprecipitation-sequencing (ChIP-seq). H3K79me2 and H3K79me3 ChIP-seq signal at enhancers displayed a very strong correlation (Fig. 1a, *R* = 0.97) and both marks independently identified an almost identical set of enhancers (Supplementary Fig. 1a). For the sake of convenience, we refer to H3K79me2/3-marked enhancer elements as KEEs and non-H3K79me2/3-marked enhancer elements as non-KEEs. Importantly, although KEEs overlap with some super-enhancers^11–13^ in SEM cells, most KEEs are not super-enhancers and many super-enhancers are not KEEs (Supplementary Fig. 1a, b, Supplementary Data 1).

We then broadened our analysis to assess the distribution of putative KEEs across a wide range of different cell types including different human leukemia cell lines and human embryonic stem cells (hESCs). Using ChromHMM with lab-generated and publicly available H3K27ac, H3K4me1, and H3K79me2 ChIP-seq datasets, we identified putative enhancers that were marked with H3K79me2 (Fig. 1b). Enhancer lengths were comparable in all cell types (Supplementary Fig. 1c), with median lengths of 1 and 0.8 kb for KEEs and non-KEEs, respectively, in SEM cells. Notably, the majority of KEEs were intragenic across all cell types, although there was also a small but variably-sized proportion of intergenic KEEs (Fig. 1c).

We next asked if the presence of a KEE had any impact on transcription of the associated gene. Since genes without H3K79me2/3 generally have lower expression^18,26,35^, we excluded non-H3K79me2/3-marked genes from our analysis to avoid this bias. We used the common approach of annotating each enhancer to the nearest gene^7^, and using this to label genes as KEE or non-KEE associated (Supplementary Data 2). Many genes were associated with both KEEs and non-KEEs. For the purposes of our analysis, KEE genes were defined by proximity to one or more KEEs (regardless of non-KEE association) and non-KEE genes were defined by proximity to one or more non-KEEs, but no KEEs.

Interestingly, we found that KEE-associated genes generally have higher levels of expression compared to non-KEE genes (Fig. 1d). Recent work from our lab has shown that a subset of important MLL-AF4 gene targets, termed spreading targets, are distinguished by broad binding of MLL-AF4, high levels of H3K79me2, and elevated gene expression^35^. We therefore asked whether the presence of H3K79me2 at KEEs is a consequence of MLL-AF4 spreading into intragenic regions. Whilst MLL-AF4 spreading targets are enriched for KEEs compared to non-spreading MLL-AF4 genes, the vast majority of KEE genes are not spreading genes (Supplementary Fig. 1d, e). Taken together, these data suggest that KEEs are functionally distinct enhancers from super-enhancers and MLL-AF4 spreading gene targets.

### KEEs are marked by increased enhancer–promoter interactions

So far, our analysis has revealed the existence of putative H3K79me2/3-marked enhancers (KEEs) in multiple cell types including hESCs and leukemia cells. However, histone marks alone do not indicate whether an enhancer is functional. One key attribute of many active enhancers is that they tend to be located physically closer to their target promoter^1^. To identify enhancer–promoter interactions at specific genes, we used next-generation (NG) Capture-C, a high-resolution chromosome conformation technique^42^. Due to the potential therapeutic importance of H3K79me2/3 in MLL-r cells, we decided to focus our analysis on KEE function in three different MLL-r leukemia cell lines: SEM, RS4;11 (MLL-AF4 leukemia), and THP1 (MLL-AF9 leukemia). NG Capture-C performed in triplicate was used to analyze promoter interactions for up to 61 different genes, including both KEE- and non-KEE-associated genes (Supplementary Data 3). We found KEEs that interacted with promoters in SEM, RS4;11, and THP1 cells (Fig. 1e and Supplementary Fig. 1f-o). For example, the *ARID1B* gene contains two intragenic KEE clusters, marked with H3K79me2, H3K79me3, H3K4me1, and H3K27ac, and regions of open chromatin as demonstrated by assay for transposase-accessible chromatin using sequencing (ATAC-seq) in SEM cells (Fig. 1e, shaded regions). Capture-C from the viewpoint of the *ARID1B* promoter and KEE1 in SEM cells revealed reciprocal interactions with both KEE clusters and the promoter (Fig. 1e).

Although KEEs were similar between SEM and RS4;11 cells, they varied in THP1 cells both in terms of size and promoter interaction frequency, for example, at *ARID1B*, *BCL11A*, and *JMJD1C* (Fig. 1f and Supplementary Fig. 1f-h, compare blue shaded regions). Conversely, the KEEs in *CDK6* are similar in all three cell types (Supplementary Fig. 1h–k). One possible explanation for these differences could be the pattern of H3K79me2 enrichment across the gene body, as the H3K79me2 level at KEEs seems to correlate with the strength of the Capture-C signal (Fig. 1f and Supplementary Fig. 1f–k, m). This can be observed at the *ARID1B* gene, where regions of high H3K79me2 and high H3K27ac show a correlation with increased frequency of promoter interaction in both SEM and THP1 cells (Fig. 1f). This appears to be a common attribute of KEEs (Supplementary Fig. 1i–k, m).

In contrast to the KEE gene examples, the major site of interaction with the *LMO4* promoter is a distal non-KEE where the Capture-C profile mirrors the H3K27ac profile (Fig. 1g and Supplementary Fig. 1l, o). Interestingly, the promoter of *BCL2* interacts with a 3′ non-KEE (Supplementary Fig. 1n–o), which has previously been identified as an enhancer^34,43^. However, the peak of interaction partially overlaps with a region of H3K79me2/3, potentially making this enhancer both a KEE and non-KEE (Supplementary Fig. 1n-o). Both the non-KEE of *BCL2* and of *LMO4* display very similar interaction profiles in all three cell types (Supplementary Fig. 1o).

Taken together, our data indicate that H3K79me2/3 marks a subset of mainly intragenic enhancers. At the genes we have tested, Capture-C reveals an enriched interaction frequency between promoters and KEEs (as well as non-KEEs). Our results also indicate that KEE activity may vary between different cell types. This is consistent with what is known about enhancer function, where activity is often cell type specific, usually driven by the specific repertoire of TFs present.

### KEEs are functional enhancers

**Fig. 2:**
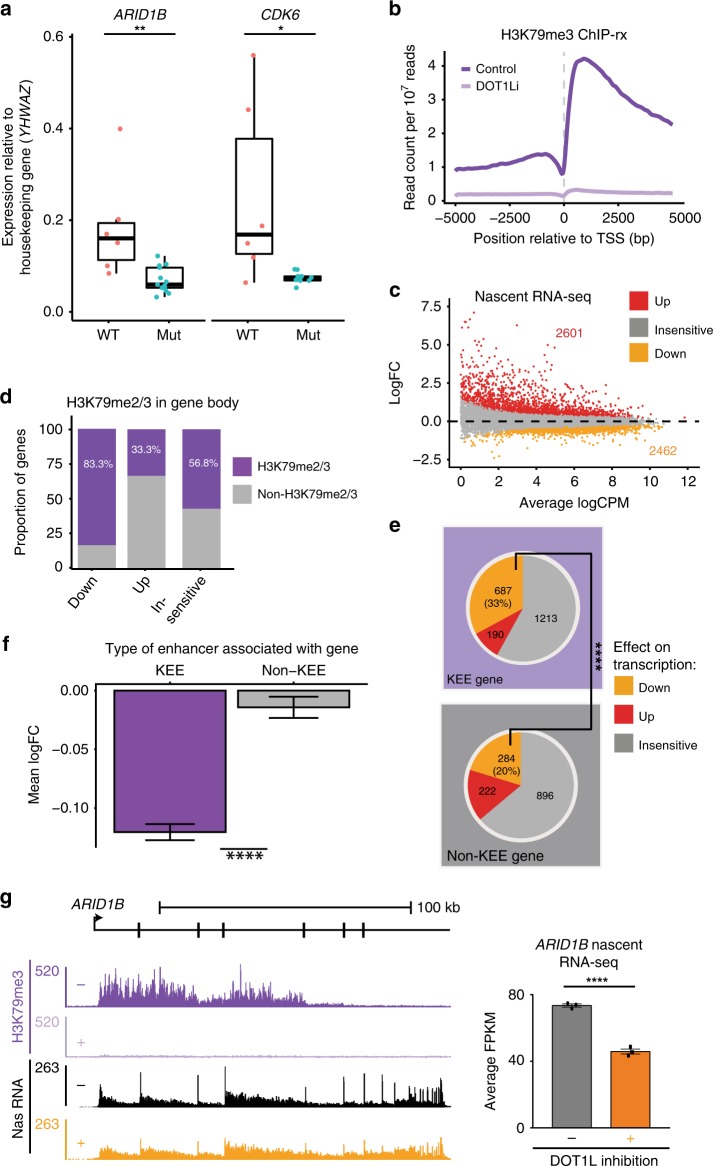
Loss of H3 lysine 79 methylation 2/3 (H3K79me2/3) at enhancers leads to a reduction in transcription at H3K79me2/3 Enhancer Element (KEE) genes. **a** Gene expression in wild-type SEM compared to *ARID1B* and *CDK6* enhancer mutant clones, normalized to the housekeeping gene *YWHAZ*. Each point represents a biological replicate. Mann–Whitney *U* test, *p < 0.01, **p < 0.05 based upon nine biological replicates. Box plots show interquartile range; center line represents the median value. **b** Metaplot of H3K79me3 reference-normalized ChIP-seq (ChIP-rx) signal at all transcriptional start sites (TSSs) following DOT1Li (EPZ-5676) treatment (lilac) compared to control treatment (purple) in SEM cells. **c** MA plot of nascent RNA-seq data showing differential gene expression (up (red), down (orange), and insensitive (gray)) in SEM cells following DOT1Li. Differential expression = FDR (false discovery rate) < 0.05 from three biological replicates. **d** Proportion of differentially expressed genes and insensitive genes, which are directly marked with H3K79me2/3 in the gene body in SEM cells. **e** Proportion of KEE genes and non-KEE genes, which are upregulated (red), downregulated (orange), or insensitive (gray) following DOT1Li by nascent RNA sequencing (****p < 0.0001, Fisher’s exact test), *n* = 3. **f** Mean log FC of H3K79me2/3-marked genes associated with a KEE (purple) or non-KEE (gray) following DOT1Li, from nascent RNA-seq data (****p < 0.0001, Fisher’s exact test, *n* = 3). Error bars represent s.e.m. **g** Left: H3K79me3 ChIP-rx tracks showing control (−, purple) and DOT1Li (+, lilac) samples, and nascent RNA-seq tracks showing control (−, black) and DOT1Li (+ , orange) samples at *ARID1B* in SEM cells. Right: Bar chart displaying mean FPKM at *ARID1B* in control (−, black) and DOT1Li (+, orange) conditions. ****FDR < 0.0001, *n* = 3. Error bars represent s.e.m. Source data are provided as a source data file. See also Supplementary Fig. 2

Although many functional enhancers have been shown to interact with the promoter of the gene they regulate, interaction alone does not necessarily prove that enhancers are functional. To determine whether any of the KEEs we identified were functional, we used two different approaches. First, we cloned fragments of the *ARID1B* and *JMJD1C* KEEs and the *BCL2* non-KEE into a luciferase construct containing a minimal promoter. We used the *BCL2* non-KEE as a positive control as it has previously been identified as a putative 3′ enhancer^34^, and deletion in an endogenous context has recently been shown to reduce *BCL2* expression^43^. When transfected into 293T cells, the *ARID1B*, *JMJD1C*, and *BCL2* sequences increased luciferase activity by approximately two-fold relative to the promoter-alone vector (Supplementary Fig. 2a). This suggests that these regions of DNA can function as an enhancer in a simple luciferase assay. Second, to determine if KEEs promote gene activation within their endogenous context, we focused on two specific KEE genes: *ARID1B* and *CDK6*. We targeted guide RNAs (gRNAs) to delete a portion of these putative enhancers in SEM cells using CRISPR/Cas9 and analyzed at least nine independent clones of each. We found that expression of *ARID1B* and *CDK6* was significantly reduced compared to wild type, consistent with these regions displaying enhancer activity (Fig. 2a). Deletions were characterized in one clone of each, showing intronic KEE deletions (Supplementary Fig. 2b). Together, these results show that the specific KEEs tested are functionally important for maintaining the expression of *ARID1B* and *CDK6*.

Since specific KEEs are associated with enriched enhancer–promoter interactions at highly expressed genes, we wanted to better understand whether H3K79me2/3 contributes to KEE function. We used the small-molecule DOT1L inhibitor EPZ-5676^39^ to diminish H3K79me2/3 genome wide in SEM (MLL-AF4) leukemia cells. Treatment with 2 µM EPZ-5676 led to a near-complete global loss of H3K79me2/3 as measured by reference-normalized ChIP-seq (ChIP-rx^44^), western blot, and ChIP-qPCR (quantitative PCR) (Fig. 2b and Supplementary Fig. 2c-d). Following DOT1L inhibition (DOT1Li), we used nascent RNA-seq data in SEM cells^35^ to identify genes dependent on H3K79me2/3 for transcription and identified 2601 upregulated and 2462 downregulated genes (Fig. 2c). Downregulated genes were more likely to be marked by H3K79me2/3 within the gene body (Fig. 2d), consistent with a role for H3K79me2/3 in active transcription^18^. In aggregate, KEE-associated genes were significantly more sensitive to DOT1Li, with 33% (687) downregulated, compared to 20% (284) of non-KEE genes (Fig. 2e–f and Supplementary Fig. 2e). For example, at *ARID1B* loss of H3K79me2/3 is coupled with downregulation of transcription (Fig. 2g). This effect was validated at *ARID1B* and other gene targets with quantitative reverse transcription PCR (qRT-PCR) of total RNA levels (Supplementary Fig. 2f).

Many non-KEE genes that are highly enriched for H3K79me2/3 in the gene body were also downregulated upon DOT1Li, such as *PBX3* (Supplementary Fig. 2g), highlighting the likely non-enhancer transcription roles for H3K79me2/3. In contrast, the non-KEE gene *LMO4* is not affected by DOT1Li, despite the presence of H3K79me2/3 in the gene body (Supplementary Figs. 1o, 2g). As discussed above, *BCL2*, which contains both KEE and non-KEE regions (Supplementary Fig. 1n), has been shown to be primarily regulated by the downstream 3′ non-KEE^34,43^. Loss of H3K79me2/3 produced a subtle reduction in transcription of *BCL2* following DOT1Li (Supplementary Fig. 2f, g). The high levels of H3K79me2/3 across the length of the *BCL2* gene body, which do not correlate with the potential KEE region, suggest that loss of this modification may have effects on transcription independent of enhancer function (Supplementary Fig. 2h).

Taken together, these results indicate that in addition to the potential role of H3K79me2/3 in regulating transcription independent of enhancers, the association of a gene with a KEE increases the likelihood that transcription will be dependent on H3K79me2/3. We therefore asked whether loss of H3K79me2/3 could be associated with a specific reduction of enhancer characteristics at KEEs.

### Maintenance of H3K27ac at KEEs requires H3K79me2/3

**Fig. 3:**
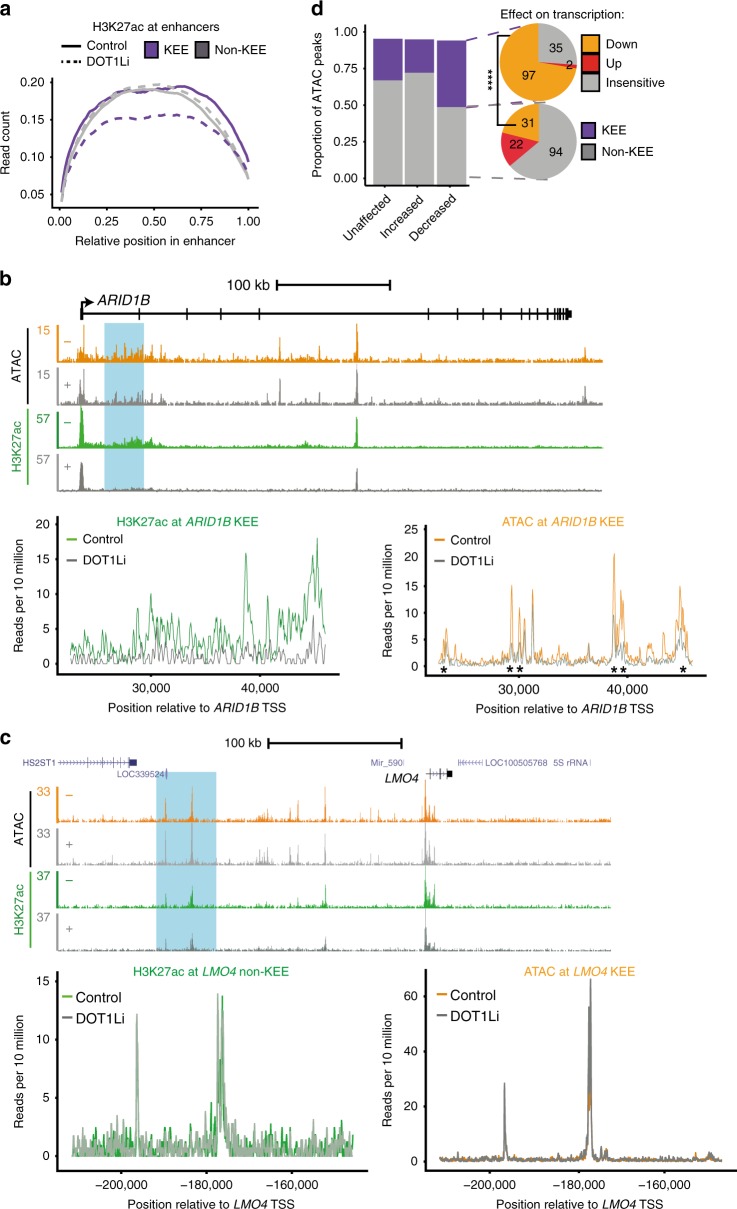
Loss of H3 lysine 79 methylation 2/3 (H3K79me2/3) leads to reduction in H3 lysine 27 acetylation (H3K27ac) and chromatin accessibility at H3K79me2/3 Enhancer Elements (KEEs). **a** Metaplot of H3K27ac chromatin immunoprecipitation-sequencing (ChIP-seq) signal across KEEs (purple) or non-KEEs (gray) in control (solid line) and DOT1Li (dashed line) SEM cells, *n* = 1. **b**, **c** Upper: assay for transposase-accessible chromatin using sequencing (ATAC-seq) and H3K27ac ChIP-seq at *ARID1B* and *LMO4* in control (−, orange/green) and DOT1Li (+, gray) SEM cells. Blue boxes highlight KEE cluster 1 region of *ARID1B* and non-KEE region associated with *LMO4*. Lower: Overlay of H3K27ac (left) and ATAC (right) signal at *ARID1B* KEE1 and *LMO4* non-KEE in control (green/orange) and DOT1Li (gray). Asterisks represent significant decreases in ATAC signal following DOT1Li, false discovery rate (FDR) < 0.05. **d** Left: Proportion of increased, decreased, or unchanged ATAC peaks found within a KEE (purple) or non-KEE (gray). Right: Proportion of decreased ATAC peaks within a KEE (upper) or non-KEE (lower) that are associated with transcriptionally downregulated (orange), upregulated (red), or insensitive (gray) gene. ****P < 0.0001, Fisher’s exact test, *n* = 5. See also Supplementary Fig. 3

If H3K79me2/3 contributes to KEE function, we might expect DOT1Li to result in a reduction in TF binding and chromatin accessibility, as well as a loss of key histone modifications at KEEs. We used ATAC-seq and H3K27ac ChIP-seq following DOT1Li in SEM cells to investigate this^45^. Strikingly, when we compared H3K27ac changes at enhancers genome wide, a clear decrease was observed exclusively at KEEs (Fig. 3a), although there were no observed global changes in H3K27ac (Supplementary Fig. 3a). The changes in H3K27ac are exemplified at DOT1Li-sensitive KEE genes, *ARID1B*, *BCL11A*, and *CDK6*, where H3K27ac levels are strongly diminished at the KEEs following DOT1Li (Fig. 3b, blue shaded region and lower left trace and Supplementary Fig. 3b). In contrast, at *LMO4* and *FOXO3* there are no clear changes in H3K27ac levels at the non-KEE regions (Fig. 3c, blue shaded region and lower left trace and Supplementary Fig. 3b). DOT1Li followed by H3K79me2 and H3K27ac ChIP-qPCR in RS4;11 and K562 (a BCR-ABL cell line) cells at specific KEEs displayed a similar H3K79me2-H3K27ac interdependence (Supplementary Fig. 3c–d). Together, our results show that in several cell types, maintenance of H3K27ac specifically at KEEs is dependent on H3K79me2/3.

ATAC-seq analysis in SEM cells following DOT1Li revealed that the majority of accessible chromatin regions were not affected by a loss of H3K79me2/3 (Supplementary Fig. 3e and Supplementary Data 4). ATAC peaks that decreased following DOT1Li were associated with a higher level of H3K79me3, indicating that loss of H3K79me3 at these loci may be responsible for the reduced accessibility (Supplementary Fig. 3f). We observed an enrichment of decreased chromatin accessibility at KEE genes compared to non-KEE genes in general (Fig. 3d and Supplementary Fig. 3g) and specifically at *ARID1B* vs. *LMO4* (Fig. 3b–c, lower right panels). In general, decreased ATAC-seq peaks were associated with higher levels of H3K79me2/3 and downregulation of transcription following DOT1Li (Supplementary Fig. 3f–h, Supplementary Data 4). To understand whether these changes in chromatin accessibility are relevant to enhancer function, we compared decreased ATAC peaks at KEEs or non-KEEs to changes in transcription of the associated genes. Strikingly, 73% of decreased ATAC peaks within KEEs correlated with downregulation of transcription of the corresponding genes, in contrast to only 21% of decreased peaks at non-KEEs (Fig. 3d, right). We also found a greater reduction of H3K27ac at decreased ATAC peaks within KEEs compared to non-KEEs (Supplementary Fig. 3i), consistent with the preferential loss of histone acetylation within KEEs following DOT1Li (Fig. 3a). We note that DOT1Li-sensitive ATAC peaks in KEEs show a higher level of H3K27ac compared to those within non-KEEs (Supplementary Fig. 3i), in contrast to the similar H3K27ac levels across KEEs and non-KEEs (Fig. 3a). This is likely because the ATAC peaks take up only a small proportion of the enhancers.

Additionally, we performed ATAC-seq in RS4;11 cells and observed similar reductions in chromatin accessibility at KEEs, exemplified by *ARID1B* (Supplementary Fig. 3j, shaded regions) but not at the *LMO4* non-KEE (Supplementary Fig. 3j). We also revisited the KEE/non-KEE region of *BCL2* where we observed no change in H3K27ac or ATAC signal in SEM or RS4;11 cells (Supplementary Fig. 3j–k), further suggesting that the *BCL2* enhancer functions mostly independently of H3K79me2/3.

Taken together, our data suggest that loss of H3K79me2/3 reduces H3K27ac levels and chromatin accessibility specifically at many KEEs but not non-KEEs. This suggests that H3K79me2/3 stabilizes H3K27ac at KEEs, potentially maintaining enhancer function and promoting transcription.

### TF binding at KEEs requires H3K79me2/3

Our results so far indicate that H3K79me2/3 is required at KEEs to maintain chromatin accessibility and H3K27ac levels. To explore this mechanism, we investigated whether TF binding at KEEs was disrupted by a loss of H3K79me2/3.

**Fig. 4:**
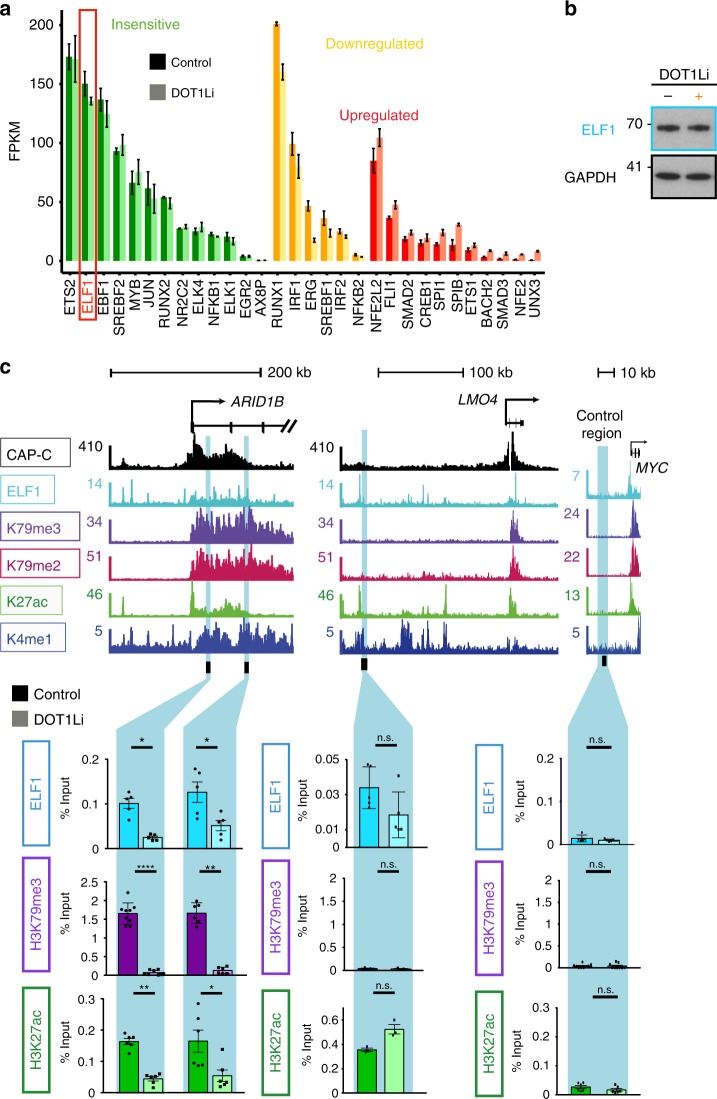
Loss of H3 lysine 79 methylation 2/3 (H3K79me2/3) leads to a reduction in ELF1 binding at H3K79me2/3 Enhancer Elements (KEEs). **a** Expression level of transcription factors whose motifs are enriched at KEEs compared to the rest of the genome, sorted into categories of transcriptionally insensitive (green), downregulated (orange), and upregulated (red) following DOT1Li. ELF1 is highlighted in a red box. **b** Western blot analysis of ELF1 and GAPDH following salt-soluble protein extraction of SEM cells in control (−) and DOT1Li (+) conditions. Representative of three biological replicates. **c** Chromatin immunoprecipitation-quantitative PCR (ChIP-qPCR) at *ARID1B* KEEs, *LMO4* non-KEE and negative control locus for ELF1, H3K79me3, and H3 lysine 27 acetylation (H3K27ac in control (darker shade) and DOT1Li (lighter shade) conditions. *n* = 6, error bars represent s.e.m. *P < 0.05, **p < 0.005, ****p < 0.0001 using a Mann–Whitney *U* test. n.s. not significant. Source data are provided as a source data file. See also Supplementary Fig. 4

To identify possible TFs that could be bound to KEEs, we performed a motif analysis and filtered the most highly enriched motifs by expression level in SEM cells. We categorized these TFs into three categories, either transcriptionally insensitive, downregulated or upregulated following DOT1Li, and focused on the insensitive category (Fig. 4a). ChIP-seq experiments revealed that ELF1, MYB, and RUNX2 bound to the *ARID1B* KEEs (Supplementary Fig. 4a), suggesting that these TFs may be important for KEE function.

As a proof of principle, we focused on ELF1, which is not affected by DOT1Li at either the transcriptional or protein level (Fig. 4a–b). We hypothesized that if loss of H3K79me2/3 at KEEs created an environment that was generally repressive for TF binding, we should observe reduced ELF1 binding at KEEs upon DOT1Li. Indeed, we found a reduction of ELF1 binding as measured by ChIP-qPCR at the KEEs of *ARID1B*, *JMJD1C*, and *BCL11A* following DOT1Li but not at the non-KEEs of *LMO4*, *SPI1*, or at one region of the *BCL2* non-KEE (Fig. 4c and Supplementary Fig. 4b–e). However, it is worth noting that another primer pair used at the *BCL2* non-KEE did display a reduction in ELF1 binding (Supplementary Fig. 4e). Loss of ELF1 binding was coupled with a reduction in H3K79me2, H3K79me3, and H3K27ac specifically at several KEE example genes (Fig. 4c and Supplementary Fig. 4b–e). Interestingly, no changes in H3K4me1, another enhancer-specific modification, were observed (Supplementary Fig. 4b–e). Although we observed a modest trend towards increases in the repressive modification H3K9me3, this appeared to be a more general effect occurring at KEEs, non-KEEs, and the non-enhancer control region (Supplementary Fig. 4b–e).

Taking these data together, we propose that the increases in H3K9me3 following DOT1Li do not explain the specific changes in TF binding at KEEs. However, there is clear link between H3K79me2/3-dependent H3K27ac, chromatin accessibility, and TF binding at KEEs, consistent with the increased transcriptional sensitivity of KEE genes to DOT1Li.

### Maintenance of KEE–promoter interactions requires H3K79me2/3

**Fig. 5:**
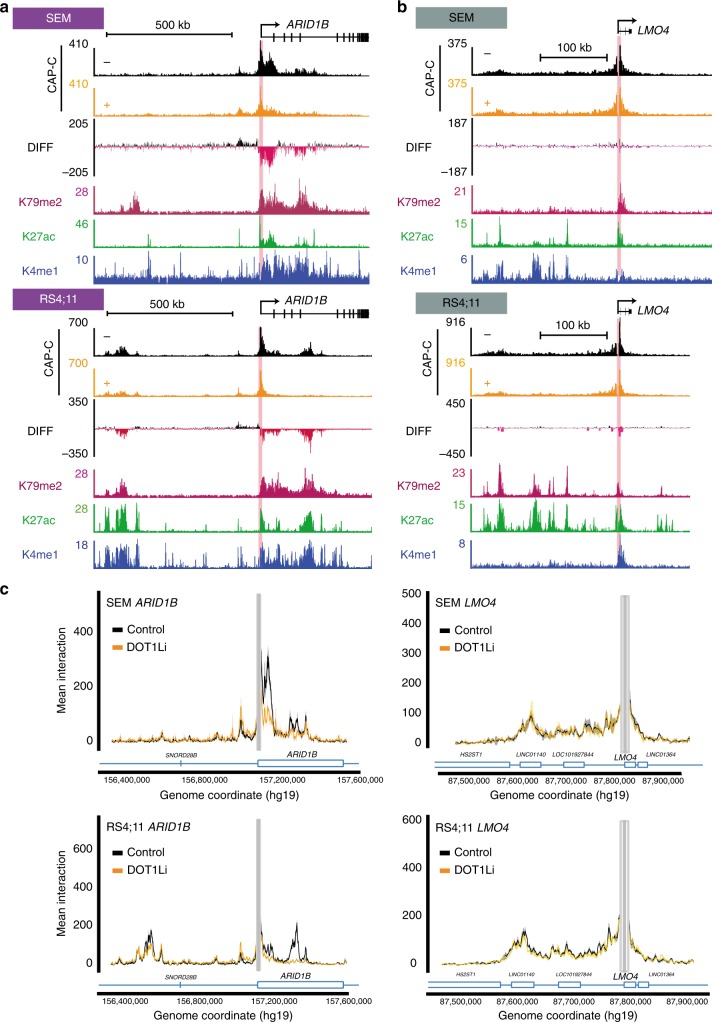
Loss of H3 lysine 79 methylation 2/3 (H3K79me2/3) leads to a reduction in H3K79me2/3 Enhancer Element (KEE)–promoter interactions. **a** Capture-C (*n* = 3) at *ARID1B* in SEM and RS4;11 cells in control (black) and DOT1Li (orange) conditions. Differential track shows average difference in Capture-C signal following DOT1Li, from three biological replicates. Loss of interaction (pink), and gain of interaction (black). H3K79me2, H3 lysine 27 acetylation (H3K27ac), H3K4me1 chromatin immunoprecipitation-sequencing (ChIP-seq) at *ARID1B*. Red line represents location of Capture-C probe. **b** Capture-C (*n* = 3) and ChIP-seq at *LMO4* in SEM and RS4;11 cells, as in **a**. **c** Overlay of control (black) and DOT1Li (orange) Capture-C signal (average of three biological replicates) from the *ARID1B* and *LMO4* promoters in SEM and RS4;11 cells. Gray bars represent location of Capture-C probe, ±1 kb exclusion zone. Shaded area around Capture-C signal represents 1 s.d. See also Supplementary Fig. 5

**Fig. 6:**
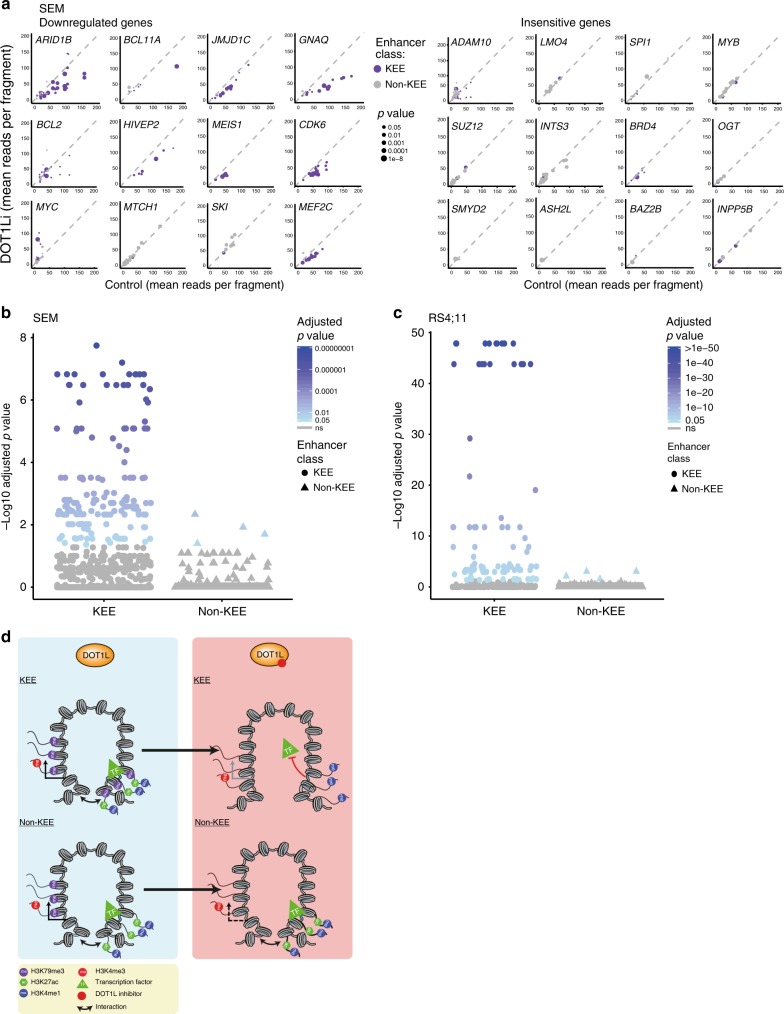
Loss of H3 lysine 79 methylation 2/3 (H3K79me2/3) leads to widespread reductions in H3K79me2/3 Enhancer Element (KEE)–promoter interactions. **a** Enhancer–promoter Capture-C interaction frequencies in Control (*x*-axis) and DOT1Li (*y*-axis) SEM cells. Left: Interactions with DOT1Li-downregulated genes; right: DOT1Li-insensitive genes. Each point represents the interaction of a KEE (purple) or non-KEE (gray) with the indicated gene promoter. Size of dot corresponds to significance of change in interaction, using a Wilcoxon’s rank test. See Supplementary Table 1 for list of *p* values. **b**, **c** Statistical analysis of the significance of the change in enhancer–promoter Capture-C interactions following DOT1Li in SEM and RS4;11 cells. Each point represents the interaction of a KEE (circle) or non-KEE (triangle) with a gene promoter. Holm–Bonferroni adjusted *p* values were calculated following a Wilcoxon’s rank test (*n* = 3). **d** Model for the role of H3K79me2/3 at KEEs. Loss of H3K79me2/3 at KEEs following DOT1Li leads to a reduction in H3K27ac, transcription factor (TF) binding and enhancer–promoter interactions. This is associated with a reduction in gene expression of the KEE-associated gene. Loss of H3K79me2/3 within the gene body leads to no changes in non-KEE enhancer activity or enhancer–promoter interaction. A reduction in transcription of the non-KEE gene may be observed due to enhancer-independent roles of H3K79me2/3 in the gene body. See also Supplementary Fig. 6

As we found that loss of H3K79me2/3 at KEEs interferes with TF binding, we hypothesized this might perturb enhancer function by disrupting enhancer–promoter interactions. To address this, we used NG Capture-C^42^ combined with DOT1Li, in three biological replicates of three independent cell types: SEM (MLL-AF4), RS4;11 (MLL-AF4), and THP1 (MLL-AF9). Capture-C oligos were designed to the promoter of the gene, so that all interactions between the promoter and any potential active enhancers would be captured, with some reciprocal interactions validated using enhancer probes (Supplementary Data 3). Overall domain profiles were not affected following DOT1Li, suggesting that domain boundaries are maintained following a loss of H3K79me2/3 (Fig. 5 and Supplementary Figs. 5, 6a–b). However, DOT1Li induces a strong perturbation of interactions between KEEs and the promoter within these domains in both SEM and RS4;11 cells, and to a lesser extent in THP1 cells. In contrast, non-KEE-promoter interactions are largely unaffected. This is highlighted in several KEE examples at *ARID1B*, *BCL11A*, *CDK6*, and *JMJD1C* (Fig. 5a, c and Supplementary Figs. 5a–c, 6a), which show strong H3K79me2/3-dependent KEE–promoter interactions, and *LMO4*, which shows non-KEE–promoter interactions that are insensitive to DOT1Li (Fig. 5b–c and Supplementary Fig. 6a–b). In the more complex example of *BCL2*, which appears to contain both KEE and non-KEE enhancers, no significant disruption of enhancer–promoter interactions overall is observed in SEM, RS4;11, or THP1 cells (Supplementary Figs. 5d, 6a, b). However, there are some smaller fragments of KEE loss of interaction as visualized in the bubble plots (Fig. 6a and Supplementary Fig. 6c), indicating that more subtle regulation may be occurring at this locus. Interestingly, differences between cell types were observable. At *ARID1B*, a unique intergenic KEE approximately 600 kb upstream from the promoter, which is not found in SEM cells, interacts with the promoter of *ARID1B* in RS4;11 cells, and this interaction is perturbed following DOT1Li (Fig. 5a, c).

To demonstrate the effect of H3K79me2/3 loss more systematically, we compared promoter–enhancer interactions at 61 genes in control and DOT1Li conditions. The majority of significant differences, most of which were coupled with a decrease in transcription, were observed between a KEE and a promoter in SEM and RS4;11 cells (Fig. 6a–c and Supplementary Fig. 6c). Very few promoter-non-KEE interactions were significantly affected by DOT1Li in either SEM or RS4;11 cells (Fig. 6b, c and Supplementary Data 5). Although we did not observe as many significant changes in Capture-C signal in THP1 cells, we did observe significant decreases at the KEE genes *CDK6* and *JMJD1C*, which were also identified in SEM and RS4;11 cells (Supplementary Fig. 6d), and no significant change between the non-KEE and promoter of *BCL2* was observed (Supplementary Fig. 6a). Taken together, this suggests that H3K79me2/3 plays a role in maintaining KEE–promoter interactions and KEE function, which contributes to the transcriptional activation of KEE genes. We therefore propose that H3K79me2/3 contributes to the function of a subset of enhancers by stabilizing TF binding and histone acetylation, which in turn facilitates enhancer–promoter interactions and transcription of target genes (Fig. 6d).

## Discussion

In this study, we demonstrate the existence of a subset of putative enhancers (KEEs) that are marked with H3K79me2 and/or H3K79me3 in multiple cell types. We have identified putative KEEs in multiple cell types including hESCs, and our results demonstrate that H3K79me2/3 is critical for the function of some enhancers in MLL-r cells.

In yeast, H3K79me2 and H3K79me3 display different distribution patterns^46^, but past work in mammalian cells has suggested that these two modifications may overlap^18,35^. Our analyses demonstrate a strong correlation between H3K79me2 and H3K79me3, suggesting that at least in MLL-AF4 cells they are likely functionally equivalent. The H3K79me3 antibody we use (Diagenode C15410068, lot no. A246-0040, recommended by BLUEPRINT for ChIP-seq) shows a high degree of specificity for H3K79me3 in a dot blot analysis (http://antibody.uni-saarland.de/antibody/112/Diagenode_company/H3K79me3/), and the H3K79me2 antibody we use has been shown to be specific for H3K79me2 in ChIP experiments^18^. This indicates that our H3K79me2 and H3K79me3 ChIP-seq results are not a consequence of antibody cross-reactivity, as has been noted for a different H3K79me3 antibody^18^. However, since the nature of the antibody–epitope interaction differs between applications, we cannot completely rule out the possibility of cross-reactivity in our ChIP experiments.

Treatment with the DOT1L inhibitor EPZ-5676 reduces H3K79me2/3 globally and we show that this leads to a loss of enhancer activity specifically at KEEs in MLL-AF4 cells. While DOT1L is the only enzyme known to catalyze H3K79 methylation, it is possible that DOT1L has additional methylation targets, and this may contribute to the effects we observe following the loss of DOT1L methyltransferase activity.

The loss of H3K79me2/3 at KEEs is associated with a localized reduction of H3K27ac, chromatin accessibility and TF binding, and reduced frequency of KEE–promoter interactions coupled with transcriptional downregulation. This contrasts with our observations at non-KEE-regulated genes that are insensitive to DOT1Li where loss of H3K79me2/3 in the gene body can disrupt transcription without impacting enhancer function. We also observe a striking correlation between H3K79me2/3 patterns of enrichment and H3K27ac/Capture-C profiles, although it is not clear if the presence of H3K79me2/3 is required to establish these enhancers. Certainly, our results demonstrate that KEEs are dependent on H3K79me2/3 for maintenance of enhancer function in MLL-AF4 cells.

In addition to our work in MLL-AF4 leukemia cells, we observe some evidence for an interdependence between H3K79me2/3 and H3K27ac levels at KEEs in K562 cells, and some subtle reductions in interactions between KEEs and promoters in THP1 cells. This subtle reduction may be due to the SEM-based Capture-C probe set used, which may not be optimal in THP1 cells. The smaller size of some of the KEEs in THP1 cells compared to SEM and RS4;11 cells (see Supplementary Fig. 1f, h) supports this. However, it is also possible that H3K79me2/3 plays a more pivotal role at KEEs in MLL-AF4 cells. Future studies in other cell types and with other Capture-C probe sets will help distinguish these possibilities.

Histone modifications, such as H3K4me1 and H3K27ac, as well as chromatin accessibility, have been useful for identifying putative enhancers^7,10,22,47^. It is striking that we observe a specific reduction of H3K27ac at KEEs, correlating with a loss of enhancer–promoter interactions following DOT1Li, but do not see changes in H3K4me1. This might suggest that H3K79me2/3 acts downstream of H3K4me1 deposition in enhancer function and may indicate that, following loss of H3K79me2/3 and H3K27ac, KEEs revert to a more poised state^8^ (Fig. 6d).

The functional significance of the loss of enhancer–promoter interactions following DOT1Li is a key question arising from our work. Although there are varying reports on the importance of enhancer proximity for promoter function^2–4,48–50^, many enhancers appear to require close association with the promoter in order to promote transcription^1^. Recent work has suggested that enhancers may act as hubs for the accumulation of factors that generate distinct regulatory domains^51,52^, and this could potentially stabilize enhancer–promoter interactions. From this perspective, it is interesting that loss of H3K79me2/3 at KEEs results in reduced TF binding. This raises the possibility that the role of H3K79me2/3 at KEEs is to provide an opportunity for TFs to bind, perhaps by creating a more open chromatin state. Thus, the presence of H3K79me2/3 alone does not create an enhancer; the underlying DNA sequence that dictates TF binding would ultimately determine functionality in a specific cell type. This is supported by the fact that at least one of the *ARID1B* KEEs can function as an enhancer in a luciferase assay in 293T cells, in which the enhancer is unlikely to be chromatinized and is probably active due to unobstructed binding of sequence-specific TFs. Cell-type-specific differences in the repertoire of TFs present may explain why some KEEs that are active in SEM and RS4;11 B-ALL cells are much less active in THP1 AML cells. Like any enhancer sequence, KEEs need to be considered in a cell-type-specific manner to determine their functionality.

How could an increase in H3K79me2/3 levels promote an open chromatin configuration? H3K79 is found in the nucleosome core, but methylation of this residue is thought to have only subtle structural effects and is not likely to disrupt the nucleosome itself^53^. Past observations have demonstrated that H3K79me2/3 inhibits the histone deacetylase SIRT1, maintaining histone acetylation and preventing increases in repressive histone modifications^19^. This is consistent with the loss of H3K27ac we observe specifically at KEEs following DOT1Li. However, our work shows a more general, presumably untargeted, increase in H3K9me3 at both KEEs and non-KEEs following DOT1Li. The fact that changes in H3K27ac, but not H3K9me3, correlate with loss of enhancer activity suggests that H3K27ac may mediate increased chromatin accessibility and TF binding, rather than functioning by indirect antagonism of repressive modifications. Notably, many TFs are known to recruit the H3K27 acetyltransferases CBP/p300^54^, suggesting the potential for positive feedback to stabilize enhancer features, dependent on H3K79me2/3 to prevent histone deacetylation.

In summary, we propose that H3K79me2/3 at enhancers is necessary for maintaining an open chromatin conformation and promoting histone acetylation, possibly via inhibition of SIRT1. If the underlying DNA sequence contains TF binding sites, this provides an opportunity for binding and activation of enhancer function, including an increased frequency of interaction with the target gene promoter. In a particular cell type, this would only happen if appropriate TFs were expressed, meaning that the repertoire of H3K79me2/3-dependent enhancers will differ from tissue to tissue. Whilst our demonstration of functional behavior of KEEs here is limited mainly to MLL-AF4 leukemia cells, the fact that a similar proportion of enhancers are marked with H3K79me2 in other cell types suggests that a role for this modification in enhancer function may be more generally applicable.

## Methods

### Cell culture and cell lines

SEM (MLL-AF4 B cell ALL line)^55^ and ML-2 (MLL-AF6 AML cell line)^56^ cells were purchased from DSMZ (www.cell-lines.de) and cultured in Iscove’s modified Dulbecco’s medium media with 10% fetal bovine serum (FBS) and Glutamax. RS4;11 (MLL-AF4 B cell ALL line), THP1 (MLL-AF9 AML cell line), and K562 (BCR-ABL CML cell line) cells were purchased from ATCC (www.lgcstandards-atcc.org) and cultured in RPMI-1640 with 10% FBS and Glutamax. The 293T cells were purchased from ATCC and cultured in Dulbecco’s modified Eagle’s medium (DMEM) with 10% FBS and Glutamax.

### EPZ-5676 treatment

Cells were seeded at 0.3 × 10^6^ cells/ml. SEM and RS4;11 cells were treated with 2 µM EPZ-5676 or with 0 µM (dimethyl sulfoxide (DMSO) only) control. THP1 cells were treated with 5 µM EPZ-5676. Cells were grown for 7 days, with a change of media and EPZ-5676 at days 3 and 6, when the cells were counted and split to 0.5 × 10^6^ cells/ml and 0.7 × 10^6^ cells/ml, respectively. At day 7, EPZ-5676-treated cells were harvested and processed for downstream applications.

### Salt-soluble protein extraction and histone acid extraction

A salt-soluble protein extraction was performed on 1 × 10^6^ SEM cells using BC300 (20 mM Tris-HCl, pH 7.5; 20% glycerol; 300 mM KCl; 5 mM EDTA) + 0.5% NP-40 + Protease inhibitor cocktail (Roche). Following this, histone proteins were extracted from nuclear pellets with 0.4 M HCl and acetone precipitation. Samples were then processed for western blot analysis.

### Western blot analysis

Western blot analysis was performed using BC300 extracted protein samples or on acid extracted histones loaded onto either 4–12% (for non-histone proteins) or 12% (histones) bis-tris gel and blotted using polyvinylidene fluoride membrane for 1 h at 100 V using a tris-glycine transfer buffer. Uncropped versions of western blots performed can be found in the Source data file.

### Antibodies used for western blotting, ChIP-qPCR, and ChIP-seq

For ChIP 2 μl of each antibody was used per 10^7^ cells. For western blotting individual dilutions are indicated. Anti-H3K79me3 (Diagenode C15410068, lot no. A246-0040 for ChIP-qPCR, ChIP-seq, and western blotting (1/5000)); anti-H3K79me2 (ChIP-seq, Millipore 04-835); anti-H3K79me2 (ChIP-qPCR, Abcam ab3594); anti-H3K27ac (Diagenode C15410196, lot no. A1723-0041D for ChIP-qPCR, ChIP-seq, and western blotting (1/20,000)); anti-H3K4me1 (ChIP-seq, Diagenode pAB-194-050); anti-H3K4me1 (ChIP-qPCR and western blotting (1/100,000), Abcam ab8895); anti-H3K4me3 (ChIP-seq, Diagenode pAB-003-050); anti-H4 (western blotting (1/100,000), Abcam ab7311); anti-ELF1 (Bethyl A301-443A, lot no. 1 for ChIP-qPCR, ChIP-seq, and western blotting (1/10,000)); anti-RUNX2 (ChIP-seq, Cell Signalling 8486, lot no. 1); anti-MYB (ChIP-seq, Abcam ab177510); anti-CTCF (ChIP-seq, Millipore 07-729); anti-BRD4 (ChIP-seq, Bethyl A301-985A).

### ChIP assays

In brief, fixed samples of up to 1 × 10^8^ cells were sonicated using a Covaris (Woburn, MA,USA) according to the manufacturer’s recommendations. Ab:chromatin complexes were isolated using a mixture of magnetic Protein A and Protein G Dynabeads (Life Technologies) and were washed three times with a solution of 50 mM HEPES-KOH, pH 7.6, 500 mM LiCl, 1 mM EDTA, 1% NP-40, and 0.7% Na deoxycholate. Following a Tris-EDTA wash, samples were eluted, treated with RNase A and proteinase K, and purified using a Qiagen PCR purification kit. DNA was quantified by qRT-PCR, with ChIP samples normalized using input chromatin^57^ (Supplementary Table 6 for list of qPCR primers used). For ChIP-seq, DNA libraries were generated using the NEB Next Ultra DNA library preparation kit for Illumina (Cat no. E7370). Samples were sequenced by paired-end sequencing using a NextSeq 500 (Illumina). Also described in refs. ^33,35,36^.

### ChIP-rx

Fixed *Drosophila melanogaster* S2 cells were added at the lysis step of the ChIP to SEM cells at a ratio of 1:4. Following sequencing, reads were mapped to both the hg19 and dm3 genome builds (see Sequence analysis). For additional information on the original protocol see ref. ^44^.

### RNA extraction

Total RNA was extracted using the RNeasy Mini kit (Qiagen). For RT-qPCR RNA was reverse transcribed using SuperScript III (Thermo Fisher) with random hexamer primers, and then quantified using TaqMan qPCR. Gene expression was normalized to the housekeeping gene *YWHAZ*. For details on nascent RNA experimental procedure see Kerry et al. ^35^. Briefly, 1 × 10^8^ THP1 cells were treated with 500 µM 4-thiouridine (4-SU) for 1 h. Cells were lysed with Trizol (Life Technologies) and total RNA was precipitated. 4-SU-incorporated RNA was purified using biotinylation and streptavidin bead pulldown. DNA libraries were prepared from RNA using the NEB Next Ultra Directional RNA library preparation kit for Illumina. Samples were sequenced by paired-end sequencing using a NextSeq 500 (Illumina).

### ATAC-seq

Fifty thousand SEM/RS4;11 cells were harvested and washed in phosphate-buffered saline and resuspended gently in 50 µl cold lysis buffer (10 mM Tris-HCl, pH 7.4; 10 mM NaCl; 3 mM MgCl_2_; 0.1% IGEPAL CA-630). Cells were spun down immediately at 500 × *g* for 10 min at 4 °C. The pellet was resuspended in a transposase reaction mix at 37 °C for 30 min. DNA was purified using a Qiagen MinElute kit as per the manufacturer’s instructions. The DNA fragments were amplified in a PCR reaction with 12 cycles and purified using Qiagen MinElute kit. Samples were sequenced by paired-end sequencing using a NextSeq 500 (Illumina). For additional information on the original protocol see ref. ^45^.

### CRISPR deletions

Three different gRNAs were cloned into separate copies of the pSpCas9-GFP vector for each gene targeted. For *ARID1B*, gRNA 1: GCACAATACTTGGACGGAA, gRNA 2: GAAGTGCGTCTTCCGTTTA, and gRNA 3: TGGGTGTGAGTCACAACAT. For *CDK6*, gRNA 1: GCTGCTTAGCCGTTTTTAA, gRNA 2: AGTACGCATACCTTTGAAT, and gRNA 3: AGGTTTTCCGGATTCCTAT. Plasmids for each gene were co-transfected into SEM cells by electroporation. At 48 h post transfection, cells were GFP sorted and plated onto MethoCult medium to generate clones. Clones were screened by PCR of the targeted region, and deletions were confirmed by Sanger sequencing.

### Luciferase assay

Candidate enhancer regions were amplified from genomic DNA using the primers given in Supplementary Table 6 and cloned into the pGL3-Promoter vector (Promega) containing a modified version of the firefly luciferase coding sequence. The 293T cells were plated at a density of 1 × 10^5^ cells in 2 ml DMEM in a 6-well dish 24 h prior to transfection. Cells were co-transfected with 500 ng of pGL3-Promoter, together with 500 ng pRL-TK, which contains the *Renilla* luciferase gene, using 5 µl Lipofectamine 2000. After 48 h, cells were scraped in 100 µl luciferase assay buffer (25 mM Tris-HCl, pH 8, 2 mM dithiothreitol, 2 mM EDTA, 1% Triton X-100, 10% glycerol) and freeze thawed twice on dry ice to ensure complete cell lysis. The Dual-Glo luciferase assay system (Promega) was used to examine the expression of firefly and *Renilla* luciferase according to the manufacturer’s instructions. Luminescence was analyzed on Fluorstar Optima microplate reader and firefly luciferase luminescence values were normalized to *Renilla* to correct for transfection efficiency.

### Sequence analysis

For ChIP-seq, ChIP-rx, and ATAC-seq, quality control of FASTQ reads, alignment, PCR duplicate filtering, blacklisted region filtering, and UCSC data hub generation was performed using an in-house pipeline (https://github.com/Hughes-Genome-Group/NGseqBasic/releases). Briefly, the quality of the FASTQ files was checked with fastQC, and then mapped using Bowtie against the human genome assembly (hg19). Unmapped reads were trimmed with trim_galore and then mapped again. Short unmapped reads from this step were combined using Flash and then mapped again. PCR duplicates were removed using samtools rmdup, and any reads mapping to Duke blacklisted regions (UCSC) were removed using bedtools. Directories of sequence tags (reads) were generated from the sam files using the Homer tool makeTagDirectory. The makeBigWig.pl command was used to generate bigwig files for visualization in UCSC, normalizing tag counts to tags per 10 million. For ChIP-rx, the normalization factor was adjusted to take into account the ratio of mapped human (hg19) and *Drosophila* (dm3) reads in the bound and input samples. Peaks were called using the Homer tool findPeaks, with the input track provided for background correction, using the -style histone or -style factor options to call peaks in histone modification or TF/ATAC datasets, respectively. Metagene profiles were generated using the Homer tool annotatePeaks.pl. Statistical analysis of differences between ATAC peaks was conducted with Diffbind, using EdgeR. Peaks were considered different between conditions if they had an adjusted *p* value (false discovery rate (FDR)) of <0.05. Motif analysis was conducted using the Homer tool findMotifsGenome.pl using the parameters -size given and -mask.

### RNA-seq and gene expression analysis

Following QC analysis with the fastQC package (http://www.bioinformatics.babraham.ac.uk/projects/fastqc), reads were aligned using STAR^58^ against the human genome assembly (hg19). Reads that were identified as PCR duplicates using Samtools^59^ were discarded. Gene expression levels were quantified as read counts using the featureCounts function from the Subread package^60^ with default parameters. The read counts were used for the identification of global differential gene expression between specified populations using the edgeR package^61^. RPKM (reads per kilobase of transcript, per million) values were also generated using the edgeR package. Genes were considered differentially expressed between populations if they had an adjusted *p* value (FDR) of <0.05.

### Enhancer state identification

Enhancer states were called using ChromHMM^40,41^. Briefly, the genome was subdivided into 200 bp buckets, and each bucket was iteratively assigned to one of 30 states using the following ChIP-seq peak files from SEM cells: H3K4me1, H3K4me3, H3K9ac, H3K27ac, H3K27me3, H3K36me3, and H3K79me2 or H3K79me3. For other cell types, H3K4me1, H3K27ac, and H3K79me2 alone were used. States were then recombined based on the presence/absence of modification peaks. Specifically, KEEs were defined as the presence of H3K4me1, H3K27ac, and H3K79me2 (or H3K79me3). Conversely, non-KEEs were defined as the presence of H3K4me1 and H3K27ac in the absence of H3K79me2 (or H3K79me3). Enhancer buckets <1 kb apart were merged together, and the merged enhancer was labelled a KEE if a KEE bucket was present. Enhancers were labelled intragenic if they overlapped with a gene’s coordinates, or intergenic if not. Enhancers were assigned to the nearest gene, then these assignments were used to label genes as KEE-associated or non-KEE-associated. Genes associated with both KEEs and non-KEEs were labelled as KEE genes.

### Capture-C

A total of 2 × 10^7^ SEM, RS4;11, or THP1 cells were assayed per sample. *Dpn*II-generated 3C libraries were sonicated to a fragment size of 200 bp and Illumina paired-end sequencing adaptors (New England Biolabs, E6040, E7335, and E7500) were added using Herculase II (Agilent) for the final PCR. Indexing was performed in duplicate to maintain library complexity, with libraries pooled after indexing. Capture-C probes targeting promoters were designed as 70 or 120 bp biotinylated DNA oligonucleotides (IDT) using the online CapSequm tool (http://apps.molbiol.ox.ac.uk/CaptureC/cgi-bin/CapSequm.cgi; ref. ^62^) (Supplementary Data 3, list of biotinylated Capture-C oligos). Enrichment was performed with two successive rounds of hybridization, streptavidin bead pulldown (Invitrogen, M270), bead washes (NimbleGen SeqCap EZ), and PCR amplification (Kapa/NimbleGen SeqCap EZ accessory kit v2). The material was sequenced using the Illumina NextSeq platform with 150-bp paired-end reads. Data analysis was performed using an in-house pipeline (https://github.com/Hughes-Genome-Group/CCseqBasicF/releases; ref. ^42^). Capture-C interactions between captured promoters and enhancers were quantified for statistical analysis. Peaks outside of the bounds of Capture-C interaction domains (visually determined using UCSC genome browser) and those on trans chromosomes were removed from the analysis. Peaks within 10 kb of the Capture-C probe hybridization site were also removed. Holm–Bonferroni-adjusted *p* values for each peak were calculated by comparing all of the normalized read counts for each *Dpn*II fragment and all replicates within a peak using a paired Mann–Whitney test for the two treatment conditions (DMSO vs. EPZ-5675 2 µM). For additional information see original protocol^42^.

### Statistical analysis

All statistical analysis was performed using R (v3.3.3) (R Core Team, 2018). Between-group comparisons of continuous variables were performed with the Wilcoxon’s rank-sum test. Contingency table tests were performed with Fisher’s exact test. Exact *p* values can be found in Supplementary Data 6. Capture-C statistics can be found in Supplementary Data 5.

### Reporting summary

Further information on research design is available in the Nature Research Reporting Summary linked to this article.

## Supplementary information

Supplementary Info

Peer Review

Reporting Summary

Description of Additional Supplementary Files

Supplementary Data 1

Supplementary Data 2

Supplementary Data 3

Supplementary Data 4

Supplementary Data 5

Supplementary Data 6

Source Data

## Data Availability

A reporting summary for this Article is available as a Supplementary Information file. All high-throughput data has been deposited in the Gene Expression Omnibus (GEO) under the accession number GSE117865. GEO accession numbers for datasets used from previous publications can be found in Supplementary Table 2. The source data underlying Figs. 2a, 4b, c and Supplementary Fig. 2a, 2c-d, 2f, 3a, 3c-d, 4b-e are provided as a Source Data file. All data are available from the corresponding author upon reasonable request.

## Code availability

For ChIP-seq and ATAC-seq, quality control of FASTQ reads, genome alignment, PCR duplicate filtering, blacklisted region filtering, and UCSC data hub generation was performed using an in-house pipeline: https://github.com/Hughes-Genome-Group/NGseqBasic/releases. Directories of sequence tags (reads) were generated from the sam files using the Homer tool makeTagDirectory. The makeBigWig.pl command was used to generate bigwig files for visualization in UCSC, normalizing tag counts to tags per 10 million. Peaks were called using the Homer tool findPeaks, with the input track provided for background correction, using the -style histone or -style factor options to call peaks in histone modification or transcription factor/ATAC datasets, respectively. Statistical analysis of differences between ATAC peaks was conducted with Diffbind, using the edgeR package.

For RNA-seq analysis, following QC analysis with the fastQC package (http://www.bioinformatics.babraham.ac.uk/projects/fastqc), reads were aligned using STAR. Reads that were identified as PCR duplicates using Samtools were discarded. Gene expression levels were quantified as read counts using the featureCounts function from the Subread package with default parameters. The read counts were used for the identification of global differential gene expression between specified populations using the edgeR package. Capture-C analysis was performed using an in-house pipeline: github.com/Hughes-Genome-Group/CCseqBasicF/releases.
