# Supplementary material for: DOT1L inhibition reveals a distinct subset of enhancers dependent on H3K79 methylation
Source: Nat Commun. 2019 Jun 26;10:2803. doi: 10.1038/s41467-019-10844-3 (PMC6594956; doi:10.1038/s41467-019-10844-3)
Supplement: Supplementary file 1 — Supplementary Info [file 41467_2019_10844_MOESM1_ESM.pdf]

# DOT1L inhibition reveals a distinct subset of enhancers dependent on H3K79 methylation

## Supplementary information

Godfrey et al.

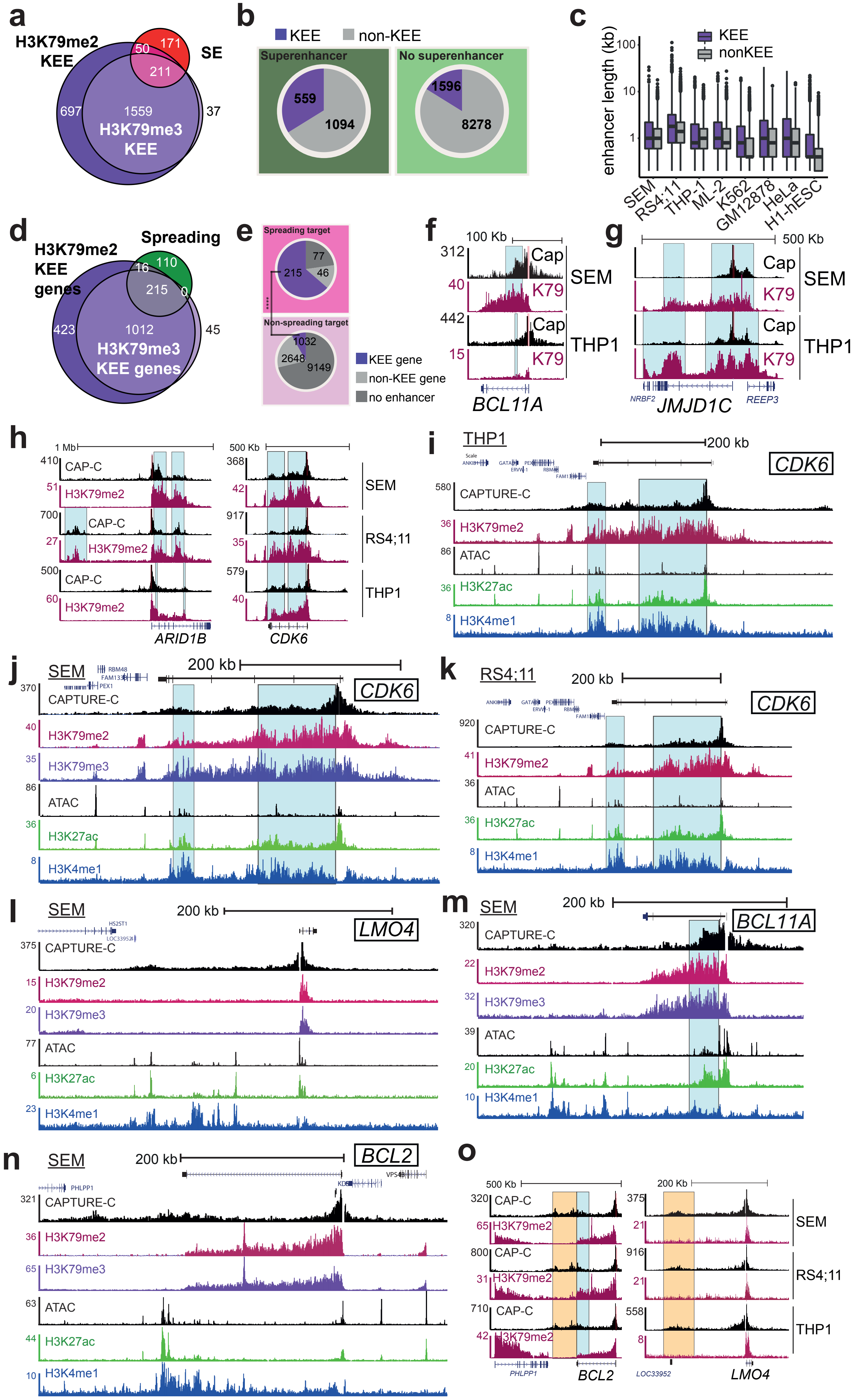

a
H3K79me2 KEE
SE
50
171
211
697
1559
H3K79me3 KEE
37
b
KEE
non-KEE
Superenhancer
No superenhancer
559
1094
1596
8278
c
KEE
nonKEE
enhancer length (kb)
100
10
1
SEM
RS4;11
THP-1
ML-2
K562
GM12878
HeLa
H1-hESC
d
H3K79me2 KEE genes
Spreading
16
110
215
0
423
1012
H3K79me3 KEE genes
45
e
Spreading target
77
215
46
****
Non-spreading target
1032
2648
9149
KEE gene
non-KEE gene
no enhancer
f
100 Kb
312
Cap
40
K79
SEM
442
Cap
15
K79
THP1
BCL11A
g
500 Kb
Cap
SEM
K79
Cap
THP1
K79
NRBF2
JMJD1C
REEP3
h
1 Mb
410
CAP-C
51
H3K79me2
700
CAP-C
27
H3K79me2
500
CAP-C
60
H3K79me2
ARID1B
500 Kb
368
42
917
35
579
40
SEM
RS4;11
THP1
CDK6
i
THP1
200 kb
CDK6
580
CAPTURE-C
36
H3K79me2
86
ATAC
36
H3K27ac
8
H3K4me1
j
SEM
200 kb
CDK6
370
CAPTURE-C
40
H3K79me2
35
H3K79me3
86
ATAC
36
H3K27ac
8
H3K4me1
k
RS4;11
200 kb
CDK6
920
CAPTURE-C
41
H3K79me2
36
ATAC
36
H3K27ac
8
H3K4me1
l
SEM
200 kb
LMO4
375
CAPTURE-C
15
H3K79me2
20
H3K79me3
77
ATAC
6
H3K27ac
23
H3K4me1
m
SEM
200 kb
BCL11A
320
CAPTURE-C
22
H3K79me2
32
H3K79me3
39
ATAC
20
H3K27ac
10
H3K4me1
n
SEM
200 kb
BCL2
321
CAPTURE-C
36
H3K79me2
65
H3K79me3
63
ATAC
44
H3K27ac
10
H3K4me1
o
500 Kb
320
CAP-C
65
H3K79me2
800
CAP-C
31
H3K79me2
710
CAP-C
42
H3K79me2
PHLPP1
BCL2
200 Kb
375
21
916
21
558
8
SEM
RS4;11
THP1
LOC339524
LMO4

**Supplementary Figure 1.** H3K79me2/3 marks a subset of enhancers **(a)** Overlap between KEEs identified using H3K79me2 and H3K79me3 ChIP-seq in SEM cells and super-enhancers (SE). Numbers inside the red circle reflect the number of super-enhancers within each overlap group. **(b)** Number of KEEs and non-KEEs that overlap with super-enhancers or non-super-enhancers. **(c)** Lengths of KEEs (purple) and non-KEEs (gray) in different cell types. Box plots show interquartile range; center line represents the median value. **(d)** Overlap between KEE-genes identified using H3K79me2 and H3K79me3 ChIP-seq in SEM cells and spreading target genes. **(e)** Proportion of MLL-AF4 spreading genes that are also a KEE-gene (purple), non-KEE-gene (light gray), or not annotated with an enhancer (dark gray) (**** = p <0.0001, Fisher's exact test) in SEM cells. **(f-g)** Capture-C signal and level of H3K79me2 at *BCL11A* and *JMJD1C* in SEM and THP1 cells. Blue boxes represent KEE regions, red line shows location of Capture-C probe. **(h)** Capture-C and H3K79me2 ChIP-seq in SEM, RS4;11 and THP1 cells at *ARID1B* and *CDK6.* Blue boxes represent KEE regions. **(i-k)** Capture-C, ChIP-seq and ATAC-seq at *CDK6* in THP1, SEM and RS4;11 cells. Blue boxes represent KEE regions **(l)** Capture-C, ChIP-seq and ATAC-seq at *LMO4* in SEM cells. **(m)** Capture-C, ChIP-seq and ATAC-seq at *BCL11A* in SEM cells. **(n)** Capture-C, ChIP-seq and ATAC-seq at *BCL2* in SEM cells. **(o)** Capture-C and H3K79me2 ChIP-seq in SEM, RS4;11 and THP1 cells at *BCL2* and *LMO4.* Blue boxes represent KEE regions, orange boxes represent non-KEE regions.

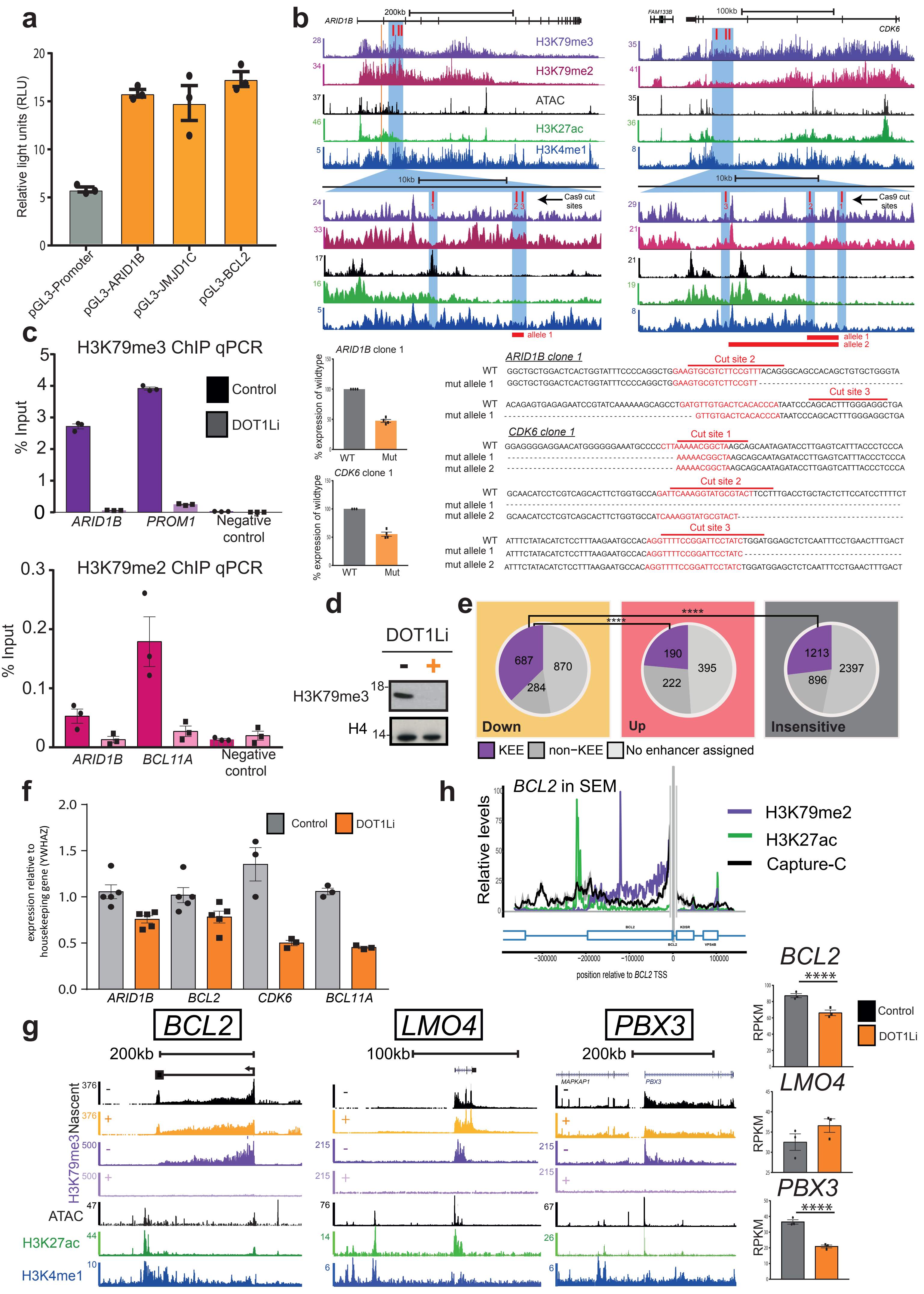

a
Relative light units (RLU)
pGL3-Promoter
pGL3-ARID1B
pGL3-JMJD1C
pGL3-BCL2
b
ARID1B
200kb
H3K79me3
H3K79me2
ATAC
H3K27ac
H3K4me1
10kb
Cas9 cut sites
allele 1
FAM133B
100kb
CDK6
allele 2
ARID1B clone 1
% expression of wildtype
WT
Mut
CDK6 clone 1
ARID1B clone 1
Cut site 2
WT GGCTGCTGGACTCACTGGTATTTCCCCAGGCTGGAAGTGCGTCTTCCGTTTACAGGGCAGCCACAGCTGTGCTGGGTA
mut allele 1 GGCTGCTGGACTCACTGGTATTTCCCCAGGCTGGAAGTGCGTCTTCCGTT
Cut site 3
WT ACAGAGTGAGAGAATCCGTATCAAAAAAGCAGCCTGATGTTGTGACTCACACCCATAATCCCAGCACTTTGGGAGGCTGA
mut allele 1 GTTGTGACTCACACCCATAATCCCAGCACTTTGGGAGGCTGA
CDK6 clone 1
Cut site 1
WT GGAGGGGAGGAACATGGGGGGAAATGCCCCCTTAAAAACGGCTAAGCAGCAATAGATACCTTGAGTCATTTACCCTCCCA
mut allele 1 AAAAACGGCTAAGCAGCAATAGATACCTTGAGTCATTTACCCTCCCA
mut allele 2 AAAAACGGCTAAGCAGCAATAGATACCTTGAGTCATTTACCCTCCCA
Cut site 2
WT GCAACATCCTCGTCAGCACTTCTGGTGCCAGATTCAAAGGTATGCGTACTTCCTTTGACCTGCTACTCTTCCATCCTTTTCT
mut allele 1
mut allele 2 GCAACATCCTCGTCAGCACTTCTGGTGCCATCAAAGGTATGCGTACT
Cut site 3
WT ATTTCTATACATCTCCTTTAAGAATGCCACAGGTTTTCCGGATTCCTATCTGGATGGAGCTCTCAATTTCCTGAACTTTGACT
mut allele 1 ATTTCTATACATCTCCTTTAAGAATGCCACAGGTTTTCCGGATTCCTATC
mut allele 2 ATTTCTATACATCTCCTTTAAGAATGCCACAGGTTTTCCGGATTCCTATCTGGATGGAGCTCTCAATTTCCTGAACTTTGACT
c
H3K79me3 ChIP qPCR
% Input
Control
DOT1Li
ARID1B
PROM1
Negative control
H3K79me2 ChIP qPCR
BCL11A
d
DOT1Li
H3K79me3
H4
e
****
687
870
284
Down
190
395
222
Up
1213
2397
896
Insensitive
KEE
non−KEE
No enhancer assigned
f
expression relative to housekeeping gene (YWHAZ)
Control
DOT1Li
ARID1B
BCL2
CDK6
BCL11A
h
BCL2 in SEM
Relative levels
H3K79me2
H3K27ac
Capture-C
position relative to BCL2 TSS
g
BCL2
200kb
LMO4
100kb
PBX3
200kb
Nascent
H3K79me3
ATAC
H3K27ac
H3K4me1
MAPKAP1
RPKM
Control
DOT1Li

**Supplementary Figure 2.** Loss of H3K79me2/3 leads to a reduction in transcription at KEE-genes **(a)** Luciferase assay for putative enhancer activity. pGL3 plasmid containing promoter alone, or sections of the *ARID1B* KEE, *JMJD1C* KEE or *BCL2* non-KEE were co-transfected with pRL-TK *Renilla* into 293T cells. Firefly luciferase activity was normalised to *Renilla* luciferase. Error bars represent s.e.m of three technical replicates. **(b)** Upper: Locations of gRNA cut sites at *ARID1B* and *CDK6* KEEs (red lines) and *ARID1B* luciferase enhancer (orange line). Lower: Sequence of an *ARID1B* mutant and *CDK6* mutant clone compared to WT, characterised using PCR and Sanger sequencing. Solid red bars below the ChIP-seq traces show the deleted regions. Left: RT-qPCR analysis of *ARID1B* and *CDK6* expression in the indicated clones relative to WT SEM cells, mean of at least three replicates. Error bars represent s.e.m. **(c)** H3K79me3 and H3K79me2 ChIP qPCR in control (dark shade) and DOT1Li (light shade) SEM cells at KEE genes and negative control locus. Error bars represent s.e.m (n=3). **(d)** Western blot analysis of H3K79me3 and H4 in control (-) and DOT1Li (+) SEM cells. Representative of three biological replicates. **(e)** Proportion of DOT1Li downregulated, upregulated or insensitive genes associated with a KEE, non-KEE or no enhancer. **** = $p<0.0001$, Fisher's exact test. **(f)** RT-qPCR of total RNA in control (black) and DOT1Li (orange) SEM cells at *ARID1B*, *BCL2*, *CDK6* and *BCL11A*, normalised to the housekeeping gene *YWHAZ*. Error bars represent s.d (n=3). **(g)** Left: Nascent RNA-seq at *BCL2, LMO4 and PBX3* in control (black, -) and DOT1Li (orange, +) conditions performed in SEM cells, H3K79me3 ChIP-rx in control (purple, -) and DOT1Li (orange, +) conditions, ATAC-seq, H3K27ac and H3K4me1 ChIP-seq. Right: Bar charts showing nascent RNA-seq RPKM values at *BCL2*, *LMO4* and *PBX3* in Control (black) and DOT1Li (orange) conditions. Error bars represent s.d from 3 biological replicates. For p-values see Supplementary Table 2. **(h)** Overlay of Capture-C with H3K79me2 and H3K27ac ChIP-seq at *BCL2* in SEM cells. Gray bars represent location of Capture-C probe, +/-1 kb exclusion zone. Source data are provided as a source data file.

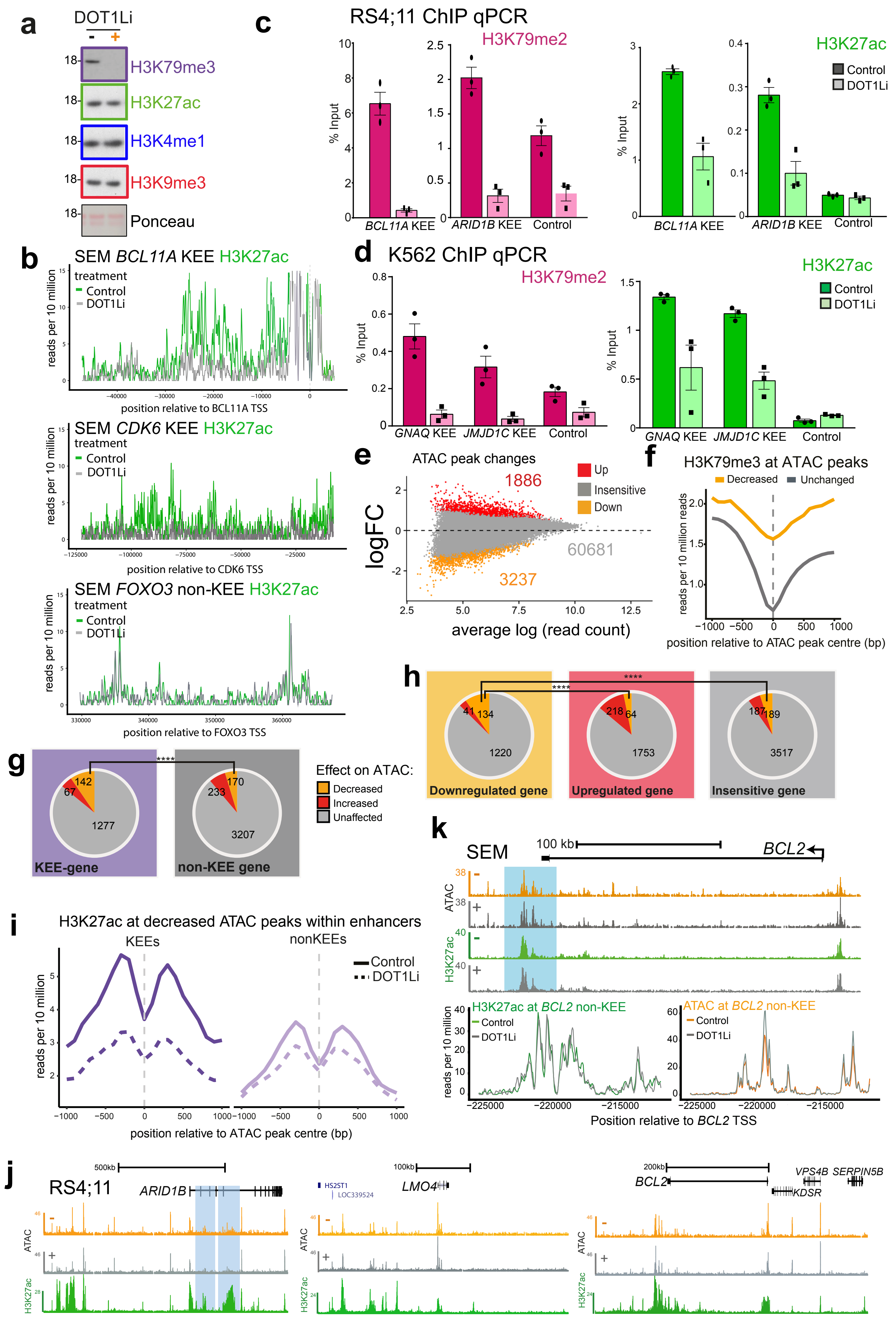
a
DOT1Li
- +
H3K79me3
H3K27ac
H3K4me1
H3K9me3
Ponceau
c
RS4;11 ChIP qPCR
H3K79me2
% Input
BCL11A KEE
ARID1B KEE
Control
H3K27ac
Control
DOT1Li
b
SEM BCL11A KEE H3K27ac
treatment
Control
DOT1Li
reads per 10 million
position relative to BCL11A TSS
SEM CDK6 KEE H3K27ac
position relative to CDK6 TSS
SEM FOXO3 non-KEE H3K27ac
position relative to FOXO3 TSS
d
K562 ChIP qPCR
H3K79me2
H3K27ac
GNAQ KEE
JMJD1C KEE
Control
e
ATAC peak changes
Up
Insensitive
Down
1886
60681
3237
logFC
average log (read count)
f
H3K79me3 at ATAC peaks
Decreased
Unchanged
reads per 10 million reads
position relative to ATAC peak centre (bp)
h
Downregulated gene
Upregulated gene
Insensitive gene
41 134 1220
218 64 1753
187 189 3517
g
KEE-gene
non-KEE gene
142 67 1277
170 233 3207
Effect on ATAC:
Decreased
Increased
Unaffected
k
SEM
100 kb
BCL2
ATAC
H3K27ac
H3K27ac at BCL2 non-KEE
ATAC at BCL2 non-KEE
Position relative to BCL2 TSS
i
H3K27ac at decreased ATAC peaks within enhancers
KEEs
nonKEEs
Control
DOT1Li
reads per 10 million
position relative to ATAC peak centre (bp)
j
RS4;11
500kb
ARID1B
100kb
HS2ST1
LOC339524
LMO4
200kb
BCL2
KDSR
VPS4B
SERPINB5

**Supplementary Figure 3.** Loss of H3K79me2/3 leads to reduction in active histone modifications and chromatin accessibility at KEEs **(a)** Western blot analysis of acid-extracted histones from SEM cells in control (-) and DOT1Li (+) conditions. **(b)** Overlay of H3K27ac ChIP-seq in control (green) and DOT1Li (gray) at *BCL11A*, *CDK6* KEE and *FOXO3* non-KEE region.**(c)** H3K79me2 and H3K27ac ChIP qPCR in RS4;11 cells at KEE-associated genes in control (darker shade) and DOT1Li (lighter shade) conditions. n=3, error bars represent s.e.m. **(d)** H3K79me2 and H3K27ac ChIP qPCR in K562 cells at KEE-associated genes in control (darker shade) and DOT1Li (lighter shade) conditions. n=3, error bars represent s.e.m. **(e)** MA plot representing up (red, 1886), down (orange, 3237) and insensitive (gray, 60681) ATAC peaks following DOT1Li, FDR <0.05, based on five biological replicates. **(f)** Metaplot of H3K79me3 ChIP-seq signal from control SEM cells centered around DOT1Li-decreased (orange) and unchanged (gray) ATAC peaks. **(g)** Proportion of ATAC peaks within KEE genes or non-KEE genes that show increases (red), decreases (orange) or no change (gray) following DOT1Li. **** = p <0.0001, Fisher's exact test. **(h)** Proportion of ATAC peaks within DOT1Li transcriptionally downregulated, upregulated and insensitive genes that show increases (red), decreases (orange) or no change (gray) following DOT1Li. **** = p <0.0001, Fisher's exact test. **(i)** Metaplot of H3K27ac ChIP-seq levels at DOT1Li-decreased ATAC peaks found within KEEs and non-KEEs. H3K27ac levels at these regions were measured from control (solid line) and DOT1Li (dashed line) SEM cells. **(j)** ATAC-seq in control (orange) and DOT1Li (gray) conditions and H3K27ac ChIP-seq (green) in RS4;11 cells at *ARID1B, LMO4* and *BCL2.* Blue boxes at *ARID1B* represent regions where reductions in ATAC correspond to those observed in SEM cells. **(k)** Upper: ATAC-seq and H3K27ac ChIP-seq in control (orange/green) and DOT1Li (gray) SEM cells at *BCL2.* Highlighted blue regions represent region represented in H3K27ac and ATAC-seq overlays below. Lower: Overlay of H3K27ac (left) and ATAC (right) signal at *BCL2* non-KEE in control (green/orange) and DOT1Li (gray) SEM cells. Source data are provided as a source data file.

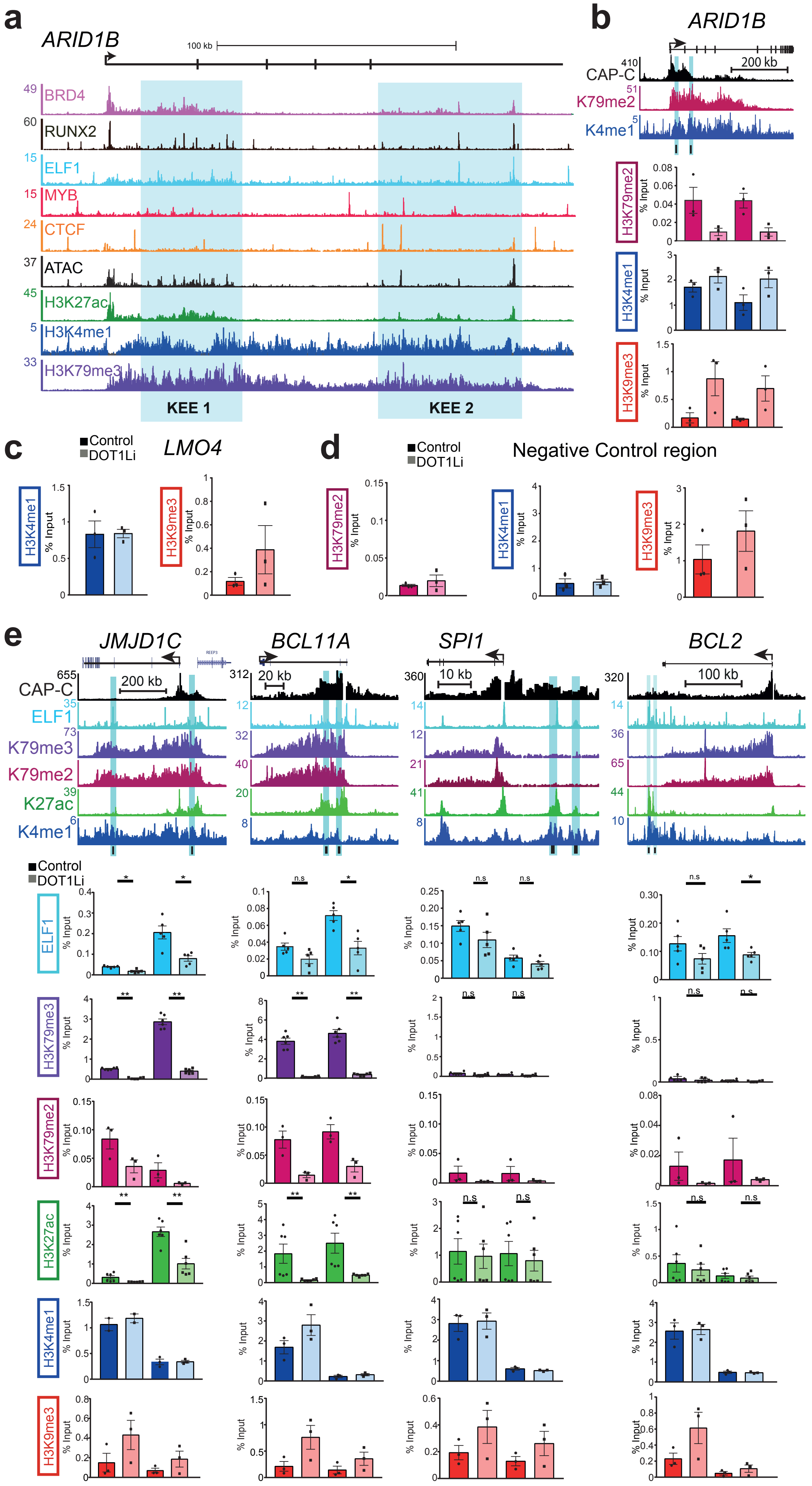
a
ARID1B
100 kb
BRD4
RUNX2
ELF1
MYB
CTCF
ATAC
H3K27ac
H3K4me1
H3K79me3
KEE 1
KEE 2
b
ARID1B
CAP-C
K79me2
K4me1
200 kb
H3K79me2
H3K4me1
H3K9me3
% Input
c
Control
DOT1Li
LMO4
d
Negative Control region
e
JMJD1C
BCL11A
SPI1
BCL2
200 kb
20 kb
10 kb
100 kb
CAP-C
ELF1
K79me3
K79me2
K27ac
K4me1
Control
DOT1Li
H3K79me3
H3K27ac
n.s

**Supplementary Figure 4.** Loss of H3K79me2/3 leads to a reduction in ELF1 binding at KEEs **(a)** ChIP-seq at *ARID1B* demonstrating multiple transcription factor binding events at KEEs in SEM cells. **(b)** H3K79me2, H3K4me1 and H3K9me3 ChIP qPCR at *ARID1B* in SEM cells in control (darker shade) and DOT1Li (lighter shade) conditions. **(c)** H3K4me1 and H3K9me3 ChIP qPCR at *LMO4* in control (darker shade) and DOT1Li (lighter shade) conditions. Error bars represent s.e.m **(d)** H3K79me2, H3K4me1 and H3K9me3 ChIP qPCR at negative control region in control (darker shade) and DOT1Li (lighter shade) conditions. Error bars represent s.e.m **(e)** ChIP qPCR at KEEs and non-KEEs for ELF1, H3K79me3, H3K79me2, H3K27ac, H3K4me1 and H3K9me3 in control (darker shade) and DOT1Li (lighter shade) SEM cells. n=6, error bars represent s.e.m. Statistical test used Mann Whitney U test, * = $p<0.05$, ** = $p<0.01$, n.s. = non-significant. Source data are provided as a source data file.

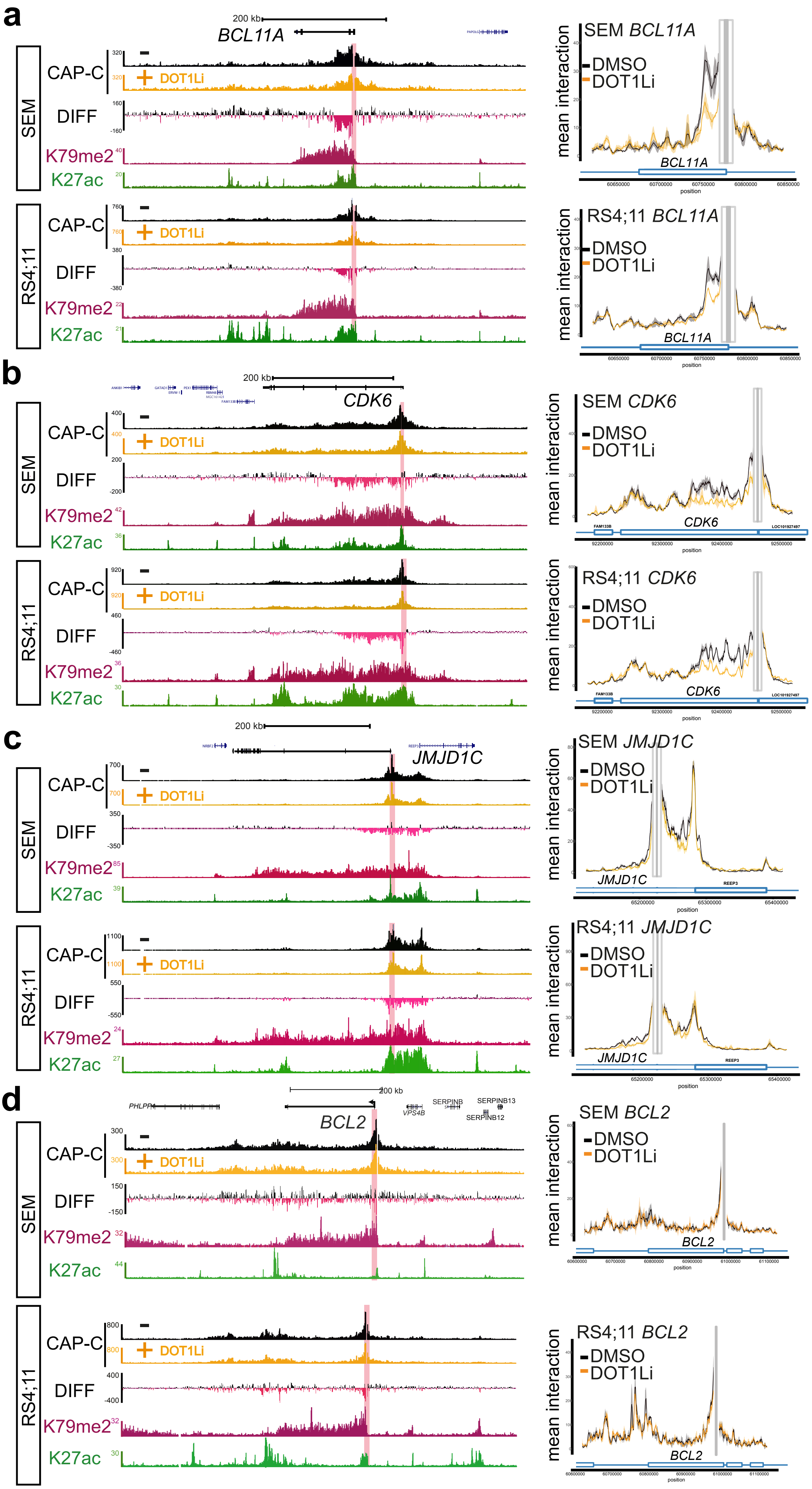
a
b
c
d
200 kb
BCL11A
CDK6
JMJD1C
BCL2
SEM
RS4;11
CAP-C
DOT1Li
DIFF
K79me2
K27ac
mean interaction
position
SEM BCL11A
RS4;11 BCL11A
SEM CDK6
RS4;11 CDK6
SEM JMJD1C
RS4;11 JMJD1C
SEM BCL2
RS4;11 BCL2
DMSO
DOT1Li
FAM133B
LOC101927497
REEP3
PHLPP1
VPS4B
SERPINB
SERPINB13
SERPINB12

**Supplementary Figure 5.** Loss of H3K79me2/3 leads to a reduction in KEE-promoter interactions at *ARID1B* but not non-KEE-promoter interactions at *LMO4* **(a-d)** Capture-C from the promoter of *BCL11A*, *CDK6*, *JMJD1C* and *BCL2* in control (black) and DOT1Li (orange) treatment, in SEM (upper) and RS4;11 (lower) cells, n=3. Left: Differential track demonstrates difference in signal between control and DOT1Li: increase (black), decrease (pink). ChIP-seq tracks for H3K79me2 and H3K27ac. Right: overlay of control (black) and DOT1Li (orange) Capture-C signal across each gene. Gray bars represent location of Capture-C probe, +/-1 kb exclusion zone. Shaded area around Capture-C signal represents 1 s.d.

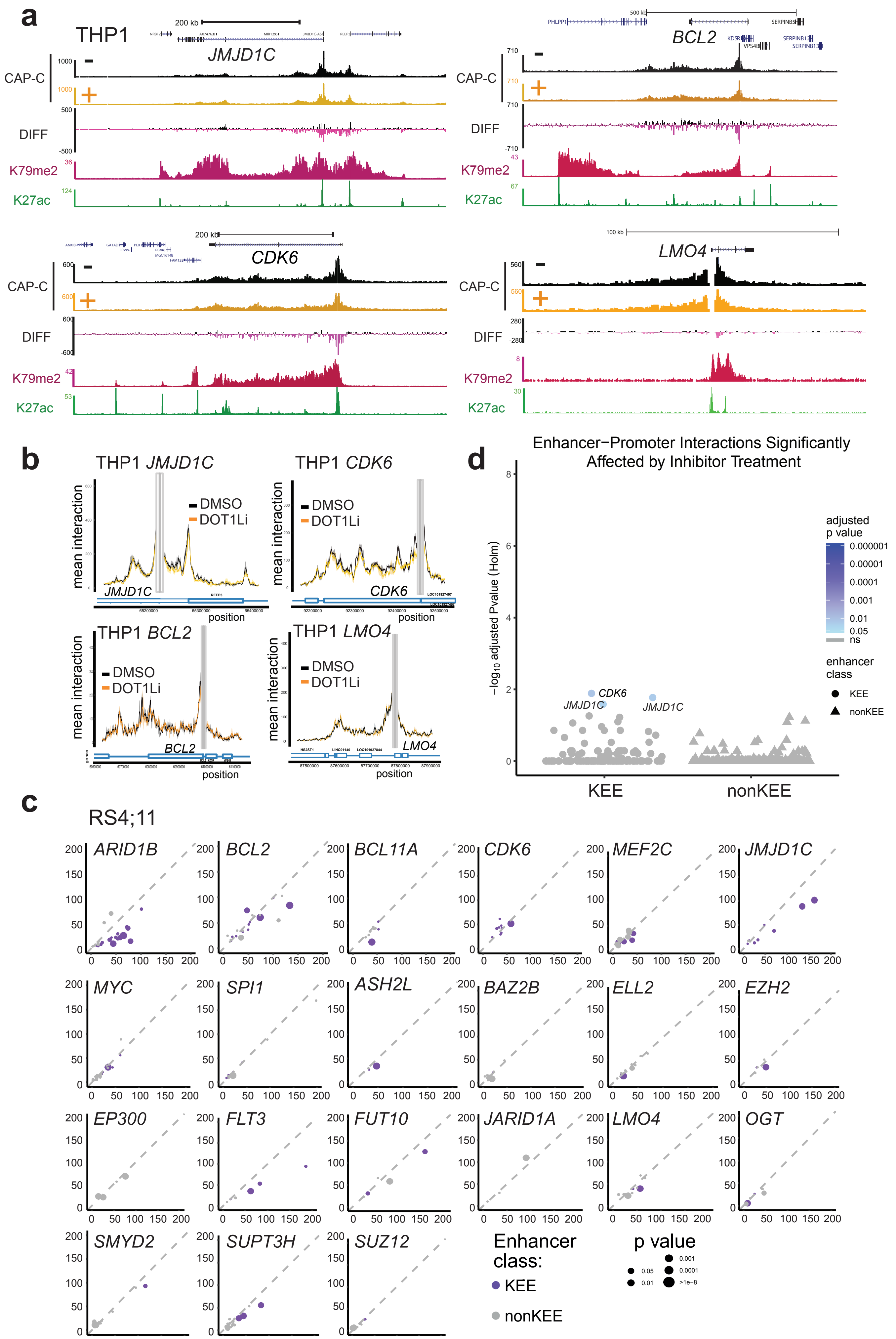

a
THP1
JMJD1C
BCL2
CDK6
LMO4
CAP-C
DIFF
K79me2
K27ac
200 kb
500 kb
100 kb
b
THP1 JMJD1C
THP1 CDK6
THP1 BCL2
THP1 LMO4
mean interaction
position
DMSO
DOT1Li
c
RS4;11
ARID1B
BCL2
BCL11A
CDK6
MEF2C
JMJD1C
MYC
SPI1
ASH2L
BAZ2B
ELL2
EZH2
EP300
FLT3
FUT10
JARID1A
LMO4
OGT
SMYD2
SUPT3H
SUZ12
Enhancer class:
KEE
nonKEE
p value
0.05
0.01
0.001
0.0001
>1e−8
d
Enhancer−Promoter Interactions Significantly Affected by Inhibitor Treatment
−log10 adjusted Pvalue (Holm)
adjusted p value
0.000001
0.00001
0.0001
0.001
0.01
0.05
ns
enhancer class
KEE
nonKEE

**Supplementary Figure 6.** Loss of H3K79me2/3 leads to specific reductions in KEE-promoter interactions at a large number of genes **(a)** Capture-C from the promoter of *JMJD1C, CDK6, BCL2* and *LMO4* in control (black) and DOT1Li (orange) treatment performed in triplicate in THP1 cells. Differential track (increases (black), decreases (pink)) demonstrates difference in signal between control and DOT1Li. ChIP-seq tracks for H3K79me2 and H3K27ac. **(b)** Overlay of control (black) and DOT1Li (orange) Capture-C signal (average of three biological replicates) across *JMJD1C*, *CDK6*, *BCL2* and *LMO4* in THP1 cells. Gray bars represent location of Capture-C probe, +/-1 kb exclusion zone. **(c)** Enhancer-promoter Capture-C interaction frequencies in Control (x-axis) and DOT1Li (y-axis) RS4;11 cells. Each point represents the interaction of a KEE (purple) or non-KEE (gray) with the indicated gene promoter. Size of dot corresponds to significance of change in interaction, using a Wilcoxon rank test. See Supplementary Table 6 for list of p values. **(d)** Statistical analysis of the significance of the change in enhancer-promoter Capture-C interactions following DOT1Li in THP1 cells. Each point represents the interaction of a KEE (circle) or non-KEE (triangle) with a gene promoter. Significantly affected interactions are labelled with the relevant gene promoter. Holm-Bonferroni adjusted p-values were calculated following a Wilcoxon rank test (n=3).

| Primer name | Sequence |
|---|---|
| ARID1B KEE1 F primer | TTTCCAGTTCCGTCCAAGTATT |
| ARID1B KEE1 R primer | CTTTCTCCCAGCTTCCTGTTAG |
| ARID1B KEE2 F primer | CTAACAGGAAGCTGGGAGAAAG |
| ARID1B KEE2 R primer | GCTTCTTGCTTAAGGCGTTTC |
| BCL11A KEE1 F primer | GCAAGCCAGGAAAGATGATAGA |
| BCL11A KEE1 R primer | AGAGACGCACAGCTCAATTT |
| BCL11A KEE2 F primer | ACACCCAGTGCCCAGAATTG |
| BCL11A KEE2 R primer | CGCGGGTCCTGAGATTCATT |
| JMJD1C KEE1 F primer | CTTCCAGCACGAGTCACATTA |
| JMJD1C KEE1 R primer | CTCGCCACTAGTCTGTCTTTG |
| JMJD1C KEE2 F primer | CAAGGTACGTCTGCGAGAG |
| JMJD1C KEE2 R primer | CGTGTCACACAGGGACAG |
| SPI-1 non-KEE1 F primer | TAACAGCTTCCGCCAAGAG |
| SPI1-non-KEE1 R primer | CGCAGAGTTGAGACCAGAG |
| SPI-1 non-KEE2 F primer | GCCAAACACTAGGTCAGAGAAA |
| SPI1 non-KEE2 R primer | CCTACTTTGGCCACCCTTAAA |
| BCL2 non-KEE1 F primer | CTTCACGCAGCATCCATTTG |
| BCL2 non-KEE1 R primer | CCAATTCAGCATCTAGTTCTCCT |
| BCL2 non-KEE2 F primer | CCGGCATCTGAGCTAATTACA |
| BCL2 non-KEE2 R primer | TCTGCCCAGTACCTCTTATCT |
| PROM1 intron 1 F primer | GGTGACACAATTCAACCCATAAC |
| PROM1 intron 1 R primer | CATGGGTCTTTAGTTGGTGTAGTA |
| GNAQ F primer | GTCCATCATGGCGTGCT |
| GNAQ R primer | CGGACGGTACTCACCGA |
| Negative control F primer | GGCTCCTGTAACCAACCACTACC |
| Negative control R primer | CCTCTGGGCTGGCTTCATTC |
| BCL2en luciferase assay F primer | ATGCATCGATGTCTTCACAGCCGTCATCCT |
| BCL2en luciferase assay R primer | ATGCGCGGCCGCGCCAATGACATACGACAGC |
| ARID1B KEE luciferase assay F | GGTGTCAGAAGTTGGGGCTA |
| ARID1B KEE luciferase assay R | GCTTGCTCCTAGGAAATACACC |
| JMJD1C KEE luciferase assay | AAATCGATAAGGATCCAGCTTGAAAATATCTTTCCATAATTTCTACTCTATTA CAC |
| JMJD1C KEE luciferase assay | AAGGGCATCGGTCGATTGCTATAGGGATTTGGGTACTGATGC |
| YWHAZ Taqman probe mix | Hs03044281_g1 |
| ARID1B Taqman probe mix | Hs00368175_m1 |
| CDK6 Taqman probe mix | Hs01026371_m1 |
| BCL2 Taqman probe mix | Hs00608023_m1 |
| BCL11A Taqman pribe mix | Hs01093197_m1 |

**Supplementary Table 1**. List of primers used.

| Experiment | Cell line | Antibody | Accession Number |
|---|---|---|---|
| ChIP seq | SEM | H3K27ac | GSE74812 |
| ChIP seq | SEM | H3K79me2 | GSE74812 |
| ChIP seq | SEM | H3K79me3 | GSE74812 |
| ChIP seq | SEM | H3K4me1 | GSE74812 |
| ChIP seq | SEM | H3K4me3 | GSE74812 |
| ChIP seq | SEM | BRD4 | GSE83671 |
| ChIP seq | RS4;11 | H3K79me2 | GSE38403 |
| ChIP seq | RS4;11 | H3K27ac | GSE71616 |
| ChIP seq | RS4;11 | H3K4me1 | GSE71616 |
| ChIP seq | RS4;11 | H3K4me3 | GSE71616 |
| ChIP seq | ML-2 | H3K79me2 | GSE83671 |
| ChIP seq | K562 | H3K27ac | GSE29611 |
| ChIP seq | K562 | H3K79me2 | GSE29611 |
| ChIP seq | K562 | H3K4me1 | GSE31755 |
| ChIP seq | K562 | H3K4me3 | GSE96303 |
| ChIP seq | GM12878 | H3K27ac | GSE29611 |
| ChIP seq | GM12878 | H3K79me2 | GSE29611 |
| ChIP seq | GM12878 | H3K4me1 | GSE29611 |
| ChIP seq | GM12878 | H3K4me3 | GSE29611 |
| ChIP seq | HeLa-S3 | H3K27ac | GSE29611 |
| ChIP seq | HeLa-S3 | H3K79me2 | GSE29611 |
| ChIP seq | HeLa-S3 | H3K4me1 | GSE29611 |
| ChIP seq | HeLa-S3 | H3K4me3 | GSE29611 |
| ChIP seq | H1-hESC | H3K27ac | GSE16256 |
| ChIP seq | H1-hESC | H3K79me2 | GSE16256 |
| ChIP seq | H1-hESC | H3K4me1 | GSE16256 |
| ChIP seq | H1-hESC | H3K4me3 | GSE16256 |
| Nascent RNA seq | SEM | | GSE83671 |
| PolyA RNA seq | K562 | | GSE88351 |
| PolyA RNA seq | GM12878 | | GSE88583 |
| PolyA RNA seq | HeLa-S3 | | GSE33480 |
| PolyA RNA seq | H1-hESC | | GSE16256 |

**Supplementary Table 2**. GEO accession numbers: datasets from previous publications.
