# Supplementary material for: DOT1L inhibition reveals a distinct subset of enhancers dependent on H3K79 methylation
Source: Nat Commun. 2019 Jun 26;10:2803. doi: 10.1038/s41467-019-10844-3 (PMC6594956; doi:10.1038/s41467-019-10844-3)
Supplement: Supplementary file 3 — Reporting Summary [file 41467_2019_10844_MOESM3_ESM.pdf]

nature research

Corresponding author(s): Thomas A. Milne

Last updated by author(s): Mar 28, 2019

Reporting Summary

Nature Research wishes to improve the reproducibility of the work that we publish. This form provides structure for consistency and transparency in reporting. For further information on Nature Research policies, see Authors & Referees and the Editorial Policy Checklist.

Statistics

For all statistical analyses, confirm that the following items are present in the figure legend, table legend, main text, or Methods section.

n/a	Confirmed	
☐	☒	The exact sample size (*n*) for each experimental group/condition, given as a discrete number and unit of measurement
☐	☒	A statement on whether measurements were taken from distinct samples or whether the same sample was measured repeatedly
☐	☒	The statistical test(s) used AND whether they are one- or two-sided<br>*Only common tests should be described solely by name; describe more complex techniques in the Methods section.*
☐	☒	A description of all covariates tested
☐	☒	A description of any assumptions or corrections, such as tests of normality and adjustment for multiple comparisons
☐	☒	A full description of the statistical parameters including central tendency (e.g. means) or other basic estimates (e.g. regression coefficient) AND variation (e.g. standard deviation) or associated estimates of uncertainty (e.g. confidence intervals)
☐	☒	For null hypothesis testing, the test statistic (e.g. *F*, *t*, *r*) with confidence intervals, effect sizes, degrees of freedom and *P* value noted<br>*Give P values as exact values whenever suitable.*
☒	☐	For Bayesian analysis, information on the choice of priors and Markov chain Monte Carlo settings
☒	☐	For hierarchical and complex designs, identification of the appropriate level for tests and full reporting of outcomes
☒	☐	Estimates of effect sizes (e.g. Cohen's *d*, Pearson's *r*), indicating how they were calculated

Our web collection on statistics for biologists contains articles on many of the points above.

Software and code

Policy information about availability of computer code

Data collection	High throughput sequencing data was collected via Basespace (Illumina). For qPCR Quantstudio (Thermofisher Scientific) was used.
Data analysis	For ChIP-seq and ATAC-seq, quality control of FASTQ reads, genome alignment, PCR duplicate filtering, blacklisted region filtering and UCSC data hub generation was performed using an in-house pipeline: https://github.com/Hughes-Genome-Group/NGseqBasic/releases. Directories of sequence tags (reads) were generated from the sam files using the Homer tool makeTagDirectory. The makeBigWig.pl command was used to generate bigwig files for visualisation in UCSC, normalising tag counts to tags per 10 million. Peaks were called using the Homer tool findPeaks, with the input track provided for background correction, using the -style histone or -style factor options to call peaks in histone modification or transcription factor/ATAC datasets, respectively. Statistical analysis of differences between ATAC peaks was conducted with Diffbind, using the edgeR package.<br>For RNA-seq analysis, following QC analysis with the fastQC package (http://www.bioinformatics.babraham.ac.uk/projects/fastqc), reads were aligned using STAR. Reads that were identified as PCR duplicates using Samtools were discarded. Gene expression levels were quantified as read counts using the featureCounts function from the Subread package with default parameters. The read counts were used for the identification of global differential gene expression between specified populations using the edgeR package.<br>Capture-C analysis was performed using an in-house pipeline: github.com/Hughes-Genome-Group/CCseqBasicF/releases

For manuscripts utilizing custom algorithms or software that are central to the research but not yet described in published literature, software must be made available to editors/reviewers. We strongly encourage code deposition in a community repository (e.g. GitHub). See the Nature Research guidelines for submitting code & software for further information.

Data

Policy information about availability of data

All manuscripts must include a data availability statement. This statement should provide the following information, where applicable:

- Accession codes, unique identifiers, or web links for publicly available datasets
- A list of figures that have associated raw data
- A description of any restrictions on data availability

All high throughput data have been deposited in the Gene Expression Omnibus (GEO) under the accession number GSE117865.
Listed below are the datasets associated with each figure:
Figure 1: THP1 ChIP-seq (H3K4me1, H3K4me3, H3K27ac, H3K79me2 and associated inputs), THP1 nascent-RNA seq, ML-2 RNA-seq, SEM Capture-C (DMSO), THP1 Capture-C (DMSO)
Figure 2: SEM H3K79me3 ChIP-rx (DMSO and 2uM EPZ-5676)
Figure 3: SEM H3K27ac ChIP-seq (DMSO and 2uM EPZ-5676), SEM ATAC-seq (DMSO and 2uM EPZ-5676)
Figure 4: SEM ELF1 ChIP-seq, SEM Capture-C (DMSO)
Figure 5: SEM Capture-C (DMSO and 2uM EPZ-5676), RS4;11 Capture-C (DMSO and 2uM EPZ-5676)
Figure 6: SEM Capture-C (DMSO and 2uM EPZ-5676), RS4;11 Capture-C (DMSO and 2uM EPZ-5676)
Supplementary figure 1: SEM Capture-C (DMSO), THP1 Capture-C (DMSO), RS4;11 Capture-C (DMSO), THP1 ChIP-seq (H3K4me1, H3K27ac, H3K79me2)
Supplementary figure 2: SEM ATAC (DMSO), SEM H3K79me3 ChIP-rx (DMSO and 2uM EPZ-5676)
Supplementary figure 3: SEM H3K79me3 ChIP-rx (DMSO and 2uM EPZ-5676), SEM H3K27ac ChIP-seq (DMSO and 2uM EPZ-5676), SEM ATAC-seq (DMSO and 2uM EPZ-5676), RS4;11 ATAC-seq (DMSO and 2uM EPZ-5676)
Supplementary figure 4: SEM BRD4, RUNX2, ELF1, MYB, CTCF ChIP-seq, SEM ATAC (DMSO), SEM Capture-C (DMSO)
Supplementary figure 5: SEM Capture-C (DMSO and 2uM EPZ-5676), RS4;11 Capture-C (DMSO and 2uM EPZ-5676)
Supplementary figure 6: RS4;11 Capture-C (DMSO and 2uM EPZ-5676), THP1 Capture-C (DMSO and 2uM EPZ-5676), THP1 ChIP-seq (H3K27ac, H3K79me2)

No restrictions on data availability.

Field-specific reporting

Please select the one below that is the best fit for your research. If you are not sure, read the appropriate sections before making your selection.

☒ Life sciences ☐ Behavioural & social sciences ☐ Ecological, evolutionary & environmental sciences

For a reference copy of the document with all sections, see nature.com/documents/nr-reporting-summary-flat.pdf

Life sciences study design

All studies must disclose on these points even when the disclosure is negative.

Sample size

Statistical methods were not used to assign sample size

Data exclusions

No data was excluded from this analysis

Replication

Listed below are the number of replicates and statistical tests used for each figure (see also figure legends and Supplementary Table 7):

Figure 1a. H3K79me2 and H3K79me3 ChIP-seq in SEM cells. Based on one biological replicate.
Figure 1b-c. ChromHMM data based upon novel or published ChIP-seq databases from one biological replicate in each cell type.
Figure 1d. Gene expression data based upon three biological replicates for SEM and THP1, one biological replicate for all other cell types. Statistical analysis using Mann Whitney U test.
Figure 1e. Capture-C is average of three biological replicates. ATAC-seq is representative of five biological replicates. ChIP-seq from one biological replicate.
Figure 1f-g. Capture-C tracks shown are averages of three biological replicates performed in SEM and RS4;11 cells.
Figure 2a. CRISPR-edited SEM clones for enhancer deletion analysis based upon at least nine independently-isolated clones per enhancer targeted (each point represents one clone).
Figure 2b, g. H3K79me3 ChIP-rx (Control and DOT1Li) from one biological replicate.
Figure 2c-g. Nascent RNA-seq analysis (Control and DOT1Li) based on three biological replicates with differentially expressed genes identified using an FDR of <0.05. Statistically significant analysis using Fisher's exact test.
Figure 3a, c-d. H3K27ac ChIP-seq analysis (Control and DOT1Li) from one biological replicate.
Figure 3b-d. ATAC-seq analysis based on five biological replicates (tracks show one representative sample). Statistical analysis carried out using Fisher's exact test.
Figure 4b. Western blot analysis is representative of three biological replicates.
Figure 4c. All ChIP qPCR data performed in at least three biological replicates. Error bars represent standard error of the mean. Statistical analysis performed using a Mann Whitney-U test.
Figure 5a-c. Capture-C tracks are an average of three biological replicates. ATAC-seq is representative of five biological replicates in SEM cells and one biological replicate in RS4;11 cells.
Figure 6a-c. Capture-C performed in triplicate. Statistical analysis performed on three biological replicates using Wilcoxon rank test in SEM and RS4;11 cells.
Supplementary figure 1. All ChIP-seq representative of one biological replicate. Capture-C mean of three biological replicates. ATAC-seq representative of five biological replicates in SEM cells and one biological replicate in RS4;11.
Supplementary figure 2a. Luciferase assay representative of three technical replicates. Error bars show standard deviation.

Supplementary figure 2c. H3K79me2 and H3K79me3 ChIP qPCR was performed in triplicate (control and DOT1Li conditions). Error bars show standard error.
Supplementary figure 2d. Western blot analysis representative of three biological replicates.
Supplementary figure 2e. Analysis using nascent RNA-seq (control and DOT1Li conditions) based upon three biological replicates. Statistical analysis using a Fisher's exact test with p value <0.0001.
Supplementary figure 2f. RT-qPCR was performed in triplicate (control and DOT1Li conditions). Error bars represent standard deviation.
Supplementary figure 2g. Nascent RNA-seq tracks (control and DOT1Li) representative of three biological replicates. H3K79me3 ChIP-rx and all other ChIP-seq tracks (control and DOT1Li conditions) from one biological replicate. ATAC-seq tracks representative of five biological replicates. Bar charts show RPKM values from nascent RNA-seq data, mean of three biological replicates. Error bars show standard deviation.
Supplementary figure 2h. Capture-C performed in triplicate. ChIP seq from one biological replicate.
Supplementary figure 3a. Western blot analysis representative of three biological replicates (control and DOT1Li conditions).
Supplementary figure 3b. H3K27ac ChIP seq from one biological replicate.
Supplementary figure 3c-d. H3K79me2 and H3K27ac ChIP qPCR performed in triplicate (control and DOT1Li conditions). Error bars show standard error.
Supplementary figure 3e. ATAC-seq analysis (Control and DOT1Li) based on five biological replicates with differential peaks identified using an FDR of <0.05. Statistically significant analysis using Fisher's exact test.
Supplementary figure 3f. H3K79me3 ChIP-seq from one biological replicate.
Supplementary figure 3g-h. ATAC-seq performed in five biological replicates. Nascent RNA-seq performed in three biological replicates. Statistical analysis performed using a Fisher's exact test.
Supplementary figure 3i. H3K27ac ChIP-seq from one biological replicate. ATAC seq performed in five biological replicates.
Supplementary figure 3j. ATAC-seq tracks (control and DOT1Li) from one biological replicate in RS4;11 cells. H3K27ac ChIP-seq track is from one biological replicate in RS4;11 cells.
Supplementary figure 3k. ATAC-seq tracks representative of five biological replicates. H3K27ac ChIP-seq from one biological replicate.
Supplementary figure 4a. ChIP-seq tracks are from one biological replicate.
Supplementary figure 4b-e. All ChIP qPCR (control and DOT1Li) was performed in at least three biological replicates. Statistical analysis was performed using a Mann Whitney U test.
Supplementary figure 5. All Capture-C was performed in three biological replicates (control and DOT1Li conditions). For histograms, shaded ribbon around Capture-C signal represents one standard deviation.
Supplementary figure 6a, c. Capture-C performed in triplicate. Statistical analysis performed on three biological replicates using Wilcoxon rank test in RS4;11 and THP1 cells.
Supplementary figure 6b. Capture-C tracks show the mean of three biological replicates. ChIP-seq tracks are from one biological replicate.
Supplementary figure 6d. Histograms of Capture-C represent mean signal over three biological replicates. Shaded regions around Capture-C signal represents one standard deviation.

Randomization: Randomization was not used in this study due to the use of cell lines

Blinding: Investigators were not blinded

Reporting for specific materials, systems and methods

We require information from authors about some types of materials, experimental systems and methods used in many studies. Here, indicate whether each material, system or method listed is relevant to your study. If you are not sure if a list item applies to your research, read the appropriate section before selecting a response.

Materials & experimental systems

n/a	Involved in the study	
☐	☒	Antibodies
☐	☒	Eukaryotic cell lines
☒	☐	Palaeontology
☒	☐	Animals and other organisms
☒	☐	Human research participants
☒	☐	Clinical data

Methods

n/a	Involved in the study	
☐	☒	ChIP-seq
☒	☐	Flow cytometry
☒	☐	MRI-based neuroimaging

Antibodies

Antibodies used:
Anti-H3K79me3: Diagenode cat no. C15410068 lot no. A246-0040. Validated for Western blotting and ChIP-seq in human cell lines stated on the manufacturer's datasheet
Anti-H3K79me2 (ChIP-seq): Millipore cat no. 04/835 Validated for western blotting and ChIP-seq in human cell lines on manufacturer's website.
Anti-H3K79me2 (ChIP-qPCR and western blotting): Abcam cat no. ab3594.
Anti-H3K27ac: Diagenode cat no. C15410196 lot no. A1723-0041D. Validated for western blotting and ChIP-seq in human cell lines on manufacturer's website.
Anti-H3K4me1 (ChIP-seq): Diagenode cat no. pAB-194-050. Validated for western blotting and ChIP-seq in human cell lines on manufacturer's website.
Anti-H3K4me1 (ChIP-qPCR and western blotting): Abcam cat no. ab8895. Validated for western blotting and ChIP in human cell lines on manufacturer's website.
Anti-H3K4me3: Diagenode cat no. pAB-003-050. Validated for western blotting and ChIP-seq in human cell lines on manufacturer's website.
Anti-ELF1: Bethyl cat no. A301-443A. lot no. 1. Validated for western blotting in human cell lines on manufacturer's website.

Anti-RUNX2: Cell Signalling cat no. 8486 lot no. 1. Validated for western blotting and ChIP in human cell lines on manufacturer's website.
Anti-MYB: Abcam cat no. ab177510. Validated for western blotting and IP in human cell lines on manufacturer's website.
Anti-CTCF. Millipore cat no. 07-729. Validated for western blotting and ChIP-seq in human cell lines on manufacturer's website.

Validation

See above box. All antibodies used were verified for specificity as described on the manufacturers websites/datasheets.

Eukaryotic cell lines

Policy information about cell lines

Cell line source(s)

SEM and ML-2 cells were purchased from DSMZ (www.cell-lines.de). RS4;11, THP1, K562 and 293T cells were purchased from ATCC.

Authentication

Cells were validated by DSMZ and ATCC.

Mycoplasma contamination

All cell lines were confirmed to be mycoplasma free.

Commonly misidentified lines
(See ICLAC register)

None

ChIP-seq

Data deposition

☒ Confirm that both raw and final processed data have been deposited in a public database such as GEO.

☒ Confirm that you have deposited or provided access to graph files (e.g. BED files) for the called peaks.

Data access links
May remain private before publication.

To review GEO accession GSE117865:
Go to https://www.ncbi.nlm.nih.gov/geo/query/acc.cgi?acc=GSE117865

Files in database submission

INPUT_CTCF_R1.fastq.gz
INPUT_CTCF_R2.fastq.gz
CTCF_R1.fastq.gz
CTCF_R2.fastq.gz
INPUT_RUNX2.fastq.gz
RUNX2.fastq.gz
INPUT_ELF1_R1.fastq.gz
INPUT_ELF1_R2.fastq.gz
ELF1_R1.fastq.gz
ELF1_R2.fastq.gz
Input_MYB_R1.fastq.gz
Input_MYB_R2.fastq.gz
MYB_R1.fastq.gz
MYB_R2.fastq.gz
Input_H3K27ac_0uM_R1.fastq.gz
Input_H3K27ac_0uM_R2.fastq.gz
H3K27ac_0uM_R1.fastq.gz
H3K27ac_0uM_R2.fastq.gz
Input_H3K27ac_2uM_R1.fastq.gz
Input_H3K27ac_2uM_R2.fastq.gz
H3K27ac_2uM_R1.fastq.gz
H3K27ac_2uM_R2.fastq.gz
Input_H3K79me3_0uM_rx_R1.fastq.gz
Input_H3K79me3_0uM_rx_R2.fastq.gz
H3K79me3_0uM_rx_R1.fastq.gz
H3K79me3_0uM_rx_R2.fastq.gz
Input_H3K79me3_2uM_rx_R1.fastq.gz
Input_H3K79me3_2uM_rx_R1.fastq.gz
H3K79me3_2uM_rx_R1.fastq.gz
H3K79me3_0uM_rx_R2.fastq.gz
THP1_H3K27ac_R1.fastq.gz
THP1_H3K27ac_R2.fastq.gz
THP1_H3K4me3_R1.fastq.gz
THP1_H3K4me3_R2.fastq.gz
THP1_Input_H3K27acK4me3_R1.fastq.gz
THP1_Input_H3K27acK4me3_R2.fastq.gz
THP1_H3K4me1_R1.fastq.gz
THP1_H3K4me1_R2.fastq.gz
THP1_Input_H3K4me1_R1.fastq.gz
THP1_Input_H3K4me1_R2.fastq.gz

THP1_H3K79me2_R1.fastq.gz
THP1_H3K79me2_R2.fastq.gz
THP1_Input_H3K79me2_R1.fastq.gz
THP1_Input_H3K79me2_R2.fastq.gz
ATAC_DMSO_1_R1.fastq.gz
ATAC_DMSO_1_R2.fastq.gz
ATAC_DMSO_2_R1.fastq.gz
ATAC_DMSO_2_R2.fastq.gz
ATAC_DMSO_3_R1.fastq.gz
ATAC_DMSO_3_R2.fastq.gz
ATAC_DMSO_4_R1.fastq.gz
ATAC_DMSO_4_R2.fastq.gz
ATAC_DMSO_5_R1.fastq.gz
ATAC_DMSO_5_R2.fastq.gz
ATAC_EPZ_1_R1.fastq.gz
ATAC_EPZ_1_R2.fastq.gz
ATAC_EPZ_2_R1.fastq.gz
ATAC_EPZ_2_R2.fastq.gz
ATAC_EPZ_3_R1.fastq.gz
ATAC_EPZ_3_R2.fastq.gz
ATAC_EPZ_4_R1.fastq.gz
ATAC_EPZ_4_R2.fastq.gz
ATAC_EPZ_5_R1.fastq.gz
ATAC_EPZ_5_R2.fastq.gz
RS411_ATAC_DMSO_R1.fastq.gz
RS411_ATAC_DMSO_R2.fastq.gz
RS411_ATAC_EPZ_R1.fastq.gz
RS411_ATAC_EPZ_R2.fastq.gz
THP1_RNA_1_R1.fastq.gz
THP1_RNA_2_R1.fastq.gz
THP1_RNA_3_R1.fastq.gz
ML2_RNA_R1.fastq.gz
SEM_CapC_0uM_1_R1.fastq.gz
SEM_CapC_0uM_2_R1.fastq.gz
SEM_CapC_0uM_3_R1.fastq.gz
SEM_CapC_2uM_1_R1.fastq.gz
SEM_CapC_2uM_2_R1.fastq.gz
SEM_CapC_2uM_3_R1.fastq.gz
RS411_CapC_0uM_1_R1.fastq.gz
RS411_CapC_0uM_2_R1.fastq.gz
RS411_CapC_0uM_3_R1.fastq.gz
RS411_CapC_2uM_1_R1.fastq.gz
RS411_CapC_2uM_2_R1.fastq.gz
RS411_CapC_2uM_3_R1.fastq.gz
THP1_CapC_0uM_1_R1.fastq.gz
THP1_CapC_0uM_2_R1.fastq.gz
THP1_CapC_0uM_3_R1.fastq.gz
THP1_CapC_5uM_1_R1.fastq.gz
THP1_CapC_5uM_2_R1.fastq.gz
THP1_CapC_5uM_3_R1.fastq.gz

Genome browser session
(e.g. UCSC)

ChromHMM data:
http://genome.ucsc.edu/s/ncrump/Godfrey%20ChromHMM
SEM data (Capture-C set 1):
http://genome.ucsc.edu/s/ncrump/Godfrey%20SEM%20CapC%20set1
SEM data (Capture-C set 2):
http://genome.ucsc.edu/s/ncrump/Godfrey%20SEM%20CapC%20set2
RS4;11 data:
http://genome.ucsc.edu/s/ncrump/Godfrey%20RS411%20CapC
THP1 data:
http://genome.ucsc.edu/s/ncrump/Godfrey%20THP1%20CapC

NB For Capture-C hubs, ConT_mean = DMSO, ConD_mean = DOT1Li, DIF_conD_minus_conT_mean = difference

Methodology

Replicates

One replicate for all ChIP seq datasets. Three biological replicates for all Capture-C (Control and DOT1Li). Five biological replicates for ATAC seq in SEM cells (Control and DOT1Li), one replicate for RS4;11 ATAC seq (Control and DOT1Li).

Sequencing depth

CTCF (SEM): total reads: 48865388 uniquely mapped reads: 38651978 read length: 40 bp, Paired end
CTCF Input (SEM): total reads: 68925276 uniquely mapped reads: 54354102 read length: 40 bp, Paired end
MYB (SEM): total reads: 51566498 uniquely mapped reads: 39895502 read length: 40 bp, Paired end
MYB Input (SEM): total reads: 67830048 uniquely mapped reads: 54826330 read length: 40 bp, Paired end
ELF1 (SEM): total reads: 116158988 uniquely mapped reads: 89892848 read length: 40 bp, Paired end
ELF1 Input (SEM): total reads: 28067866 uniquely mapped reads: 10527434 read length: 40 bp, Paired end

RUNX2 (SEM): total reads: 51415940 uniquely mapped reads: 40421256 read length: 50 bp, Single end
RUNX2 Input (SEM): total reads: 120081552 uniquely mapped reads: 101334464 read length: 50 bp, Single end
H3K27ac (DMSO) (SEM): total reads: 33337576 uniquely mapped reads: 25433120 read length: 40 bp, Paired end
H3K27ac Input (DMSO) (SEM): total reads: 35934204 uniquely mapped reads: 27421698 read length: 40 bp, Paired end
H3K27ac (EPZ) (SEM): total reads: 32457508 uniquely mapped reads: 23093524 read length: 40 bp, Paired end
H3K27ac Input (EPZ) (SEM): total reads: 26531312 uniquely mapped reads: 19419704 read length: 40 bp, Paired end
H3K79me3 (DMSO) (SEM ChIP-rx): total reads: 100243036 uniquely mapped reads: 63296990 read length: 40 bp, Paired end
H3K79me3 Input (DMSO) (SEM ChIP-rx): total reads: 141706504 uniquely mapped reads: 106004614 read length: 40 bp, Paired end
H3K79me3 (EPZ) (SEM ChIP-rx): total reads: 74763896 uniquely mapped reads: 14507312 read length: 40 bp, Paired end
H3K79me3 Input (EPZ) (SEM ChIP-rx): total reads: 119282564 uniquely mapped reads: 85250254 read length: 40 bp, Paired end
H3K79me3 (DMSO) (ChIP-rx S2 spike-in) total reads: 100243036 uniquely mapped reads: 16848930 read length: 40 bp, Paired end
H3K79me3 Input (DMSO) (ChIP-rx S2 spike-in) total reads: 141706504 uniquely mapped reads: 7072834 read length: 40 bp, Paired end
H3K79me3 (EPZ) (ChIP-rx S2 spike-in) total reads: 74763896 uniquely mapped reads: 44070662 read length: 40 bp, Paired end
H3K79me3 Input (EPZ) (ChIP-rx S2 spike-in) total reads: 119282564 uniquely mapped reads: 8798818 read length: 40 bp, Paired end
H3K27ac (THP1): total reads: 67318900 uniquely mapped reads: 56376638 read length: 40 bp, Paired end
H3K4me3 (THP1): total reads: 63065900 uniquely mapped reads: 54514184 read length: 40 bp, Paired end
H3K27ac, K4me3 Input (THP1): total reads: 70696836 uniquely mapped reads: 56597714 read length: 40 bp, Paired end
H3K4me1 (THP1): total reads: 47796288 uniquely mapped reads: 38816332 read length: 40 bp, Paired end
H3K4me1 Input (THP1): total reads: 45596096 uniquely mapped reads: 36901714 read length: 40 bp, Paired end
H3K79me2 (THP1): total reads: 68564950 uniquely mapped reads: 54696576 read length: 40 bp, Paired end
H3K79me2 Input (THP1): total reads: 31661654 uniquely mapped reads: 24390350 read length: 40 bp, Paired end

Antibodies

Anti-H3K79me3: Diagenode cat no. C15410068 lot no. A246-0040.
Anti-H3K79me2: Millipore cat no. 04/835
Anti-H3K27ac: Diagenode cat no. C15410196 lot no. A1723-0041D.
Anti-H3K4me1: Diagenode cat no. pAB-194-050.
Anti-H3K4me3: Diagenode cat no. pAB-003-050.
Anti-ELF1: Bethyl cat no. A301-443A. lot no. 1.
Anti-RUNX2: Cell signalling cat no. 8486 lot no. 1.
Anti-MYB: Abcam cat no. ab177510.
Anti-CTCF. Millipore cat no. 07-729.

Peak calling parameters

Peaks were called using the Homer tool findPeaks, with the input track provided for background correction, using the -style histone or -style factor options to call peaks in histone modification or transcription factor/ATAC datasets, respectively.

Data quality

Reads were filtered to remove PCR duplicates. Called peaks were analyzed as described on homer.ucsd.edu and compared to input track, with a threshold of FDR < 0.001 applied.

Software

Quality control of FASTQ reads, genome alignment, PCR duplicate filtering, blacklisted region filtering and UCSC data hub generation was performed using an in-house pipeline: https://github.com/Hughes-Genome-Group/NGseqBasic/releases
