# Supplementary material for: DOT1L inhibition reveals a distinct subset of enhancers dependent on H3K79 methylation
Source: Nat Commun. 2019 Jun 26;10:2803. doi: 10.1038/s41467-019-10844-3 (PMC6594956; doi:10.1038/s41467-019-10844-3)
Supplement: Supplementary file 4 — Description of Additional Supplementary Files [file 41467_2019_10844_MOESM4_ESM.docx]

**Description of Additional Supplementary Files**

File Name: Supplementary Data 1
Description: Annotated SEM enhancer list containing H3K79me3 KEEs and nonKEEs, with the nearest gene and overlaps with super-enhancers

File Name: Supplementary Data 2
Description: Annotated gene list containing transcriptional response to DOT1Li in SEM cells and KEE/non-KEE assignment in SEM, RS4;11, THP1, ML2, K562, GM12878, HeLa and human embryonic stem cells

File Name: Supplementary Data 3
Description: List of biotinylated Capture-C oligos

File Name: Supplementary Data 4
Description: Annotated SEM ATAC peak list, containing response to DOT1Li, nearest gene and overlap with enhancers

File Name: Supplementary Data 5
Description: Capture-C enhancer-promoter interaction statistics in SEM, RS4;11 and THP1 cells.

File Name: Supplementary Data 6
Description: P-values for statistical tests
