# Supplementary material for: DOT1L inhibition reveals a distinct subset of enhancers dependent on H3K79 methylation
Source: Nat Commun. 2019 Jun 26;10:2803. doi: 10.1038/s41467-019-10844-3 (PMC6594956; doi:10.1038/s41467-019-10844-3)
Supplement: Supplementary file 2 — Peer Review [file 41467_2019_10844_MOESM2_ESM.pdf]

Reviewers' comments:

Reviewer #1 (Remarks to the Author):

In their manuscript "DOT1L inhibition reveals a distinct class of enhancers dependent on H3K79 methylation" Godfrey et al identify a new subset of enhancers that are marked by H3K79me3. The authors demonstrate that genes whose enhancers have the H3K79me3 mark are expressed at higher levels compared to genes that don't have the H3K79me3 enhancers. Moreover, using the DOT1L inhibitor, EPZ-5676 the authors demonstrate that transcription is functionally dependent on the H3K79me3 mark. Their model suggests that H3K79me3 at enhancers mediates chromatin accessibility, transcription factor binding, and ultimately promoter-enhancer interactions, thereby regulating gene expression. While this manuscript does present some interesting new analyses, there are major questions for clarification.

Major Points
1. One major concern is that all the data presented in the manuscript is from one cell line (SEM). This manuscript would be strengthened if some of the key findings, particularly the presence of H3K79me3 at enhancers and the promoter-enhancer interaction (assayed using Capture-C) would be confirmed in another leukemia cell line. There are numerous other MLL-rearranged leukemia cell lines, including some that have the MLL-AF4 rearrangement (e.g. RS4;11 or MV4;11).
2. Another concern is the confounding effect of H3K79me2 on the data analysis. Firstly, it would be useful to know if H3K79me2 is also found in the KEEs? Or is H3K79me2 found at transcription start sites of genes that have KEEs? This is important because the authors use the DOT1L inhibitor EPZ-5676 to ablate H3K79me3 and they conclude that a subset of genes with KEEs displayed reduced transcription upon DOT1L inhibitor treatment, implicating H3K79me3 in enhancer function. However, since DOT1L inhibition also ablates H3K79me2, it would be important to know or at least address how much of these effects are associated with H3K79me2 versus H3K79me3. In other words, are these effects indeed associated with ablation of H3K79me3 at enhancers or potentially due to ablation of H3K79me2 at transcriptional start sites? Also, throughout the manuscript the authors imply that inhibition of DOT1L and thus H3K79me shows that changes in H3K79me are the cause of the gene expression changes identified. This is actually an association and it still remains possible that loss of modification of some other DOT1L substrate is critical. This should at least be acknowledged somewhere.
3. The last part of the last sentence in the abstract should be removed since there is no data concerning phase-separation in this paper.

Minor Points
1. For Figure 1 di/dii it would be useful to include another example. In the last figure, Figure 6, the authors show BCL11A as another KEE-associated gene – it would be more convincing if the authors included BCL11A (or some other representative KEE-regulated gene) in addition to ARID1A throughout the manuscript.
2. In the paragraph where the authors discuss super-enhancers and how the KEE enhancers are distinct from super-enhancers, the authors say " We identified super-enhancers in MLL-AF4 cells as in the original study and found that most KEEs are not super-enhancers" – there should be a citation after 'original study'
3. In the subsequent paragraph the authors discuss a previous publication in which they identified H3K79me3 spreading across genes–they state that the majority of KEE genes are not H3K79me3 spreading targets. To my knowledge, the referenced study actually primarily looks at H3K79me2. Do KEE-regulated genes correlate with the spreading of H3K79me2?
4. For Figure 2d – the authors just say 'H3K79me3-marked' – are these marks at the enhancers or the transcriptional start sites? How do the numbers change when just looking at H3K79me3 at the enhancers?
5. For Figure 2eii it would be important that the authors confirm their RNA-seq findings (i.e. reduction

of ARID1B transcription) by qPCR.
6. For Figure 3 ei/eii it would be good to show qPCR data confirming the downregulation of BCL2 (in the supplementary Figure it only shows the RNA-seq tracks).
7. In Figure 4b the authors show that KEE genes have lower levels of H3K27ac compared to non-KEE genes – doesn't this contradict their statements in Figure 1g, where they say that KEE genes have higher levels of H3K27ac?
8. The 'condensates' part of Figure 7 should be removed since there is no data in the manuscript indicating that KEE-promoter interactions form condensates.

Reviewer #2 (Remarks to the Author):

This is an interesting study which identifies a set of enhancers marked by H3K79me3 in leukemia cells. Although the experiments are well performed, a number of modifications would improve the manuscript as follows:
-Although the data presented in the manuscript are comprehensive, currently there is no functional evaluation of any individual "KEE" enhancer (e.g. through targeted genetic disruption) to actually study its functional impact individually on gene expression or other biological readouts.
-The manuscript text needs to state explicitly what cell types were used for each experiment in the results section and the genetic background of these cells (particularly whether the cells are MLL rearranged or not).
-The above comment is particularly importance as it is unclear if these H3K79me3 enhancers are specific to MLL rearranged leukemia versus more other genetic subtypes of leukemia.
-The comments in the manuscript about H3K79me3 enhancers being important for promoting phase separation aren't evaluated by any experiments directly and seem to come from nowhere in the Abstract and model cartoon in Figure 7. Unless the authors have actual data regarding this class of enhancers on regulating phase separation, these comments should be deleted from the manuscript.
-Figure 1a diagram is not helpful. It isn't clear what is being shown as different from "KEE" and "non-KEE" enhancers in this diagram and it is not ideal to start out the manuscript with a cartoon model that has yet to be substantiated by any data in the manuscript. Also the results section describing analysis of chrom HMM data omitted use of anti-H3K79me3 ChIP-seq (which appears to be an oversight).
-What is the rationale for evaluation of H3K79me3 as opposed to H3K79me2? Would the results expected to be different using anti-H3K79me2 ChIP-seq?

Reviewer #3 (Remarks to the Author):

This is a manuscript by Godfrey et al. entitled, "DOT1L inhibition reveals a distinct class of enhancers dependent on H3K79 methylation". The premise of the article suggests that a distinct subset of enhancers in leukemia cells are functionally dependent upon H3K79me3 enrichment. The authors make use of the DOT1L inhibitor, EPZ-5676, emphasizing that the loss of H3K79me3 at these H3K79me3 enhancer elements (KEEs) leads to altered chromatin accessibility, histone acetylation and transcription factor binding. Followed by using a modified 3C approach, to show that H3K79me3 is essential for KEE interactions with the promoter as well as transcription of the associated genes. Overall, the concept is compelling; however, some weaknesses in the data presented make it difficult to clearly state whether H3K79me3 truly represent a subclass of enhancers. Nonetheless, there are some notable strengths to the approaches used and their results that suggest the significance of the H3K79me3 mark in gene control in leukemia cells as a potential hallmark of some enhancers.

Among the authors' main figures, they performed many comparisons however, several of these

comparisons are not clear and lack rationale for their comparisons. There are a number of insignificant (or less convincing) plots (discussed below) that could be placed in Supplementary data. Whereas, having these figures within the main body of the manuscript detract from the authors' overall hypothesis and make for a less convincing argument (i.e. ATAC-Seq, some controls, etc.), or preferred to just omit.

This reviewer's belief is if the authors want to explore H3K79me3 functionality, it appears they have sufficient ChIP-Seq, RNA-Seq, and their modified 3C data to reach some modest conclusion for a gene regulatory role through H3K79me3 enrichment. However, the concept of KEE as a subclass of enhancers remains premature to claim from the data provided and for many of the figures the case for regulatory elements associated with a KEE is not made particularly strong.

Specific Comments:
In Figure 1C: The figure notes that 2460 gene have the KEE signature in their enhancers, this apparently doesn't necessarily indicate that "KEE may DIRECTLY regulate the expression of a subset of genes" as the authors state and claim. Combined with Figure 1D, the authors, again, use the characterization of "directly". The KEE association in this instance maybe involved, but the use of "direct" is not an accurate characterization as presented.

Figure 1E and 1F: While the comparison between the KEEs with non-KEEs makes sense, the comparison of genes with a KEE versus those with no enhancer is not relevant. Then in Figure 1F, the authors divided each into three categories, high, medium and low H3K79me3 enrichment within a gene body which lack the appropriate context for comparisons, if their goal is about H3K79me3 enrichment at enhancer regions, having this information of H3K79me3 within gene body in this plot is very confusing and a poor comparison, and the mixture of these different gene contexts of those two patterns of H3K79me3 enrichment confuses the intent of the studies performed.

Figure 1G: A KEE-containing gene, as is indicated in the name, already has the enrichment of H3K79me3 at their enhancers (most of which are intragenic, within the gene body, anyway), that means, the KEE-containing genes, are supposed to be enriched with higher levels of H379me3 in the gene body, regardless. Therefore, many of the plots within this figure panel are not relevant to the intended conclusion. While the box plot shows statistical significance, the differences do not appear dramatic between the KEE-containing gene and "no enhancer" groups. Also noted, H3K27ac and H3K4me1 -marked enhancers should also contain these two notable histone modifications in KEE and no-KEE containing genes. Therefore, it is not clear what the authors are comparing among those enhancers with the dual H3K27ac/K4me1 reads versus those with "no enhancer also carrying the dual H3K27ac/K4me1 enrichments. This doesn't help the authors with their argument.

The authors should consider removing Supplementary Figure 1A, as it is redundant with Figure 1b, and should be deleted. This may confuse readers.

Shown in Supplemental figure 1C: Is it H3K79me in total the authors are measuring or is it a typo? Should it be H3K79me3? Besides, total methylation was not mentioned anywhere it in the text.

Supplemental figure 1 D: See a presumed typo: KEE gene, not "HEE".

On Page 6, In the end, the authors claim, "MLL-AF4 fusion spreading targets are enriched for KEES compared to non-spreading target", The authors didn't indicate which specific plot this result was referring to in Supplementary Figure 1. The reviewer presumes Supplementary Figure 1G, but this figure lacks evidence for the "non-spreading" targets. Overall, MLL-AF4 study appears redundant, since the authors are not using the same cell line model for the remainder of the studies performed, and the only piece of evidence associated with their result is not convincing.

Shown in Figure 2D: The authors need to clarify what represents enrichment of the H3K79me3 mark

within gene bodies and H3K79me3 within enhancers (or KEEs). The reviewer suggests that it would be more meaningful, if the comparison made are between KEE-associated versus non-KEE genes, but shouldn't involve genes enriched for H3K79me3 versus those lacking H3K79me3.

Shown in Figure 2F: the pie chart is misleading, as the absolute values of KEE-containing, non-KEE, and the non-enhancer groups are drastically different (i.e. 2000~, 1300~, 3500~). Therefore, comparisons made using different absolute numbers, as shown, should somehow be better normalized to reflect the changes in percentage for each of these distinct categories.

Shown in Figure 2h: it is apparent that not even KEE-containing genes have a decrease in gene expression (logFC). This result contradicts what the authors claimed. Shown in Figure 2I it is not apparent why the authors measure K79me3 at the TSS, if it doesn't show results concordant with their hypothesis it shouldn't be presented. The authors make the statement, " K79me3 levels are the highest at KEE gene" in the text, which is not relevant to this plot shown (only represents KEE-associated genes localized within the TSS region). Also, the authors only measure the levels of H3k79me3 within the gene bodies (which lacks clear justification and should maybe included as supplementary data).

Shown in Figure 3b, the authors demonstrate only a 10% decrease in ATAC peak calls even for KEE-associated genes, therefore make the evidence presented weak. Furthermore, all the ATAC-Seq attempts to establish that H3K79me3 plays a fundamental role in chromatin accessibility needs more evidence to convince readers.

Shown in Figure 3 ei, the authors use the BCL2 gene as an example, which the authors neglected to mention in the text until describing in a later figure stating this gene is also slightly decreased. The authors should make some of this finding in the results section.

Shown in Figure 4c, the authors show the KEE-associated group that upon adding the DOT1L inhibitor, the comparison for H3K27ac enrichment and the ATAC profile is very confusing to the reader. Therefore, the concept of how the K27ac and K79me3 together orchestrate chromatin accessibility is not convincing. Maybe the authors should try not to conflate the two signatures within one profile and separate this as two distinct patterns each in different plots.

Shown in Figure 5e, shown within the bar graph, only the ELF1 group in ARID1B iare indicated as statistically significant shown with asterisks, however, it is not clear whether the H3K27ac, H3K4me1 and H3K79me3 group for this gene reach significance. This is especially important for illustrating the enrichment for H3K27ac, as the authors claim this being significant in Figure 4d. This detracts from the authors' claim.

Shown in Figure 6E the authors show among the downregulated group of genes, BCL2, which is a non-KEE gene throughout the manuscript, the plot for BCL2 has circle dots representing KEE enhancer, shouldn't this BCL2 gene be excluded from having any KEE enhancer? Otherwise, having at least one KEE enhancer suggests BCL2 is a KEE-associated gene. Is this not correct? If this is the case throughout the whole manuscript, the authors are using flawed negative control as a "non KEE" gene, therefore, making all of those comparisons improper. Is there an explanation for this?

In summary, the reviewer suggests the authors reorganize their figures and re-prioritize some data, and to remove unnecessary data, which further weaken the manuscript or at the very least embed such ancillary data within supplementary information if it can be justified for their use.

General comments for all reviewers

Overall, we'd like to thank all the reviewers for their hard work on assessing the first version of our paper and for their positive comments about the work. We would also like to thank the reviewers for their insights and critiques which have helped us improve the paper. We hope that this revised version of the paper will answer most of the questions and concerns raised.

All of our comments to reviewers below (with the exception of figure legends) are highlighted in red to make them easier to follow. We have rewritten the text of the manuscript extensively so it would reduce clarity to highlight all the changes we have made in the manuscript. However, where appropriate we have highlighted in red key changes made to the manuscript in response to specific reviewer comments. We also reproduce these changes below.

There are two general issues raised by all reviewers:

1) The first is the difference between H3K79me2 and H3K79me3. We address this issue in more detail in the paper and in response to the specific reviewer comments below, but the main conclusion is that within the limits of antibody specificity, we do not see a difference between H3K79me2 and H3K79me3 patterns of distribution. Therefore, we cannot address whether these two marks have distinct functions in MLLr leukemia cells and so refer to H3K79me2/3 in the paper.

2) Another issue that has been raised is whether the KEEs we studied are functional enhancers. Although it is not possible to address this issue for every single KEE, we have deleted parts of two KEEs at *ARID1B* and *CDK6* in their endogenous context and shown that they are important for maintaining expression of their associated genes. We also used a luciferase assay to show that part of the *ARID1B* and *JMJD1C* KEE sequences can function as enhancers.

We address specific reviewer comments in more detail below:

Reviewer #1 (Remarks to the Author):

In their manuscript "DOT1L inhibition reveals a distinct class of enhancers dependent on H3K79 methylation" Godfrey et al identify a new subset of enhancers that are marked by H3K79me3. The authors demonstrate that genes whose enhancers have the H3K79me3 mark are expressed at higher levels compared to genes that don't have the H3K79me3 enhancers. Moreover, using the DOT1L inhibitor, EPZ-5676 the authors demonstrate that transcription is functionally dependent on the H3K79me3 mark. Their model suggests that H3K79me3 at enhancers mediates chromatin accessibility, transcription factor binding, and ultimately promoter-enhancer interactions, thereby regulating gene expression. While this manuscript does present some interesting new analyses, there are major questions for clarification.

We'd like to thank the reviewer for their helpful comments and questions and for thinking that our manuscript presents some interesting new analyses.

Major Points

1. One major concern is that all the data presented in the manuscript is from one cell line (SEM). This manuscript would be strengthened if some of the key findings, particularly the presence of H3K79me3 at enhancers and the promoter-enhancer interaction (assayed using Capture-C) would be confirmed in another leukemia cell line. There are numerous other MLL-rearranged leukemia cell lines, including some that have the MLL-AF4 rearrangement (e.g. RS4;11 or MV4;11).

This is an important point and we have performed two sets of experiments to address this. First, we performed additional H3K79me2 and H3K79me3 ChIP-seq and used published datasets as input for ChromHMM to identify putative KEEs in multiple additional cell types and cell lines (new analysis, Fig. 1b, c; and see below).

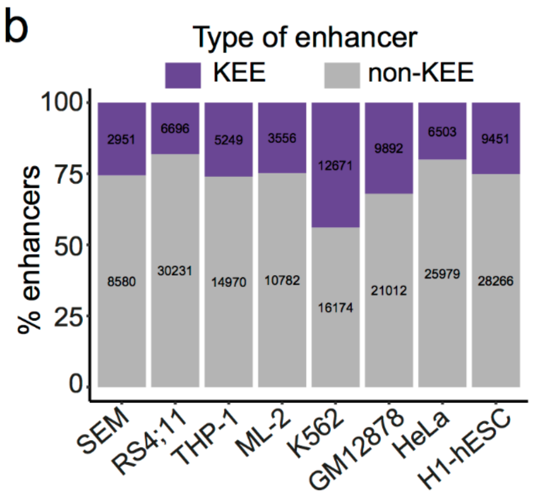

Figure 1 (b) Proportion of predicted enhancers which are KEEs (purple) or non-KEEs (gray) in different cell types, based on ChromHMM analysis.

Second, we have performed Capture-C at 64 loci (targeting both KEE-associated and non-KEE-associated genes) in SEM cells and at 32 loci (KEEs and non-KEEs) in two other cell lines: RS4;11 (MLL-AF4) and THP1 (MLL-AF9) (new data in Fig. 1, 5 and 6, Supplementary Fig. 1, 5 and 6, Supplementary Tables 3 and 5).

A summary of the significant changes in promoter-enhancer interactions following DOT1Li in all three cell lines is shown below. The main message is that many of the KEE-promoter interactions tested appear to be very sensitive to a loss of H3K79me2/3 in SEM and RS4;11 cells, whereas the non-KEE-promoter interactions are not. In THP1 cells, there were specific KEEs that were sensitive to a loss of H3K79me2/3 but the effect was more subtle. There are two possible explanations for this. One is that H3K79me2/3 is more functionally important at KEEs in MLL-AF4 rather than MLL-AF9 cells. Another explanation is that, since the choice of Capture-C probes was based on the KEE analysis from SEM cells, we were not analysing the most appropriate KEE-associated genes in THP1 cells. The potential tissue/cell type specificity of KEE activity is in line with the observation that although H3K27ac and H3K4me1 can mark potentially active enhancers, not all loci carrying these modifications are functional. Here, we believe that H3K79me2/3 doesn't necessarily directly drive enhancer function, but provides an opportunity for transcription factors to bind, depending on tissue-specific gene expression. We discuss these points extensively in the manuscript, but why some KEEs are active in some cell types but not others is an important issue we wish to address in future studies.

Figure 6

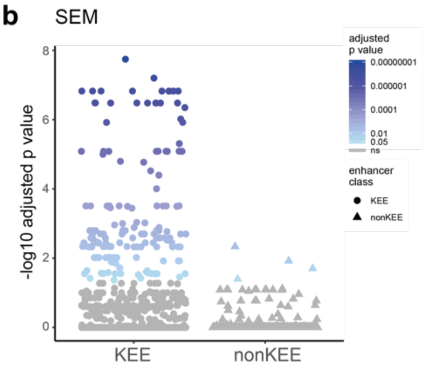

Figure 6

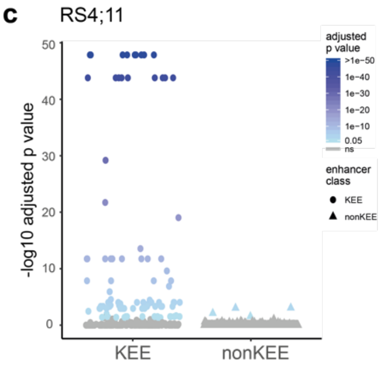

Supplementary Figure 6

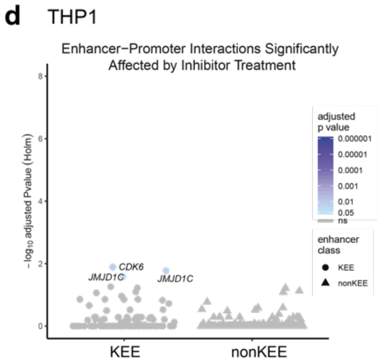

Figure 6. (b-c) Statistical analysis of the significance of the change in enhancer-promoter Capture-C interactions following DOT1Li in SEM and RS4;11 cells. Each point represents the interaction of a KEE (circle) or non-KEE (triangle) with a gene promoter. Holm-Bonferroni adjusted p-values were calculated following a Wilcoxon rank test (n=3). **Supplementary Figure 6. (d)** Statistical analysis of the significance of the change in enhancer-promoter Capture-C interactions following DOT1Li in THP1 cells. Each point represents the interaction of a KEE (circle) or non-KEE (triangle) with a gene promoter. Significantly affected interactions are labelled with the relevant gene promoter. Holm-Bonferroni adjusted p-values were calculated following a Wilcoxon rank test (n=3).

2. Another concern is the confounding effect of H3K79me2 on the data analysis. Firstly, it would be useful to know if H3K79me2 is also found in the KEEs? Or is H3K79me2 found at transcription start sites of genes that have KEEs? This is important because the authors use the DOT1L inhibitor EPZ-5676 to ablate H3K79me3 and they conclude that a subset of genes with KEEs displayed reduced transcription upon DOT1L inhibitor treatment, implicating H3K79me3 in enhancer function. However, since DOT1L inhibition also ablates H3K79me2, it would be important to know or at least address how much of these effects are associated with H3K79me2 versus H3K79me3. In other words, are these effects indeed associated with ablation of H3K79me3 at enhancers or potentially due to ablation of H3K79me2 at transcriptional start sites? (second question answered further below)

Again, this is an important point also brought up by the other reviewers. We analysed both H3K79me2 and H3K79me3 ChIP-seq datasets in SEM cells and found that these two marks show a very tight correlation, and both marks overlap almost perfectly genome wide. If we call KEEs using either ChIP-seq dataset, we get almost identical results (Fig. 1a and Supplementary Fig. 1a; see below). Visually, the two marks also display patterns that are almost identical to each other (e.g. Fig. 1e). Due to this very strong overlap, the reviewer is correct that we cannot differentiate the function of H3K79me2 from H3K79me3, and we reflect this by referring to H3K79me2/3-marked genes and enhancers throughout the paper.

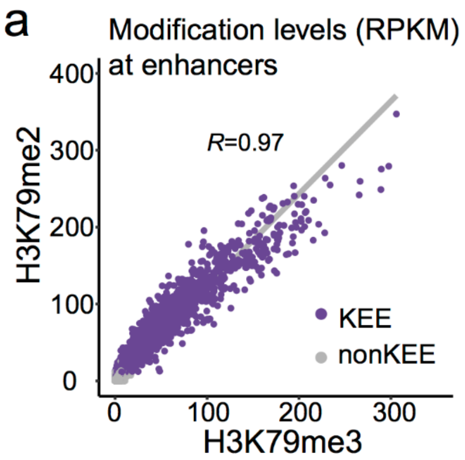

Fig. 1. (a) Correlational analysis between H3K79me2 ChIP-seq and H3K79me3 ChIP-seq reads in SEM cells at KEEs (purple) and non-KEEs (gray), data based upon one replicate of both H3K79me2 and H3K79me3 ChIP-seq.

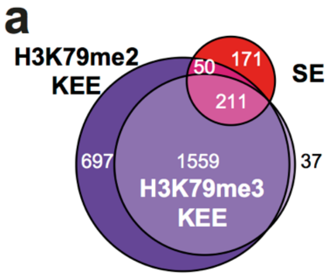

Supplementary Fig. 1. (a) Overlap between KEEs identified using H3K79me2 and H3K79me3 ChIP-seq in SEM cells with super-enhancers (SE).

The antibody we use for H3K79me3 ChIP-seq was recommended by BLUEPRINT for ChIP-seq and the specific lot number we used displays a specificity for H3K79me3 over H3K79me2 in a dot blot analysis (http://antibody.uni-saarland.de/antibody/112/Diagenode_company/H3K79me3/). The H3K79me2 antibody we used has been previously tested for specificity over H3K79me3 in ChIP (Steger et al, Mol Cell Biol, 2008). That said, we cannot completely rule out cross reactivity. To reflect this, we have put this section in the discussion:

"In yeast, H3K79me2 and H3K79me3 display different distribution patterns {Schulze, 2009}, but past work in mammalian cells has suggested that these two modifications may overlap {Steger, 2008;Kerry, 2017}. Our analyses demonstrate a strong correlation between H3K79me2 and H3K79me3, suggesting that at least in MLL-AF4 cells they may be functionally equivalent. The H3K79me3 antibody used in our work here (Diagenode C15410068, lot A246-0040, recommended by the Blueprint Consortium for ChIP-seq) shows a high degree of specificity for H3K79me3 and not H3K79me2 in a dot blot analysis (http://antibody.uni-saarland.de/antibody/112/Diagenode_company/H3K79me3/), and the H3K79me2 antibody we use has been shown to be specific for H3K79me2 in

ChIP experiments {Steger, 2008 }. This provides some level of confidence that our ChIP-seq results are not a consequence of antibody cross-reactivity, as has been noted for a different H3K79me3 antibody {Steger, 2008 }. However, since the nature of the antibody-epitope interaction differs between applications, we cannot completely rule out the possibility of cross-reactivity in our ChIP experiments."

In other words, are these effects indeed associated with ablation of H3K79me3 at enhancers or potentially due to ablation of H3K79me2 at transcriptional start sites?

This is an interesting point; whether the changes in gene expression could be due to an effect of H3K79me2/3 loss in the gene body (i.e. implying a role in transcription elongation) versus the effect on enhancers. Since these two functions are likely intertwined (especially considering most KEEs are intragenic) they are difficult to separate. However, our analysis of enhancer features suggests that H3K79me2/3 does contribute specifically to enhancer function. This is best illustrated by the following two points:

1) Throughout the paper, we have specifically excluded genes which do not contain H3K79me2/3 within the gene body (see Fig. 2d). By focusing on comparing H3K79me2/3-marked non-KEE genes with H3K79me2/3-marked KEE genes we minimise any differential effect of loss gene body H3K79me2/3 between the two groups following DOT1Li. If H3K79me2/3 was important for transcription elongation alone, you would expect our analysis for these two gene sets to be quite similar. Instead, we observed differences in the sensitivity of these gene sets to loss of H3K79me2/3 (see Fig. 2f below).

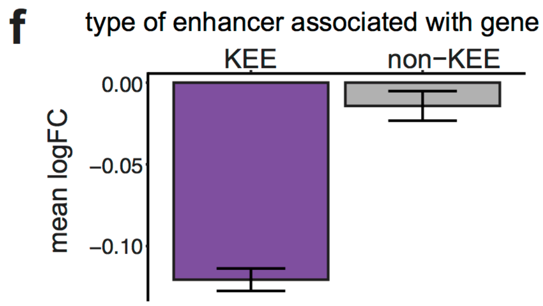

Figure 2. (f) Mean logFC of H3K79me2/3-marked genes associated with a KEE (purple) or non-KEE (gray) by nascent RNA-seq (**** = p-value<0.0001, Fisher's exact test, n=3).

2) Our H3K27ac ChIP-seq (see Fig. 3a below) and Capture-C (see Fig. 6b and c; see above) results all indicate a specific loss of enhancer activity following DOT1Li at KEEs but not non-KEE enhancers, arguing for a direct role for H3K79me2/3 at these loci.

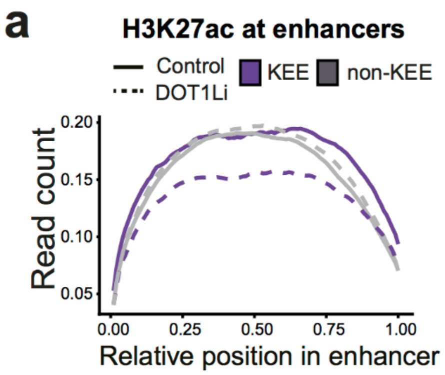

Figure 3. (a) Metaplot of H3K27ac ChIP-seq signal across KEEs (purple) or non-KEEs (gray) in control (solid line) and DOT1Li (dashed line) in SEM cells, based upon one replicate.

Also, throughout the manuscript the authors imply that inhibition of DOT1L and thus H3K79me shows that changes in H3K79me are the cause of the gene expression changes identified. This is actually an association and it still remains possible that loss of modification of some other DOT1L substrate is critical. This should at least be acknowledged somewhere.

We have added this paragraph in the discussion to acknowledge this point:

"While DOT1L is the only enzyme known to catalyse H3K79 methylation, we cannot rule out the possibility that DOT1L has other methylation targets, and the effects we are observing are catalysed by methylation of a non-histone substrate. However, even if this is true, any such alternative target would have to also somehow function specifically at KEEs, with no activity at non-KEEs. Formally, this remains a possibility, however."

3. The last part of the last sentence in the abstract should be removed since there is no data concerning phase-separation in this paper.

We agree and have removed this line from the abstract.

Minor Points

1. For Figure 1 di/dii it would be useful to include another example. In the last figure, Figure 6, the authors show BCL11A as another KEE-associated gene – it would be more convincing if the authors included BCL11A (or some other representative KEE-regulated gene) in addition to ARID1A throughout the manuscript.

We have added multiple examples of KEEs in the main figures and supplementary figures in these places:

Fig. 1f – Capture-C trace figures across *ARID1B* KEEs in SEM and THP1 cells
Supplementary Fig. 1 – Examples of *CDK6*, *JMJD1C* and *BCL11A* KEEs in SEM, RS4;11 and THP1 cells and a comparison of *ARID1B* across all three cell types.
Supplementary Fig. 5 – Examples of Capture-C enhancer-promoter interaction changes at *BCL11A, CDK6* and *JMJD1C* KEEs in SEM and RS4;11 cells
Supplementary Fig. 6 – Examples of Capture-C at *CDK6* and *JMJD1C* KEEs in THP1 cells.

2. In the paragraph where the authors discuss super-enhancers and how the KEE enhancers are distinct from super-enhancers, the authors say " We identified super-enhancers in MLL-AF4 cells as in the original study and found that most KEEs are not super-enhancers" – there should be a citation after 'original study'

We have added a reference to Lovén *et al* 2013 and Whyte *et al* 2013 which are the original super-enhancer papers and the sentence now states the following:

"Importantly, although KEEs overlap with some super-enhancers {Whyte, 2013;Loven, 2013;Hnisz, 2013} in SEM cells, most KEEs are not super-enhancers and many super-enhancers are not KEEs (Supplementary Fig. 1a, b, Supplementary Table 1). This suggests that at least in human MLL-AF4 (SEM) leukemia cells, KEEs are a distinct subset of enhancers that can be identified with either H3K79me2 or H3K79me3."

3. In the subsequent paragraph the authors discuss a previous publication in which they identified H3K79me3 spreading across genes–they state that the majority of KEE genes are not H3K79me3 spreading targets. To my knowledge, the referenced study actually primarily looks at H3K79me2. Do KEE-regulated genes correlate with the spreading of H3K79me2?

The reviewer is correct that our previous work focused mainly on H3K79me2 as a marker of spreading, although we did find some evidence that spreading targets were marked by H3K79me3 as well (Figure S7a of Kerry et al 2017). As mentioned above, we have expanded on our analysis and shown that H3K79me2 and H3K79me3 correlate very strongly with each other (Fig. 1a and Supplementary Fig. 1a). Essentially, almost all KEEs called with H3K79me2 are the same as KEEs called with H3K79me3. We performed a specific overlap with spreading targets and found almost no difference between the two marks (see Supplementary Fig. 1d, e; see below).

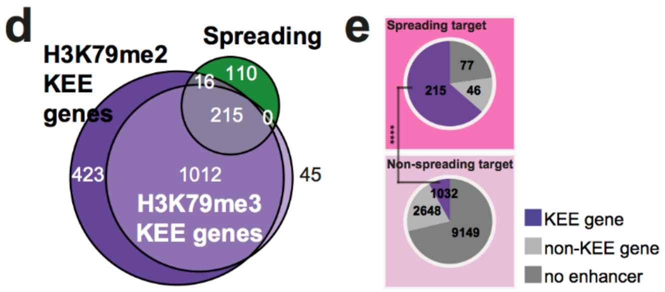

Supplementary Figure 1 (d) Overlap between KEE-genes identified using H3K79me2 and H3K79me3 ChIP-seq in SEM cells and spreading target genes. **(e)** Proportion of MLL-AF4 spreading genes that are also a KEE-gene (purple), non-KEE-gene (gray), or not annotated with an enhancer (light gray) (**** = p <0.0001, Fisher's exact test) in SEM cells.

4. For Figure 2d – the authors just say 'H3K79me3-marked' – are these marks at the enhancers or the transcriptional start sites? How do the numbers change when just looking at H3K79me3 at the enhancers?

Fig. 2d has been re-labelled with the phrase "H3K79me2/3 in gene body" to clarify our intent here. This figure shows all genes, colored based on whether or not they are marked in the gene body with H3K79me2/3 and whether they are downregulated, upregulated or unchanged in their expression. The figure simply is intended to illustrate that downregulated genes are more likely to be marked with H3K79me2/3 in the gene body.

We address the second question in Fig. 2e and 2f; when we separate H3K79me2/3-marked genes into those associated with KEEs and non-KEEs, KEE genes are much more likely to display downregulation. This indicates that the association of a gene with a KEE contributes to the sensitivity of genes to the loss of H3K79me2/3, independently of the presence of H3K79me2/3 in the gene body.

5. For Figure 2eii it would be important that the authors confirm their RNA-seq findings (i.e. reduction of ARID1B transcription) by qPCR.

To address this, we have performed qRT-PCR on total RNA in untreated vs DOT1Li treated cells at three KEE (*ARID1B*, *CDK6*, *BCL11A*) and one non-KEE gene that has H3K79me2/3 in the gene body (*BCL2*). This new data is presented below and in Supplementary Fig. 2f.

f

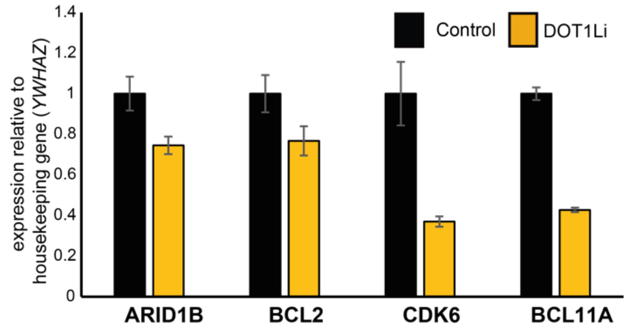

Supplementary Fig. 2. (f) RT-qPCR of total RNA in control (black) and DOT1Li (orange) SEM cells at *ARID1B*, *BCL2*, *CDK6* and *BCL11A*, normalised to the housekeeping gene *YWHAZ*. Error bars represent standard deviation

6. For Figure 3 ei/eii it would be good to show qPCR data confirming the downregulation of BCL2 (in the supplementary Figure it only shows the RNA-seq tracks).

We have performed RT-qPCR on total RNA for *BCL2* (see Supplementary Fig. 2f, shown above).

7. In Figure 4b the authors show that KEE genes have lower levels of H3K27ac compared to non-KEE genes – doesn't this contradict their statements in Figure 1g, where they say that KEE genes have higher levels of H3K27ac?

We'd like to thank the reviewer for pointing this out. This apparent contradiction was partially caused by an error in our analysis. We have reanalysed our data and found that H3K27ac across the entire enhancer is present on average at roughly similar levels at all KEEs versus non-KEEs (See Fig. 3a below). However, when you look at H3K27ac specifically around DOT1Li "decreased" ATAC peaks, H3K27ac levels are generally higher at decreased ATAC peaks in KEEs versus non-KEEs (See Supplementary Fig. 3i, below).

We've commented on the ATAC data further in the results:

"We note that DOT1Li-sensitive ATAC peaks in KEEs show a higher level of H3K27ac compared to those within non-KEEs (Supplementary Fig. 3i), in contrast to the similar H3K27ac levels across KEEs and non-KEEs (Fig. 3a). This is likely because the ATAC peaks take up only a small proportion of the enhancers."

Figure 3

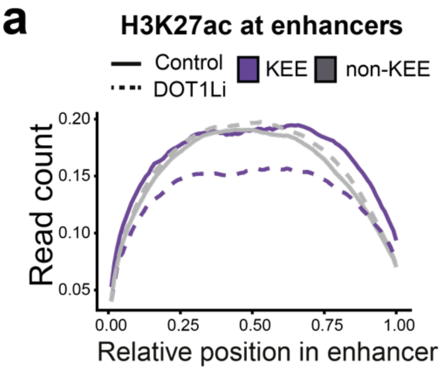

Supplementary Figure 3

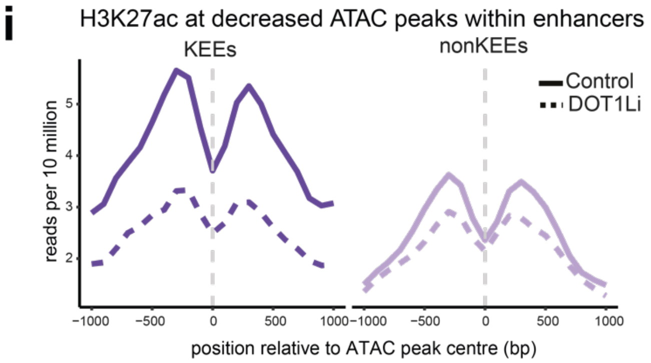

Figure 3. (a) Metaplot of H3K27ac ChIP-seq signal across KEEs (purple) or non-KEEs (gray) in control (solid line) and DOT1Li (dashed line) in SEM cells, based upon one replicate.
Supplementary Figure 3. (i) Metaplot of H3K27ac ChIP-seq levels at DOT1Li-decreased ATAC peaks found within KEEs and non-KEEs. H3K27ac levels at these regions were measured from control (solid line) and DOT1Li (dashed line) SEM cells.

8. The ‘condensates’ part of Figure 7 should be removed since there is no data in the manuscript indicating that KEE-promoter interactions form condensates.

This was speculation on our part so we have removed it from the figure.

Reviewer #2 (Remarks to the Author):

This is an interesting study which identifies a set of enhancers marked by H3K79me3 in leukemia cells. Although the experiments are well performed, a number of modifications would improve the manuscript as follows:

-Although the data presented in the manuscript are comprehensive, currently there is no functional evaluation of any individual "KEE" enhancer (e.g. through targeted genetic disruption) to actually study its functional impact individually on gene expression or other biological readouts.

We'd like to thank the reviewer for their valuable comments and we are glad that they find our study interesting.

The reviewer raises the very important point that in the original study we had not shown that any KEEs were actually functional enhancers. To address this question, we decided to take two approaches: 1) cloning of KEE sequences into a luciferase construct to test if they can enhance transcription from a minimal promoter; and 2) targeted deletion of KEEs within their genomic context to determine if this would disrupt gene expression.

In short, our new results have shown that a sequence from the *ARID1B* KEE and *JMJD1C* KEE can function as an enhancer in a luciferase assay and deletions in *ARID1B* and *CDK6* KEEs are sufficient to disrupt transcription of the genes (new data Fig. 2a and Supplementary Fig. 2a; see below).

a

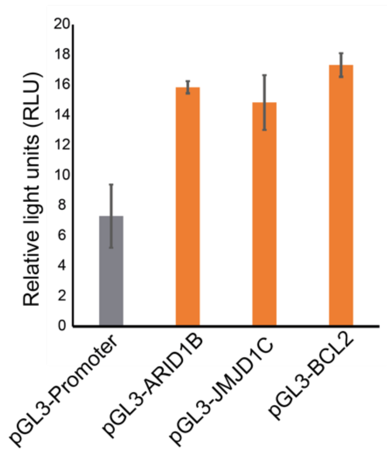

Supplementary Figure 2. (a) Luciferase assay for putative enhancer activity. pGL3 plasmid containing promoter alone, or sections of the *ARID1B* or *JMJD1C* KEE, or *BCL2* non-KEE were co-transfected with pRL-TK *Renilla* into 293T cells. Firefly luciferase activity was normalised to *Renilla* luciferase. Error bars represent standard deviation of three technical replicates.

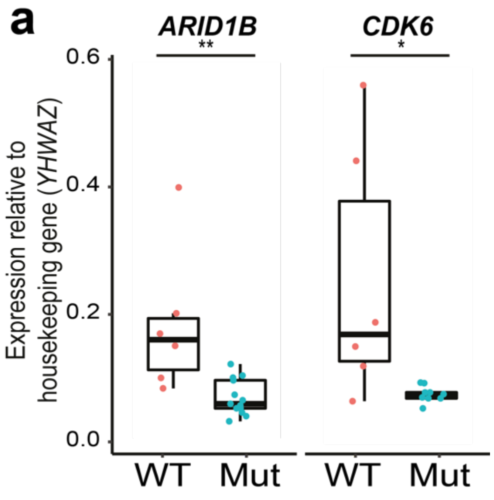

Figure 2. (a) Gene expression in wildtype compared to *ARID1B* and *CDK6* enhancer mutant SEM clones, normalised to the housekeeping gene *YWHAZ*. Each point represents a biological replicate. Mann Whitney U test, ** = p<0.05 based upon 7 biological replicates.

-The manuscript text needs to state explicitly what cell types were used for each experiment in the results section and the genetic background of these cells (particularly whether the cells are MLL rearranged or not).

We have carefully gone through the text of the paper and specifically indicated what cells/lines were used in each figure. Other than the ChromHMM analysis showing putative KEEs in multiple cell types (Fig. 1b and c), most of the other primary work has been performed in SEM (MLL-AF4 B-ALL cell line), RS4;11 (MLL-AF4 B-ALL cell line) and THP1 (MLL-AF9 AML cell line) cells. One minor exception is that there is a single ChIP qPCR experiment (Supplementary Fig. 3d) in K562 cells (BCR-ABL CML cell line). This section has been clairified in the methods:

"**Cell culture and cell lines.** SEM (MLL-AF4 B cell ALL line) cells (Greil et al., 1994) and ML-2 (MLL-AF6 AML cell line) cells were purchased from DSMZ (www.cell-lines.de) and cultured in IMDM media with 10% FBS and Glutamax. RS4;11 (MLL-AF4 B cell ALL line), THP1 (MLL-AF9 AML cell line) and K562 (BCR-ABL CML cell line) cells were purchased from ATCC (www.lgcstandards-atcc.org) and cultured in RPMI 1640 with 10% FBS and Glutamax. 293T cells were purchased from ATCC and cultured in DMEM media with 10 % FBS and Glutamax."

-The above comment is particularly importance as it is unclear if these H3K79me3 enhancers are specific to MLL rearranged leukemia versus more other genetic subtypes of leukemia.

This is an important point. In response to other reviewers, we have shown that there is a very strong correlation between H3K79me2 and H3K79me3 in genes/enhancers as well as patterns of distribution (see also below). Because of this, we were able to expand our analysis to enhancers marked with H3K79me2 which allowed us to take advantage of multiple published datasets in other cell types. Using ChromHMM, we were able to show that KEEs are present in multiple cell types including hES cells and non MLL leukemia cell types. We focused the bulk of our functional analysis on MLL-r cell lines (SEM, RS4;11 and THP1) but did perform some preliminary ChIP experiments in K562 cells. In this analysis, KEEs in RS4;11 and SEM cells (both MLL-AF4 B-ALL cell lines) appeared to be particularly sensitive to loss of H3K79me2/3. Whether this is a unique property of MLL-AF4 leukemias or because of differential enhancer usage due to cell type-specific transcription factor expression is unclear. We discuss this extensively in the paper and further expanding this analysis to other cell types is something we will pursue in future studies.

-The comments in the manuscript about H3K79me3 enhancers being important for promoting phase separation aren’t evaluated by any experiments directly and seem to come from nowhere in the Abstract and model cartoon in Figure 7. Unless the authors have actual data regarding this class of enhancers on regulating phase separation, these comments should be deleted from the manuscript.

The other reviewers have raised similar concerns about this and we have removed this as it was highly speculative on our part.

-Figure 1a diagram is not helpful. It isn’t clear what is being shown as different from “KEE” and “non-KEE” enhancers in this diagram and it is not ideal to start out the manuscript with a cartoon model that has yet to be substantiated by any data in the manuscript. Also the results section describing analysis of chrom HMM data omitted use of anti-H3K79me3 ChIP-seq (which appears to be an oversight).

We agree with this and have removed the model figures from throughout the paper. They were intended to be a guide for the reader to show the context for the experiments but they were not effective. We have clarified the ChIP-seq datasets used for the ChromHMM analysis in the figure legends and results.

-What is the rationale for evaluation of H3K79me3 as opposed to H3K79me2? Would the results expected to be different using anti-H3K79me2 ChIP-seq?

This is an important point raised by other reviewers. We initially focused mainly on H3K79me3 because of the published paper from Bonn et al (Nat Gen 2012) that focused on this mark being an enhancer mark. We didn't intend to imply with our original analysis that H3K79me3 was necessarily functionally distinct from H3K79me2, and now refer to H3K79me2/3 where appropriate throughout the paper.

As discussed in our response to reviewer 1, we have now performed an extensive H3K79me2 and H3K79me3 ChIP-seq analysis in SEM cells and found that these two marks show a very tight correlation (Fig. 1a and below) and that both marks overlap almost perfectly genome wide. In addition, we have repeated the ChromHMM analysis in SEM cells using either H3K79me2 or H3K79me3 ChIP-seq to identify KEEs, and the two identify a very similar set of KEEs (Supplementary Fig. 1a; see below). Visually, the two marks display ChIP-seq patterns that are almost identical (Fig. 1e and others). We don't have H3K79me3 ChIP-seq in other cell types because in most cases only H3K79me2 data is publicly available, but we think that the distribution of the two marks is virtually indistinguishable, at least in leukemia cells.

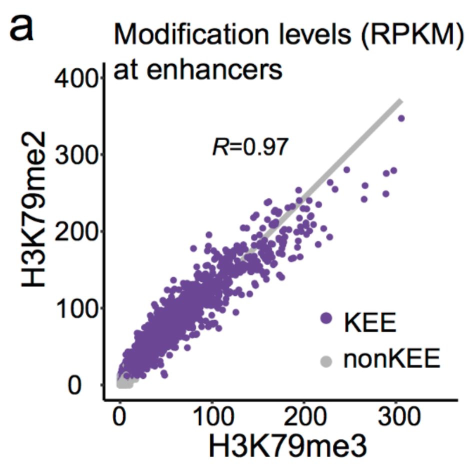

Figure 1. (a) Correlational analysis between H3K79me2 ChIP-seq and H3K79me3 ChIP-seq reads in SEM cells at KEEs (purple) and non-KEEs (gray), data based upon one replicate of both H3K79me2 and H3K79me3 ChIP-seq.

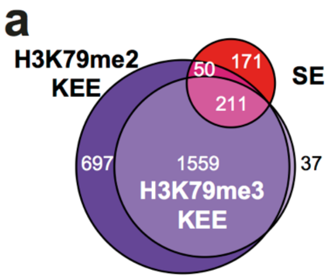

Supplementary Figure 1. (a) Overlap between KEEs identified using H3K79me2 and H3K79me3 ChIP-seq in SEM cells and super-enhancers (SE). Numbers inside the red circle reflect the number of super-enhancers within each overlap group.

In addition, we now have a new section in the discussion about this issue:

“In yeast, H3K79me2 and H3K79me3 display different distribution patterns {Schulze, 2009}, but past work in mammalian cells has suggested that these two modifications may overlap {Steger, 2008;Kerry, 2017}. Our analyses demonstrate a strong correlation between H3K79me2 and H3K79me3, suggesting that at least in MLL-AF4 cells they may be functionally equivalent. The H3K79me3 antibody used in our work here (Diagenode C15410068, lot A246-0040, recommended by the Blueprint Consortium for ChIP-seq) shows a high degree of specificity for H3K79me3 and not H3K79me2 in a dot blot analysis (http://antibody.uni-saarland.de/antibody/112/Diagenode_company/H3K79me3/), and the H3K79me2 antibody we use has been shown to be specific for H3K79me2 in ChIP experiments {Steger, 2008 }. This provides some level of confidence that our ChIP-seq results are not a consequence of antibody cross-reactivity, as has been noted for a different H3K79me3 antibody {Steger, 2008 }. However, since the nature of the antibody-epitope interaction differs between applications, we cannot completely rule out the possibility of cross-reactivity in our ChIP experiments.”

Reviewer #3 (Remarks to the Author):

This is a manuscript by Godfrey et al. entitled, “DOT1L inhibition reveals a distinct class of enhancers dependent on H3K79 methylation”. The premise of the article suggests that a distinct subset of enhancers in leukemia cells are functionally dependent upon H3K79me3 enrichment. The authors make use of the DOT1L inhibitor, EPZ-5676, emphasizing that the loss of H3K79me3 at these H3K79me3 enhancer elements (KEEs) leads to altered chromatin accessibility, histone acetylation and transcription factor binding. Followed by using a modified 3C approach, to show that H3K79me3 is essential for KEE interactions with the promoter as well as transcription of the associated genes. Overall, the concept is compelling; however, some weaknesses in the data presented make it difficult to clearly state whether H3K79me3 truly represent a subclass of enhancers. Nonetheless, there are some notable strengths to the approaches used and their results that suggest the significance of the H3K79me3 mark in gene control in leukemia cells as a potential hallmark of some enhancers.

We appreciate the work that the reviewer put into assessing our original manuscript and we are glad that they found our work to be worthwhile. We also appreciate the detailed comments the reviewer provided for our paper.

Among the authors’ main figures, they performed many comparisons however, several of these comparisons are not clear and lack rationale for their comparisons. There are a number of insignificant (or less convincing) plots (discussed below) that could be placed in Supplementary data. Whereas, having these figures within the main body of the manuscript detract from the authors’ overall hypothesis and make for a less convincing argument (i.e. ATAC-Seq, some controls, etc.), or preferred to just omit.

This reviewer’s belief is if the authors want to explore H3K79me3 functionality, it appears they have sufficient ChIP-Seq, RNA-Seq, and their modified 3C data to reach some modest conclusion for a gene regulatory role through H3K79me3 enrichment. However, the concept of KEE as a subclass of enhancers remains premature to claim from the data provided and for many of the figures the case for regulatory elements associated with a KEE is not made particularly strong.

The reviewer raises a very important point here, and we agree that it is premature to call KEEs a subclass of enhancers. We’ve changed the title of the paper to reflect this and no longer refer to KEEs as a class of enhancers. We’ve modified how we discuss the data

throughout the manuscript and temper our conclusions to reflect this, and removed reference to it being a subclass. In particular, we spend more time in the discussion developing the idea that H3K79me2/3 is mainly functioning to create a more “open” chromatin conformation (possibly through disruption of SIRT1 and increased H3K27ac levels) creating an opportunity for transcription factors to bind. Whether a specific sequence can then function as an enhancer depends on the repertoire of transcription factors that is present.

We have extended our initial analysis and have provided some new data to show that putative KEEs may be more widespread than we initially thought (Fig. 1b, c; see below). However, the reviewer is correct that all of our functional data is in MLL-r leukemia cells so it is not clear whether the KEEs we have identified in other cell types are functional enhancers.

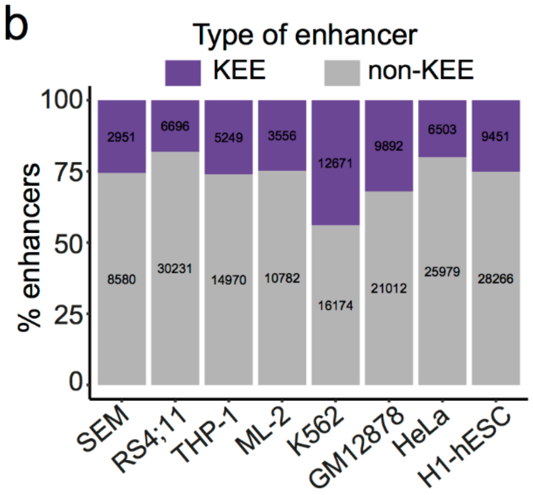

Figure 1 (b) Proportion of predicted enhancers which are KEEs (purple) or non-KEEs (gray) in different cell types, based on ChromHMM analysis.

To address the point of whether KEEs are functioning enhancers in more detail, we have now used CRISPR/Cas9 to delete parts of KEEs at *CDK6* and *ARID1B* and have shown that these deletions cause a reduction of gene expression in SEM cells (Fig. 2a; see below). In addition, we have shown that part of the *ARID1B* KEE and *JMJD1C* KEE can enhance luciferase expression in a luciferase assay (Supplementary Fig. 2a; see below). However, we have removed any reference to KEEs being a subclass of enhancers as cannot definitively conclude this.

a

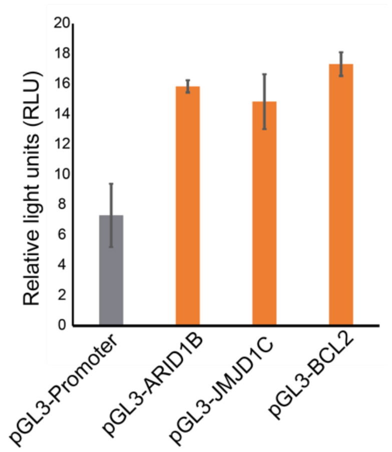

Supplementary Figure 2. (a) Luciferase assay for putative enhancer activity. pGL3 plasmid containing promoter alone, or sections of the *ARID1B* or *JMJD1C* KEE, or *BCL2* non-KEE were co-transfected with pRL-TK *Renilla* into 293T cells. Firefly luciferase activity was normalised to *Renilla* luciferase. Error bars represent standard deviation of three technical replicates.

a

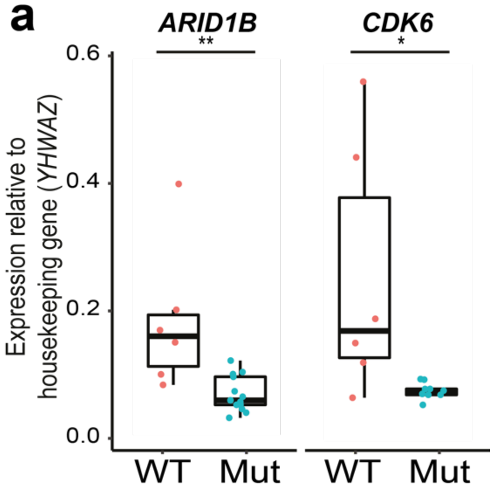

Figure 2. (a) Gene expression in wildtype compared to *ARID1B* and *CDK6* enhancer mutant SEM clones, normalised to the housekeeping gene *YWHAZ*. Each point represents a biological replicate. Mann Whitney U test, ** = p<0.05 based upon 7 biological replicates.

Specific Comments:

In Figure 1C: The figure notes that 2460 gene have the KEE signature in their enhancers, this apparently doesn’t necessarily indicate that "KEE may DIRECTLY regulate the expression of a subset of genes" as the authors state and claim. Combined with Figure 1D, the authors, again, use the characterization of “directly”. The KEE association in this instance maybe involved, but the use of “direct” is not an accurate characterization as presented.

This is a fair point and we have rewritten this section to explain the logic behind the approach we used to link enhancers and putative gene targets, and discuss the limitations of this method:

“We used the common approach of annotating each enhancer to the nearest gene, and using this to label genes as KEE- or non-KEE-associated (Supplementary Table 2). Many genes were associated with both KEEs and non-KEEs. For the purposes of our analysis,

KEE genes were defined as proximity to one or more KEEs (regardless of non-KEE association) and non-KEE genes were defined by proximity to one or more non-KEEs, but no KEEs."

Figure 1E and 1F: While the comparison between the KEEs with non-KEEs makes sense, the comparison of genes with a KEE versus those with no enhancer is not relevant. Then in Figure 1F, the authors divided each into three categories, high, medium and low H3K79me3 enrichment within a gene body which lack the appropriate context for comparisons, if their goal is about H3K79me3 enrichment at enhancer regions, having this information of H3K79me3 within gene body in this plot is very confusing and a poor comparison, and the mixture of these different gene contexts of those two patterns of H3K79me3 enrichment confuses the intent of the studies performed.

These are all very good points from the reviewer and we have attempted to clean up the presentation of the figure to make our point(s) clearer. In addition, since we have included some new analyses the main point of Fig. 1 has changed. We have removed most of the irrelevant analyses especially the "no enhancer" genes. Fig. 1a now compares H3K79me2 with H3K79me3 and shows that these two marks are highly correlated, at least in MLL-AF4 SEM leukemia cells. Fig. 1b and c are now focused on identifying putative KEEs in multiple cell types. Fig. 1d compares KEEs to non-KEEs in these different cell types to show that genes associated with a KEE tend to have higher expression. We also perform some further comparative analysis which we include in Supplementary Fig. 1. We hope this clarifies our intent and analysis.

Figure 1G: A KEE-containing gene, as is indicated in the name, already has the enrichment of H3K79me3 at their enhancers (most of which are intragenic, within the gene body, anyway), that means, the KEE-containing genes, are supposed to be enriched with higher levels of H379me3 in the gene body, regardless. Therefore, many of the plots within this figure panel are not relevant to the intended conclusion. While the box plot shows statistical significance, the differences do not appear dramatic between the KEE-containing gene and "no enhancer" groups. Also noted, H3K27ac and H3K4me1 -marked enhancers should also contain these two notable histone modifications in KEE and no-KEE containing genes.

Again, we have removed these comparisons as we think they are confusing and took away from the main point we are trying to make with the figure. The effect of DOT1Li on different histone marks at KEEs and non-KEEs is addressed more robustly later in the paper, where we specifically focus on H3K27ac.

Therefore, it is not clear what the authors are comparing among those enhancers with the dual H3K27ac/K4me1 reads versus those with “no enhancer also carrying the dual H3K27ac/K4me1 enrichments. This doesn’t help the authors with their argument.

We fully agree with the reviewer on this point and have taken out the no enhancer comparison.

The authors should consider removing Supplementary Figure 1A, as it is redundant with Figure 1b, and should be deleted. This may confuse readers.

We have removed both figures and replaced them with a single new Fig. 1c that highlights the genomic locations of KEEs in multiple cell types.

Shown in Supplemental figure 1C: Is it H3K79me in total the authors are measuring or is it a typo? Should it be H3K79me3? Besides, total methylation was not mentioned anywhere it in the text.

This was referring to both H3K79me2/3, but as this analysis was confusing and did not add anything additional we removed this to improve the presentation of the paper.

Supplemental figure 1 D: See a presumed typo: KEE gene, not “HEE”.

Thanks for catching this, this was corrected but we have now removed this figure.

On Page 6, In the end, the authors claim, “MLL-AF4 fusion spreading targets are enriched for KEES compared to non-spreading target”, The authors didn't indicate which specific plot this result was referring to in Supplementary Figure 1. The reviewer presumes Supplementary Figure 1G, but this figure lacks evidence for the “non-spreading” targets. Overall, MLL-AF4 study appears redundant, since the authors are not using the same cell line model for the remainder of the studies performed, and the only piece of evidence associated with their result is not convincing.

We have corrected this, and now refer to Supplementary Fig. 1d and e. We have also made it clear which cell types we have used for each figure throughout the paper. The bulk of the analysis has been in the MLL-AF4 SEM cell line, but where we have varied from this we have been explicit in the figure legends.

Shown in Figure 2D: The authors need to clarify what represents enrichment of the H3K79me3 mark within gene bodies and H3K79me3 within enhancers (or KEEs). The reviewer suggests that it would be more meaningful, if the comparison made are between KEE-associated versus non-KEE genes, but shouldn't involve genes enriched for H3K79me3 versus those lacking H3K79me3.

We agree with the reviewer that it is not meaningful to compare H3K79me2/3 marked genes (the presence of H3K79me2/3 within the gene body) to those that do not have H3K79me2/3. Treatment with DOT1L inhibitor potentially causes a lot of secondary gene expression effects. The purpose of this figure is simply to show that despite that, there is a strong correlation between the observed downregulation of targets and the mere presence of H3K79me2/3 within the gene (regardless of KEE/non-KEE status). We then go on to take all genes marked with H3K79me2/3 and explicitly divide them into genes that are associated with a KEE and those associated with a non-KEE, and show that KEE association itself is significantly correlated with downregulated gene expression upon loss of H3K79me2/3 (Fig. 2e and f).

Shown in Figure 2F: the pie chart is misleading, as the absolute values of KEE-containing, non-KEE, and the non-enhancer groups are drastically different (i.e. 2000~, 1300~, 3500~). Therefore, comparisons made using different absolute numbers, as shown, should somehow be better normalized to reflect the changes in percentage for each of these distinct categories.

We agree with the reviewer that comparing different absolute numbers is misleading. To clarify our approach here, what we have done is to perform the statistics on the proportions rather than the absolute numbers. To highlight this, we've added percentages to the figure as well. The Figure has now been move to 2e.

Shown in Figure 2h: it is apparent that not even KEE-containing genes have a decrease in gene expression (logFC). This result contradicts what the authors claimed.

The original Fig. 2h (now Fig. 2f) was a plot showing the average log fold change (FC) in gene expression in KEE, non-KEE genes and no-enhancer genes. The average fold changes are quite subtle by eye especially when we represent the full spread of the data as in the original figure. We have now replotted this data (with the no-enhancer genes removed) and instead show mean logFC, and it is much easier to see that KEE genes are

on average more sensitive to DOT1L inhibitor treatment than non-KEE genes (Fig. 2f; see below).

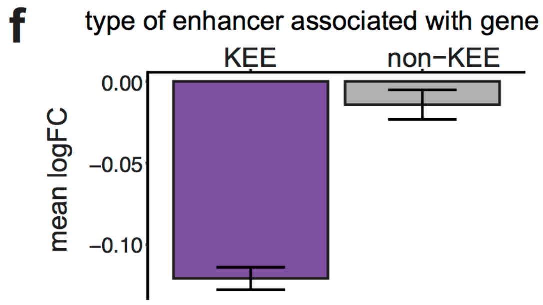

Figure 2. (f) Mean logFC of H3K79me2/3-marked genes associated with a KEE (purple) or non-KEE (gray) by nascent RNA-seq (**** = p-value<0.0001, Fisher's exact test, n=3).

However, the reviewer is correct in that the observed transcription changes are subtle. This something we have observed before when performing nascent RNA-seq experiments. One reason for this could be that since nascent RNA-seq is a more accurate reflection of actual transcription rates (compared to steady state RNA), the dynamic range is quite different as there is less variation in RNAPII transcription rates compared to steady state RNA accumulation. Such subtle changes in nascent RNA levels can often lead to strong changes in steady state RNA and ultimately protein levels, as we have observed in past studies (Kerry et al, Cell Rep, 2017)

Shown in Figure 2I it is not apparent why the authors measure K79me3 at the TSS, if it doesn't show results concordant with their hypothesis it shouldn't be presented. The authors make the statement, " K79me3 levels are the highest at KEE gene" in the text, which is not relevant to this plot shown (only represents KEE-associated genes localized within the TSS region). Also, the authors only measure the levels of H3k79me3 within the gene bodies (which lacks clear justification and should maybe included as supplementary data).

We agree with the reviewer that this analysis is confusing and not supportive of the aims of the paper and have removed it.

Shown in Figure 3b, the authors demonstrate only a 10% decrease in ATAC peak calls even for KEE-associated genes, therefore make the evidence presented weak. Furthermore, all the ATAC-Seq attempts to establish that H3K79me3 plays a fundamental role in chromatin accessibility needs more evidence to convince readers.

We agree with the reviewer that in general, the changes in ATAC-seq are subtle, but where decreases occur they tend to be enriched at KEEs (Fig. 3b). There are however some specific sites where the changes are more obvious. We have now combined the ATAC-seq data with the H3K27ac figure in traces at specific genes to compare them side by side, for example at *ARID1B* (Fig. 3b; see below). Some of the ATAC-seq analysis is now in Supplementary Fig. 3.

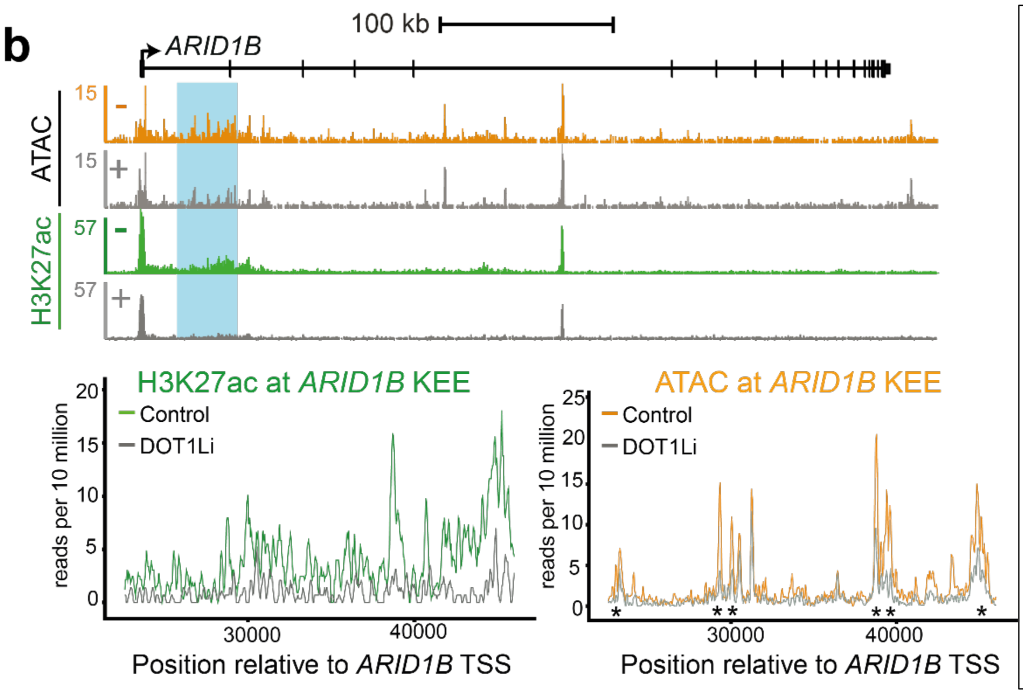

Figure 3. (b) Upper: ATAC-seq and H3K27ac ChIP-seq at *ARID1B* in control (-, orange/green) and DOT1Li (+, gray) SEM cells. Blue boxes highlight KEE cluster 1 region of *ARID1B*, shown in more detail below. Lower: Overlay of H3K27ac (left) and ATAC (right) signal at *ARID1B* KEE1 in control (green/orange) and DOT1Li (gray). Asterisks indicate significantly reduced peaks (FDR <0.05).

Shown in Figure 3 ei, the authors use the BCL2 gene as an example, which the authors neglected to mention in the text until describing in a later figure stating this gene is also slightly decreased. The authors should make some of this finding in the results section.

We agree this was not introduced sufficiently early in the original paper, so we introduce *BCL2* as a non-KEE gene earlier in the paper and discuss its relevance as a comparison for KEE genes.

Shown in Figure 4c, the authors show the KEE-associated group that upon adding the DOT1L inhibitor, the comparison for H3K27ac enrichment and the ATAC profile is very confusing to the reader. Therefore, the concept of how the K27ac and K79me3 together orchestrate chromatin accessibility is not convincing. Maybe the authors should try not to conflate the two signatures within one profile and separate this as two distinct patterns each in different plots.

We agree with the reviewer that as presented, this is a very confusing figure. To address this, we have reorganized Figure 3 in the following manner. First we show that upon DOT1L inhibitor treatment the average H3K27ac signal is decreased specifically at KEEs (but not at non-KEEs) genome-wide (Fig. 3a). We show examples of the change in H3K27ac profiles at a KEE (*ARID1B*) and non-KEE (*LMO4*) gene (Fig. 3b-c), with other examples in Supplementary Fig. 3. We then analyse changes in ATAC-seq peaks, and show that peaks that are decreased upon DOT1L inhibition are much more likely to be associated with downregulation of gene expression when found within with a KEE (Fig. 3d). Again, we use *ARID1B* and *LMO4* to highlight the difference in ATAC response at KEEs and non-KEEs (Fig. 3b-c). Much of the ATAC-seq analysis is now in Supplementary Fig. 3, and the main focus of Fig. 3 is on H3K27ac as that is the major enhancer attribute that displays a change. We have moved the specific figure referred to by the reviewer (Fig. 4c) to Supplementary Fig. 3i, and we now focus on H3K27ac levels at decreased ATAC peaks, for clarity.

Shown in Figure 5e, shown within the bar graph, only the ELF1 group in ARID1B are indicated as statistically significant shown with asterisks, however, it is not clear whether the H3K27ac, H3K4me1 and H3K79me3 group for this gene reach significance. This is especially important for illustrating the enrichment for H3K27ac, as the authors claim this being significant in Figure 4d. This detracts from the authors' claim.

We have now reorganized this figure to clarify our results and focus on the key findings. We show ELF1, H3K79me3 and H3K27ac ChIP qPCR at the KEE gene *ARID1B* and the non-KEE gene *LMO4* in Fig. 4, as well as several other example loci in Supplementary Fig. 4. The reduction of ELF1 binding at KEEs is novel and the H3K79me3 and H3K27ac results validate the ChIP-seq results. These experiments represent at least 5 biological replicates so that we can perform a Mann Whitney U test on the experiments and assign p values to each experiment (the assumption is that ChIP data does not have a normal distribution so a T-test is not appropriate here). We observe that there are significant changes in H3K79me3 and h3K27ac at KEEs but not at non-KEEs, which matches with our ChIP-seq experiments. For completeness, in Supplementary Fig. 4 we also present H3K79me2, H3K4me1 and H3K9me3 ChIP qPCR. H3K79me2 shows the same trend as H3K79me3 and thus matches the ChIP-seq data, and H3K4me1 does not appear to change. H3K9me3 shows a trend towards increasing which is consistent with past published results on H3K9me3 levels in DOT1L inhibitor treated cells {Chen, 2015, Nat Med}.

Shown in Figure 6E the authors show among the downregulated group of genes, BCL2, which is a non-KEE gene throughout the manuscript, the plot for BCL2 has circle dots

representing KEE enhancer, shouldn't this BCL2 gene be excluded from having any KEE enhancer? Otherwise, having at least one KEE enhancer suggests BCL2 is a KEE-associated gene. Is this not correct? If this is the case throughout the whole manuscript, the authors are using flawed negative control as a "non KEE" gene, therefore, making all of those comparisons improper. Is there an explanation for this?

We appreciate the reviewer bringing up this important point. It is true that *BCL2* represents a gene that has both a KEE and a non-KEE annotation. Mainly, it seems to have a large non-KEE that partially overlaps with H3K79me2/3 creating a partial KEE (see Supplementary Fig. 1n, below, tan shaded region). It is also likely that *BCL2* is not the only gene like this.

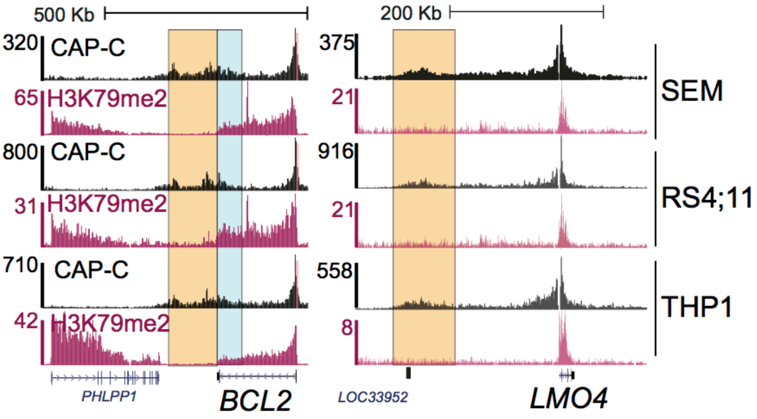

Supplementary Figure 1. (o) Capture-C and H3K79me3 ChIP-seq in SEM, RS4;11 and THP1 cells at *BCL2* and *LMO4.* Blue boxes represent KEE regions, orange boxes represent non-KEE regions.

Work from us (Supplementary Fig. 2a, see above) has shown that the *BCL2* non-KEE can function as an enhancer in a luciferase assay, and work from others has shown that deletion of this region can reduce *BCL2* expression {Tzelepis, 2018 Nat Comm}. Together, this suggests that the non-KEE is an important enhancer regulating this gene. However, loss of H3K79me2/3 at *BCL2* does cause downregulation of the gene, likely due to the role of H3K79me2/3 within the gene body in transcription elongation. What this example highlights is that although a genome-wide analysis is useful for categorizing regulatory events in broad strokes, specific loci have to be analysed in detail in order to truly get a clear picture of how a gene is being regulated. Our final analysis of *BCL2* is that it does not overall display significant changes in enhancer-promoter interactions across the enhancer upon DOT1Li in either SEM or RS4;11 cells (Supplementary Figure 5). However, as the reviewer noted, there are some "purple dots" (Figure 6a Supplementary Figure 6c) that do show some significant changes, likely due to some individual fragment interaction changes from the partial KEE that exists at the locus. We discuss this in this section of the results"

"In the more complex example of *BCL2*, which appears to contain both KEE and non-KEE enhancers, no significant disruption of enhancer-promoter interactions overall is observed in SEM, RS4;11 or THP1 cells (Supplementary Fig. 5d, Supplementary Fig. 6a, b) suggesting that this enhancer-promoter interaction is not dependent on H3K79me2/3. However, there are some smaller fragments of KEE loss of interaction as visualized in the bubble plots (Figure 6a and Supplemental Figure 6c), indicating that something more subtle may be occurring at this locus."

We have spent more time discussing the complexities of *BCL2* in the manuscript in both the results and discussion sections and have highlighted these sections.

In summary, the reviewer suggests the authors reorganize their figures and re-prioritize some data, and to remove unnecessary data, which further weaken the manuscript or at the very least embed such ancillary data within supplementary information if it can be justified for their use.

We believe we have taken into account all of the reviewer's comments and have extensively reorganized and rewritten the paper and we hope that the reviewer finds the overall message to be much more clear. We'd like to thank the reviewer for their careful comments and for helping us to improve the paper.

REVIEWERS' COMMENTS:

Reviewer #1 (Remarks to the Author):

The authors have been quite responsive to the comments and have enhanced the manuscript with further data and clarification. I have no further comments.

Reviewer #2 (Remarks to the Author):

The authors have fully addressed my initial questions and comments. This is now an excellent manuscript.

Reviewer #3 (Remarks to the Author):

The premise of the article suggests that a distinct subset of enhancers in leukemia cells are functionally dependent upon H3K79me3 enrichment. The authors make use of the DOT1L inhibitor, EPZ-5676, emphasizing that the loss of H3K79me3 at these enhancer elements (KEEs) leads to altered chromatin accessibility, histone acetylation and transcription factor binding leading to alterations in DEGs. Followed by using a modified 3C approach, to show that H3K79me3 is essential for KEE interactions with the promoter as well as transcription of the associated genes.

The main contention was whether the authors could provide stronger evidence of the functionality of these so called KEEs where some problems were with making observations apparently unsupported with strong evidence and using too many inconclusive and confusing figures to support the authors' claims. However, the authors have temper some of these observations and in line with both new and old data they now present. The authors appear to have removed many of the discrepancies noted in the prior review that made the manuscript somewhat garbled in places and inconsistent in others. This was specifically noted in the previous Figures #2 and #3 and made changes to the text to reflect these concerns noted by all the prior reviewers. Figures tend to be easier to understand now.

With regard to the revision, the authors performed more ChIP-seq with additional and different cell lines to keep their conclusion to become more broadly accepted and not entirely reliant on a specific a narrow tissue or cell type context. A question the authors address with some, but minimal, satisfaction is whether these "KEEs are really functional"? By performing CRISPR-based KO of some of the KEE elements questioned the authors ventured to identify chromatin and transcript changes that correspond to validate their tests but still seems a narrow window of responses. The authors attempted to revise their original DOT1L inhibitor treatment figure in a manner that is acceptable as shown in Figure 3, however, what they are trying to show is "KEE and its associated genes are more sensitive and responsive to the manipulation of H3K79me modification. This is still a little vague from the data presented but it is acceptable as changes are still somewhat modest. The authors do better explain about the H3K79me2 mark as well and giving KEE and KEE-associated genes thereby making a clearer definition from the previous version of this manuscript.

Overall, the manuscript is better written and more clearly consistent with the concept of the H3K79me mark as a putative enhancer mark, and what authors present are more in line with what they claim. There remain some minor concerns over the novelty of the study since other reviewers mentioned this relationship before with H3K79me2 being addressed elsewhere, but overall make a stronger case for addressing their hypothesis and could be considered as a stronger manuscript.
